# A Holistic Approach to Determining Stereochemistry of Potential Pharmaceuticals by Circular Dichroism with β-Lactams as Test Cases †

**DOI:** 10.3390/ijms23010273

**Published:** 2021-12-27

**Authors:** Marcin Górecki, Jadwiga Frelek

**Affiliations:** Institute of Organic Chemistry, Polish Academy of Sciences, Kasprzaka 44/52 St., 01-224 Warsaw, Poland; jadwiga.frelek@icho.edu.pl

**Keywords:** stereochemistry, absolute configuration, conformation, circular dichroism, β-lactam

## Abstract

This paper’s main objective is to show that many different factors must be considered when solving stereochemical problems to avoid misleading conclusions and obtain conclusive results from the analysis of spectroscopic properties. Particularly in determining the absolute configuration, the use of chiroptical methods is crucial, especially when other techniques, including X-ray crystallography, fail, are not applicable, or give inconclusive results. Based on various β-lactam derivatives as models, we show how to reliably determine their absolute configuration (AC) and preferred conformation from circular dichroism (CD) spectra. Comprehensive CD analysis, employing both approaches, i.e., traditional with their sector and helicity rules, and state-of-the-art supported by quantum chemistry (QC) calculations along with solvation models for both electronic (ECD) and vibrational (VCD) circular dichroism ranges, allows confident defining stereochemistry of the β-lactams studied. Based on an in-depth analysis of the results, we have shown that choosing a proper chiroptical method/s strictly depends on the specific case and certain structural features.

## 1. Introduction

Unambiguous assignment of the absolute configuration (AC) of compounds isolated from natural sources or synthesized in laboratories continually remains essential in chemistry, biochemistry, structural biology, and medicinal chemistry. It is crucial for synthetic and natural product chemists wishing to characterize their products fully but mandatory for nanotechnology, modern chemical, and pharmaceutical industries. Correct AC determination may become critical for pharmaceutical applications, as almost half of the active pharmaceutical ingredients (APIs) are chiral. Biological properties of chiral molecules, in turn, are directly related to their three-dimensional (3D) structure and therefore call for easier access to methods allowing reliable assignment of the stereostructure to ensure the good quality, safety, and efficacy of potential drugs. Consequently, the AC must be established with the highest degree of certainty as chiral drugs’ enantiomers may exhibit null, similar, different, or opposite therapeutic activity. Thus, different biological properties of a pair of enantiomers may result in the marketing of both isomers with different therapeutic indications in their enantiopure forms. Moreover, a racemic mixture of a drug or its scalemic form may exhibit different properties than the individual enantiomers, providing the best therapeutic profile in a given case. Therefore, eliminating the undesired stereoisomer from a drug’s preparation and determining its optimal dose and purity may become essential for assuring proper therapy [1,2,3]. In this context, the unambiguous assignment of AC, conformation, and optical purity of biomolecules inquired becomes extremely important. At present, the single-crystal X-ray diffraction method is considered to provide the most definitive results when assigning stereochemistry. However, the primary requirement for its use is to have a properly diffractive crystal, which cannot always be fulfilled. Even with suitable crystals, examples of incorrect absolute configuration determination based on X-ray crystallography alone or crystallographic results needing support by other methods can quite often be found in the literature [4,5,6,7]. One of the reasons may be more ready crystallization of an unrepresentative component of the bulk material [8]. In such a case, validating crystallographic stereochemical assignments may be achieved by analyzing many crystals and a statistical judgment of the consistency of results. However, such an approach is often impossible because of the sample’s lack of, or its poor, crystallization. For chiral compounds, another option of assignment confirmation is a comparison of circular dichroism (CD) curves obtained from measuring a single crystal solution previously analyzed crystallographically and a solution of a representative portion of the bulk sample. Thus, by providing complementary data for determining stereochemistry and optical purity, as well as by offering insight into chiral phenomena, CD authenticates its role as a method of choice for elucidating chirality and, in particular, for monitoring and characterizing even the smallest changes of a molecule in solution and solid phase.

In recent times CD, in its electronic and vibrational ranges (ECD and VCD, respectively), is becoming an increasingly important and productive research tool because it effectively determines the absolute configuration, conformation, and optical purity of chiral molecules. This is evidenced by, for example, the constantly growing number of literature reports on its practical and successful use, such as these few newest links indicated here [9,10,11,12,13,14,15,16,17].

Moreover, the incorporation of VCD as a new standard method in US Pharmacopeia in 2016 resulted in a faster understanding of a potential drug’s bioactivity by defining its structure and purity as soon as possible, thus allowing quicker entry into the market and a cost reduction when obtaining the drug [18]. Especially beneficial in this regard is the combined application of both of these methods—ECD and VCD—as it can provide in-depth, detailed stereochemical information about studied molecules and substantially increase the credibility of the assignment [15,19,20]. Consequently, two theoretically assisted chiroptical techniques are highly recommended for routine use as, in some cases, this is the only way to identify the spatial structure correctly [15,21,22,23,24,25]. However, relatively often, one can come across cases when the combined use of ECD and VCD is impossible or ineffective [26]. Such circumstances are mainly driven by limitations of the given method and the structural complexity of samples tested and/or their interactions with the solvent. In such cases, choosing the right method that guarantees error-free conclusions while increasing the reliability of the assignment is crucial. This is especially vital for drugs for which incorrect stereochemical assignments may cause unpredictable effects, including severe consequences.

Among structural factors determining the method of choice to be applied, the key roles performed include conformational freedom of the sample, the presence of large substituents in its backbone, including their impact on the molecular geometry, the number of chromophores present in the molecule, and their interactions. Substituents of steric bulk, especially with additional stereogenic centers and electron-donating or withdrawing groups, can effectively obstruct spectral analysis by VCD [27]. This is because VCD measures the vibrations of the molecule’s entire skeleton and the impacts associated with the volume occupied by the substituent and its conformational freedom accumulated. Therefore, they significantly affect calculations, at least because of their time consumption and accuracy. In contrast, in ECD, due to the analysis being limited to the chromophoric system and its immediate environment, problems associated with large substituents do not cause such severe complications in calculations despite using the same conformers pool in ECD and VCD [11].

**Chart 1:**
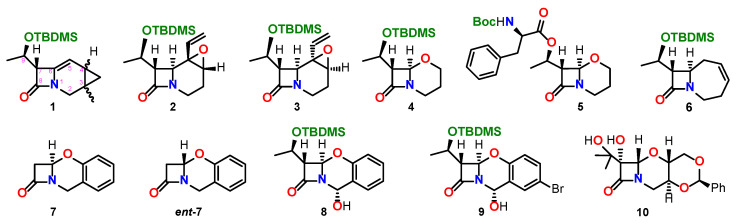
Structures of compounds of interest in the present study.

This paper is intended to demonstrate that the successful use of ECD, VCD, or their combination depends on the problem to be solved. Particular emphasis will be placed on confirming the effectiveness of CD spectroscopy in answering structural problems, especially in cases where the solution proposed by other methods does not always yield precise results or is impossible entirely. Based on the model of β-lactams collected in Chart 1, we intend to show that even slight differences in the starting molecular backbone can cause differences in the chiroptical method’s effectiveness and significantly affect the bioactivity leading, for example, to an improved or completely new biological profile.

β-lactams were selected as models because of their remarkable antimicrobial properties, making them one of the most clinically relevant medicines [28,29]. Model compounds, thoughtfully selected from a large collection of β-lactam derivatives synthesized in our research group (for details see the section: Source of compounds, and Supplementary Materials), cover their various classes, including carbacephams (**1**–**3**,**6**), oxacephams (**4**,**5**), and related unconventional bicyclic systems containing medium-sized rings fused to the β-lactam nucleus (**2**,**3**,**7**–**9**). Among the latter, greatly interesting, although little studied so far are 3,4-benzo-5-oxacephams (**7**–**9**) possessing two pharmacophoric cores in their backbones, i.e., azetidinone and chromane, resulting in the creation of a shared 1,3-benzoxazine subunit. Combining azetidinone and chromane structural fragments in one molecule, each with independent, significant biological activity, makes tricyclic cephalosporin oxanalogs **7**–**9**, now referred to as oxabenzoxals, which are exciting models for systematic chiroptical and biological research. Our preliminary results on the latter subject demonstrated that their representatives **7** and **9** act as DDpeptidase 64–575 inhibitors with an IC50 at the level of mmol/L [30], implying the possibility of obtaining new valuable molecules with a broader spectrum of action via further structural modifications. Therefore, the correct assignment of AC at the bridgehead carbon atom responsible for the antibiotic activity of β-lactams [31], as well as newly created stereogenic centers resulting from structural changes, is of particular importance. From the CD spectroscopy point of view, in turn, the presence of a complex chromophoric system in skeletons makes these compounds a spectroscopically stimulating research object. For example, the helicity rule’s effectiveness linking O = C-N-C helicity with AC of the bridgehead carbon atom generally working for β-lactam systems and the possible interaction between chromophores may need to be carefully examined [32,33]. In terms of the CD spectra interpretation, lactam **5** is also an exciting example due to possible inter-chromophoric interactions that could significantly hinder the analysis.

Secondly, but no less important, conformational effects are also an essential factor of biological activity, and their study is also possible through dichroic studies. Consequently, for the present research, we selected compounds differing significantly in their conformational flexibility. In line with this, a rigid three-membered skeleton characterizes tricyclic β-lactams **1**–**3** and **7**–**9** (Chart 1), while the conformational lability of common scaffolds in bicyclic lactams **4**–**6** increases in ascending order of numbering. What is more, the presence in β-lactams **1**–**4**,**6**, and **8**–**9** of the same protecting group with an additional stereogenic center, i.e., *tert*-butyldimethylsilyl (TBDMS), allows for studying the impact of this bulky structural motif on CD spectra.

The set of discussed examples is closed by the β-lactam derivative **10**. Its AC at the bridgehead carbon atom was definitely established as (6*S*) by NMR and ECD spectroscopies [34,35]. However, assigning AC to the C7 carbon atom required applying another ECD method, known in the literature as the in situ dimolybdenum method. The effective use of this methodology to determine the stereochemistry of the *tert*/*tert vic*-diol moiety will be presented in detail with the example of this compound [35].

This paper is organized as follows. We will start with a brief discussion on selecting computational methods and their use because quantum chemical computations play a critical role in stereochemical studies by providing a theoretical basis for experimental results. Next, we will discuss the most convenient and effective ways of determining the AC of test compounds according to the increasing degree of interpretation difficulty. Accordingly, we will first present rigid compounds or compounds with significantly reduced conformational lability. Subsequently, we will consider compounds of growing skeletal flexibility paying attention to the impact of other chromophoric systems and bulky substituents on the CD spectra. Finally, we will denote the strengths and weaknesses of each of the approaches presented and indicate the challenges and prospects for further development of chiroptical methods.

## 2. Results and Discussion

### 2.1. General Information on Calculation Methods

A fundamental requirement for the effective computational calculations of any chiroptical properties is the prior knowledge of all relevant conformational species of the respective molecule [36]. From a technical point of view, the simulation of CD spectra of any molecules comes down to three main steps:

(1) Conformational search.
(2) Calculating the CD spectra of conformers found.
(3) Calculating the Boltzmann average of the CD spectra of single conformers to compare the final CD spectrum with the experimental one.

Conformational analysis is the fundamental step, as it allows for identifying a set of possible conformers on the potential energy surface (PES) of a molecule, i.e., determining the most stable conformations and their energy. Since CD is sensitive to any minimal conformational fluctuations, this stage is crucial, but on the other hand, it can generate the most errors, which in the extreme case could make the assignment erroneous. That is why it is essential to conduct conformational analysis carefully and in the broadest energy range possible: Generally, it is 3 kcal/mol for rigid molecules and 5–10 kcal/mol for conformationally labile ones. Next, conformers obtained after optimization at the molecular mechanic level must be re-optimized at the DFT level to refine the geometry and find relative energy between them. Then, the most abundant conformations obtained are submitted for the simulation of CD spectra. The final simulated CD spectrum for the test compound is obtained by Boltzmann averaging spectra of all contributing conformers. Based on the comparison of the simulated spectrum with the experimental, an assignment is made. However, when comparing theoretical and experimental spectra, it is recommended to use the similarity factor (SF) to obtain a quantitative assignment and increase its reliability [37,38]. Including solute–solvent interactions in the calculations using an appropriate solvent model also significantly helps predict conformer populations and simulates chiroptical properties.

In order to demonstrate proper and critical use of a holistic approach when necessary to determine the stereochemistry of chiral compounds in each analyzed compound, we initially performed a two-step conformational analysis in the range of 10 kcal/mol. The first step of this analysis was carried out at the molecular mechanics level (MMFF94s force field), and for the next one, optimizing conformers obtained in the range of 5 kcal/mol, the DFT method was used at the ωB97X-D/6–311+G(d,p) theory level with the PCM solvent model. Subsequently, we used the obtained pool of the most abundant conformations to simulate the chiroptical properties. To ensure that the obtained results are comparable, we also simulated the electronic and vibrational CD spectra at different levels of approximation; however, for the discussion here, we selected only the ones that demonstrate the best fit with the experimental spectra, i.e., obtained at the ωB97X-D/6–311+G(d,p) level of theory with the PCM solvent model. Additional motivation for using this approximation level was prior identification of its effectiveness and accuracy in reproducing chiroptical spectral features of various organic molecules of synthetic and natural origin [26,39,40,41,42,43,44]. In our previous work on β-lactams, we also showed that using the PCM solvent model significantly impacted the assignments’ credibility [22,31,33,45,46,47].

To reflect the ECD and/or VCD spectra’s measurement conditions, we ran DFT optimizations and simulations of CD data under the theoretical conditions specified above for compounds **1**–**7** in CH_3_CN (ε = 35.69) and **8** and **9** in both CH_3_CN and CHCl_3_ (ε = 4.71). The choice of CHCl_3_ was dictated by the solubility of these compounds in the concentration required by VCD. We also performed ECD calculations using CAM-B3LYP and B3LYP functionals, but their results were less satisfactory than for the previously mentioned ωB97X-D functional.

In order to quantify the last step of the stereochemical analysis, the comparison of theoretical and experimental data was analyzed using the similarity factors (SF) in each studied case. Moreover, in Table 1, we have listed the CPU times for ECD and VCD simulations to gain insight into the computational time and cost aspects of calculations related to obtaining the best quality/time ratio for all test compounds.

The data collected in the table additionally show both the economic and practical roles of choosing the right method for a specific case. They also point very clearly that the geometry optimization time strongly depends on the quality of the starting geometry.

### 2.2. Solving Stereochemical Problems Step by Step

The first stereochemical problem we intend to analyze is determining the AC at C3 and C4 carbon atoms in β-lactam derivative **1** (Chart 1). This compound, a representative of the carbacephem family, was obtained in a few-step synthesis starting from commercially available Kaneka azetidinone, i.e., (3*R*,4*R*)-4-acetoxy-3-(*R*)-(tert-butyldimethylsilyloxy)-ethyl)-2-azetidinone (i) according to the literature procedure [48].

**Figure 1:**
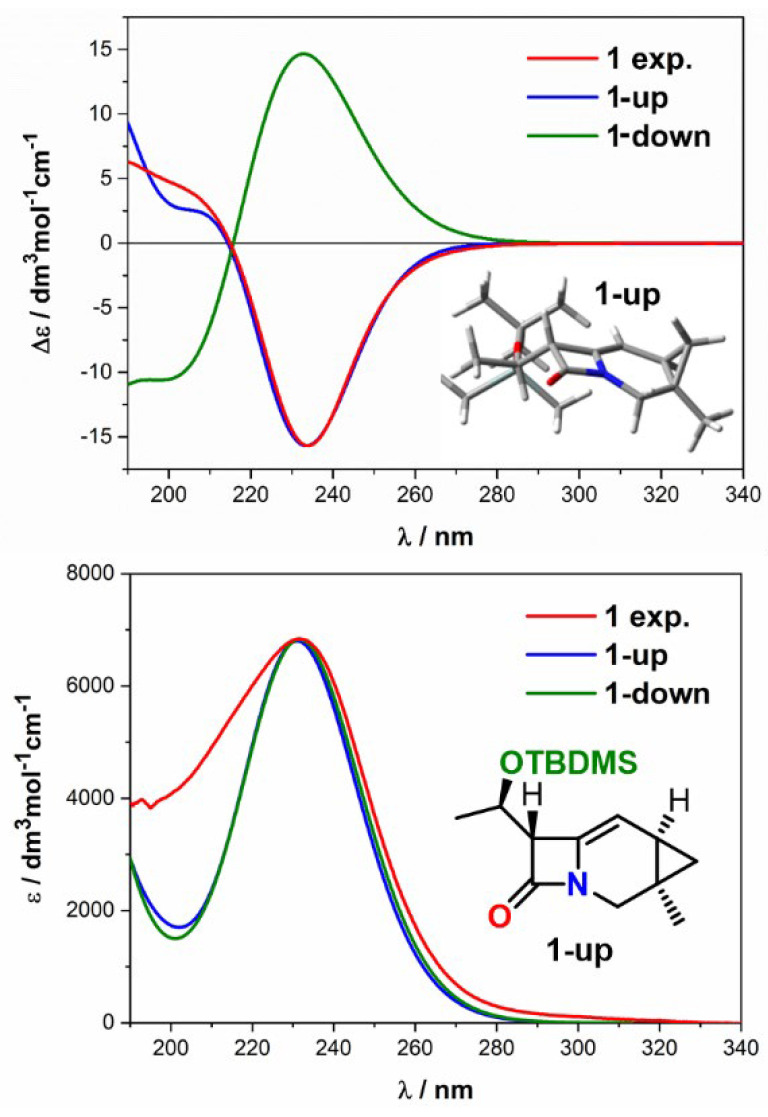
Comparison of computed ECD (**top**) and UV (**bottom**) spectra of (3*R*,4*R*) and (3*S*,4*S*)-diastereomers of carbacephem **1**, termed **1-up** and **1-down**, respectively, with experimental one recorded in CH_3_CN. The lowest energy conformer of (3*R*,4*R*)-diastereomer of **1-up** is presented on the top panel. Calculated ECD spectra were obtained with 0.42 eV exponential half-width, red-shifted by 13 nm.

This tricyclic carbacephem **1** constitutes a highly strained system consisting of fused four, six, and three-membered rings with conformational lability restricted mainly to the side-chain substituent at the C7 carbon atom. Its relatively unrestricted mobility is evident from results of conformational searches showing, for both diastereoisomers (later referred to as **1-up** and **1-down**), the presence of three conformers in the 1 kcal/mol energy window. In the lowest energy conformers of both diastereomers, the four- and six-membered rings are nearly planar, and the cyclopropane ring is directed, depending on diastereomer, above or below this plane (Figure 1) [48]. Diastereoisomers that differ only in stereochemistry on two adjacent carbon atoms (here C3 and C4) should exhibit opposite signs of Cotton effects (CEs) in ECD since the different orientation of the cyclopropane ring disturbs the cephem chromophore differently. Thus, distinguishing such diastereoisomers should not be difficult, making ECD the first-choice method for compound **1**. To verify this assumption, we simulated ECD spectra for both diastereomers using quantum chemical calculations (QC) and compared them with the experimental spectrum of **1**. The Boltzmann-weighted average ECD spectrum of carbacephem **1-up** shows very close agreement with the experiment (SF = 0.991), thus providing clear evidence for the (3*R*,4*R*) absolute configuration (Figure 1). The simulated UV spectrum of compound **1-up** also shows good agreement with the experimental spectrum, while **1-down** lacks such a good match. Thus, the combined experimental and theoretical analysis of ECD results enabled assignment (3*R*,4*R*) AC in **1** with a high degree of certainty.

**Figure 2:**
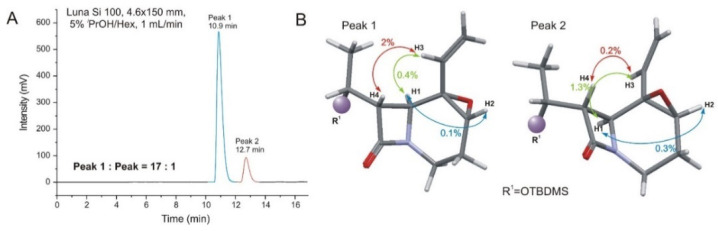
(**A**) An HPLC chromatogram for separation of epoxides **2** and **3** mixture on Luna^®^ (Si100, 3 μm) column, eluent 5% ^i^PrOH in hexane, flow rate 1 mL/min; (**B**) NOE experimental results for **2** (peak 1) and **3** (peak 2). The arrows indicate the observed NOEs upon irradiations as percent enhancement of ^1^H NMR signals.

The structural analysis of spontaneous epoxidation products of 5-vinyl-1-azabicyclo [4.2.0] oct-4-en-8-one, namely epoxides **2** and **3** (Chart 1) proved to be much more complex. The starting diene self-epoxidation reaction under aerobic conditions yields a mixture of two epoxides **2** and **3** in a ratio of 17:1 with an accompanying 25% decomposition of the diene after three days at room temperature (for details see Supplementary Materials). The first step to fully characterize the epoxidation products was their separation by HPLC using the Luna Si100 (150 mm × 4.6 mm, 3 μm) column and 5% of ^i^PrOH in hexane as a mobile phase with a flow rate of 1 mL/min. Under these conditions, both mixture components were isolated in pure form at a retention time for peaks 1 and 2 of 10.9 min and 12.7 min, respectively (Figure 2A).

Having both stereoisomers at our disposal, we used the NOE experiment to determine the epoxide’s AC, i.e., at C4 and C5 carbon atoms, and assign the appropriate structure to both epoxides. The irradiation of H1 and H4 protons in the sample corresponding to epoxide **2** caused the interaction between H3 and H4 protons. No NOE interaction between H1 and H2 protons indicated that H3 and H4 protons are in spatial proximity at the upper side of the β-lactam ring (Figure 2B). In contrast, the irradiation of H1 and H4 protons in epoxide **3** caused a strengthening of H1 and H3 protons’ signals and the lack of interaction with the H4 and H2 protons, indicating the spatial proximity of the H1 and H3 protons. It also demonstrated that protons H2 and H4 are on opposite sides of the molecule. Thus, based on the NOE interaction between H3-H4 and H1-H3, (4*R*,5*S*) AC could be assigned to epoxide **2** and (4*S*,5*R*) to epoxide **3**.

**Figure 3:**
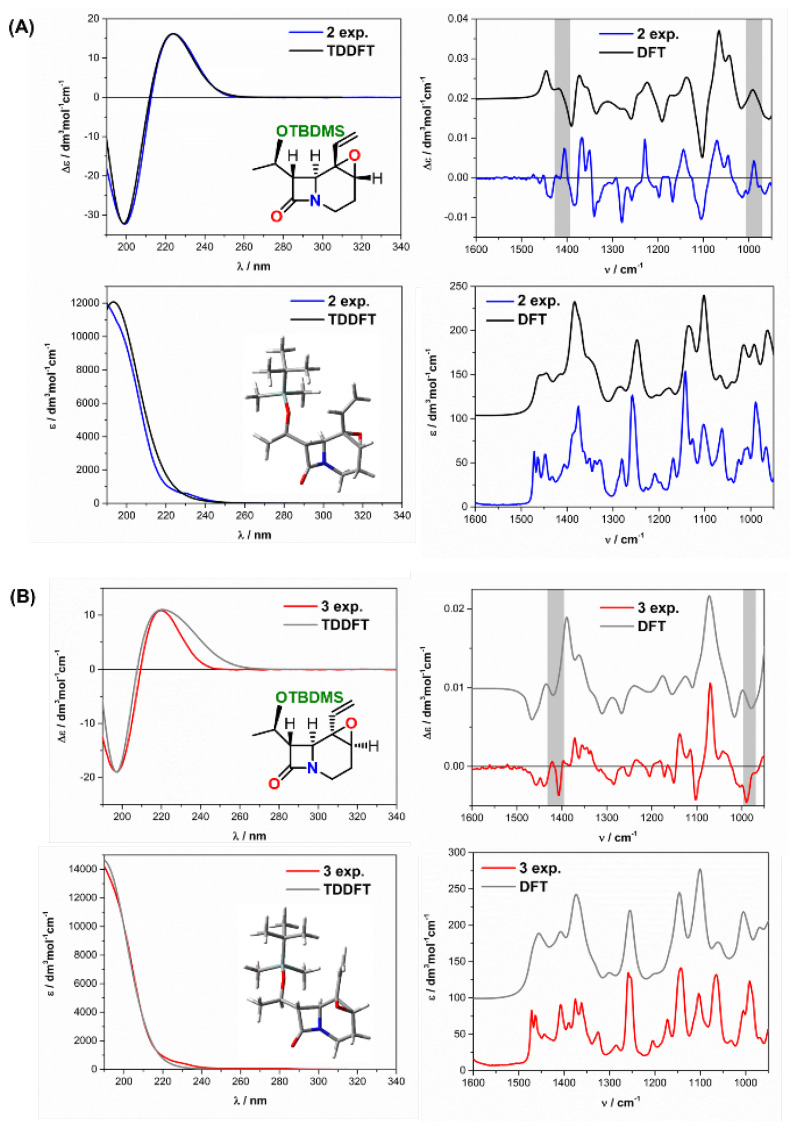
The experimental and computed ECD/VCD and UV/IR spectra of epoxides **2** (**A**) and **3** (**B**). Calculated ECD spectra were obtained with 0.47 eV exponential half−width; red−shifted by 20 nm.

To strengthen the NOE assignment with independent proof, we used CD spectroscopy in the next step. As it turned out, combined experimental and theoretical ECD did not provide a sufficient distinction between diastereomeric isomers. Positive CE, which appeared in both samples at around 220 nm, confirmed the (*R*)-AC of the bridgehead carbon atom in compliance with the helicity rule developed for cyclic β-lactams [32]. However, it did not resolve the AC of C4 and C5 carbon atoms due to the apparent similarity of experimental and simulated ECD curves for both (4*R*,5*S*) and (4*S*,5*R*) isomers (Figure 3A,B, left). However, this distinction was made possible by the VCD. As can be seen in Figure 3A,B (right), in experimental VCD spectra, bands in the fingerprint area, i.e., at 1420 and 990 cm^−1^, show opposite signs and therefore are suitable for distinguishing diastereomers. The comparison of experimental and theoretical curves of assumed stereochemistry unambiguously indicates (4*R*,5*S*) AC for epoxide **2** and (4*S*,5*R*) for epoxide **3**. What is more, the VCD assignment based on compliance between experimental and simulated curves for the assumed AC is consistent with the NOE assignment. The full agreement achieved between experimental and simulated curves for the assumed AC proved to be consistent with the NOE experiments′ results. Thus, in this case, a reliable assignment of (4*R*,5*S*) and (4*S*,5*R*) AC for diastereomeric epoxides **2** and **3**, respectively, was made possible by VCD analysis.

The following examples, i.e., oxacephams **4** and **5** [49], represent systems with a relatively rigid bicyclic ring skeleton and significant lability of substituents located at the C7 carbon atom. Unlike oxacepham **4**, the side-chain substituent of oxacepham **5** bears two other chromophores, amide and phenyl, interfering with the β-lactam chromophore present in the molecule’s backbone and one more stereogenic center. Therefore, such a multi-chromophoric system is much more complex for CD analysis, especially VCD. This complexity is related to the overlapping absorption bands from more chromophores, an increased number of stereogenic centers, and a significant number of energetically close conformers. Moreover, the TBDMS substituent in compound **4**, although free of strongly absorbing chromophores, may also significantly affect the course of CD curves and hinder their interpretation due to its steric bulk, thus profoundly affecting the chiroptical properties.

**Figure 4:**
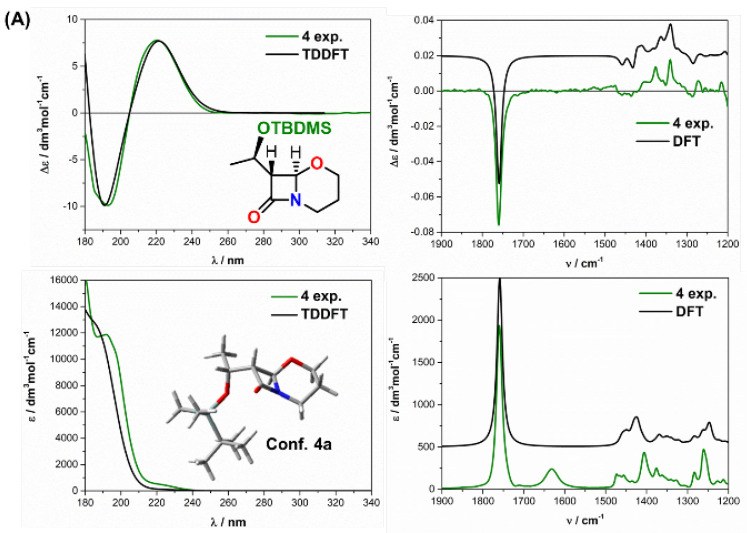

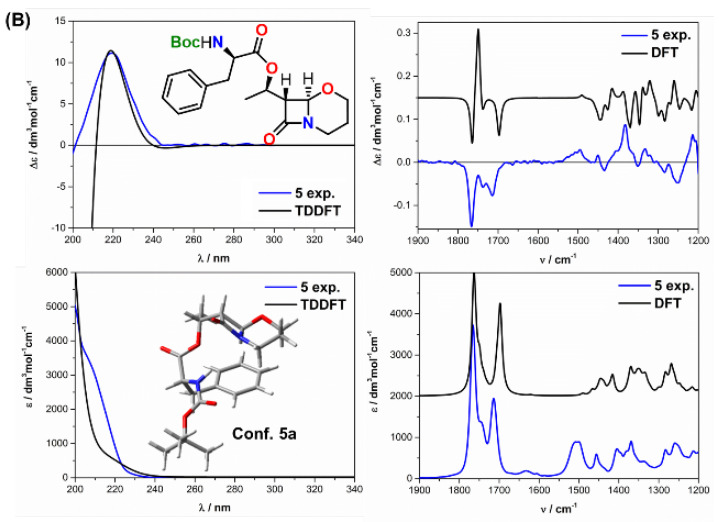
The experimental and computed ECD/VCD (top) and UV/IR (bottom) spectra of oxacephams **4** (**A**) and **5** (**B**). The lowest energy conformers 4a and 5a are presented in the appropriate inserts of the IR spectra. Calculated ECD spectrum for **4** and **5** were obtained with 0.44 or 0.42 eV, respectively, exponential half-width; red-shifted by 14 or 6 nm, respectively.

A positive CE at ca. 220 nm preceded by a negative one at around 195 nm characterizes experimental ECD curves of oxacephams **4**. In the case of oxacepham **5**, the short-wavelength negative band at ca. 195 nm does not evolve completely to a minimum precisely because of this complexity. Nevertheless, a well-developed positive maximum at around 220 nm in **5** lends itself well to interpretation (Figure 4B). According to the β-lactam helicity rule, the positive sign of the 220 nm CE, corresponding to the amide *n*→π* transition, correlates with the (*R*) AC of the C6 carbon atom [31,32,33]. Thus, based on the positive sign of this ECD band, one can assign (6*R*) AC to both oxacephams **4** and **5**.

In order to confirm the assignment based on the helicity rule, we simulated theoretical ECD curves for **4** and **5** and compared them with the experimental ones. In oxacepham **4**, the six-membered ring of the lowest-energy computed conformer adopts a chair conformation with the β-lactam ring in an energetically favorable equatorial position. Thus, at 300 K, the chair conformation of the pyranose ring dominates. The experimental and simulated ECD curves’ excellent compatibility confirms the (6*R*) AC and chair conformation of the heterocyclic ring and the helicity rule’s validity for this oxacepham. The above conclusion is additionally strengthened as the 220 nm band has the pure character of an amide *n*(O)→π* transition undisturbed by other contributions. Finally, it also means that the sterically hindered TBDMS substituent does not significantly affect the ECD curve of **4** (Figure 4A, left). The VCD spectrum of **4** (Figure 4A, right) also confirms the above assignment, as the similarity factor for the (6*R*) isomer accounts for 0.941, while for the (6*S*) epimer, only 0.004.

The situation changes drastically in oxacepham **5**. Due to the presence of groups strongly interfering with the amide chromophore in the C7 substituent, its ECD spectrum is much more complex than oxacepham’s **4**. These groups, including phenyl, absorb at similar wavelengths as the β-lactam chromophore, complicating the ECD spectrum. Although calculations reproduce the experimental spectrum in the 240–210 nm range well, the short-wavelength agreement is less satisfactory (Figure 4B, left). Nevertheless, the similarity factor (SF) for the (6*R*) isomer is 0.961. Thus, it provides a reasonable basis for assigning the (*R*) configuration to the C6 carbon atom.

The ultimate reason for the discrepancy between the experimental and calculated VCD and IR spectra of **5** is unclear. Usually, VCD provides in-depth knowledge of molecular interactions due to its higher sensitivity to local molecules’ changes [50]. Other oxacepham **5**-like cases in which stereochemical analysis using VCD fails in contrast to the ECD method are also known in the literature [26]. Nevertheless, the positive sign of the decisive ECD band in both experimental and Boltzmann-averaged ECD spectra of oxacepham **5** is very satisfactory. In the case of VCD, in turn, the situation is not so favorable as in ECD. Despite using the same conformers pool in the calculations, the VCD spectrum does not provide an unambiguous stereochemical assignment for **5**. The achieved similarity coefficients are 0.439 for the (6*R*) isomer and 0.114 for the (6*S*) epimer. Thus, despite the similarity factor being almost three times higher for (6*R*) than for (6*S*), it is still far too small to consider the assignment obtained on its basis as doubtless.

The ultimate reason for the discrepancy between the experimental and calculated VCD and IR spectra of **5** is unclear. Usually, VCD provides in-depth knowledge of molecular interactions due to its higher sensitivity to local molecules’ changes [50]. Other oxacepham **5**-like cases in which stereochemical analysis using VCD fails in contrast to the ECD method are also known in the literature [26]. Nevertheless, the positive sign of the decisive ECD band in both experimental and Boltzmann-averaged ECD spectra of oxacepham **5** and the very satisfactory agreement between both curves in the long-wavelength range corroborates the correctness of the ECD-based assignment for the C6 carbon atom.

**Figure 5:**
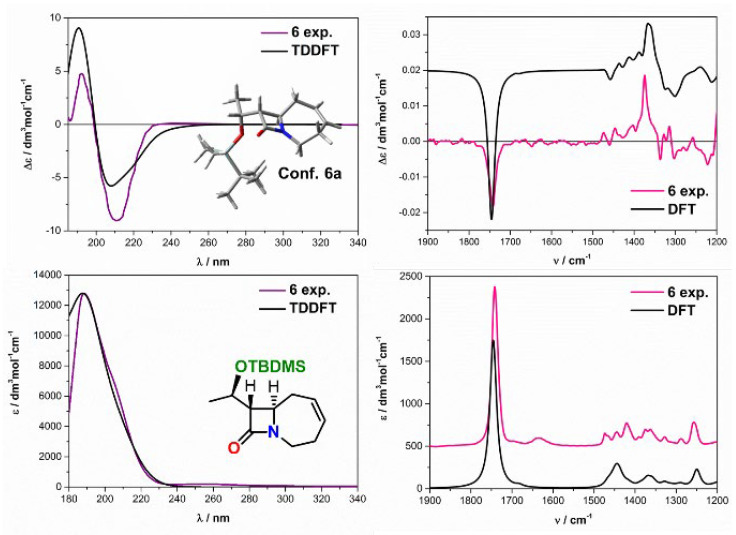
The experimental and computed ECD/VCD (**top**) and UV/IR (**bottom**) spectra of carbacephem **6**, including molecular structure and lowest energy conformer. Calculated ECD spectrum was obtained with 0.44 exponential half-width; red-shifted by 31 nm, respectively.

In carbacephem **6**, we are dealing with a similar but not as drastic situation as in oxacepham **5**. As shown in Figure 5 (left top), the simulated ECD curve is well-matched with its experimental counterpart. Thus, the negative CE sign at 211.5 nm is consistent with that already specified for non-classical β-lactam derivatives of the (7*R*) absolute configuration [46]. The band’s occurrence attributed to the amide *n*→π* excitation at 211.5 nm indicates a shift by around 10–15 nm to the higher energy region than typical carbacephams do [31]. This shift is likely related to the interfering ene chromophore, which absorbs at a similar energy range that the amide chromophore does. The calculations confirmed that the 211.5 mm band is a mixture of excitations from the *n*→π* amide transition and a double bond, strongly mixing mutually.

Some conformers of oxacephem **6** calculated in the range of 3 kcal/mol demonstrate a small deviation of the amide chromophore from planarity manifested by a slight pyramidality of the amide nitrogen and thus comply with the helicity rule. Nevertheless, as expected the decisive ECD band’s negative sign in both the experimental and Boltzmann-averaged ECD spectrum certifies the assignment and shows the amide helicity rule’s breakdown for this compound. Thus, in carbacephem **6**, the calculations-assisted ECD spectrum assigns (7*R*) AC of the C7 carbon atom.

A slightly different situation occures in VCD (Figure 5, right). Although the VCD spectrum correctly predicts the C=O amide stretching vibration at ~1742 cm^−1^, differences in the short-wavelength range of the spectrum cause the similarity factor to be equal to 0.871. Therefore, the assignment of AC on a VCD basis cannot be considered definite. Hence, as in oxacepham **5**, VCD does not provide a reliable stereochemical assignment here, most likely due to the lability of the seven-membered ring and the associated presence of conformers with similar energy. Thus, the VCD results do not conclude with a definite AC assignment to carbacephem **6**, while ECD does.

Derivatives of 3,4-benzo-5-oxacepham **7**–**9**, called oxabenzoxals here, contain two important fragments in their skeleton, i.e., oxacepham (oxygen cephalosporin analogs) and 2H-1,3-benzoxazine. The first represents one of the most clinically essential antibiotics known. The second, in turn, is helpful in medicine as anti-inflammatory, antifungal, antibacterial, anti-HIV, anticancer, anticonvulsant agents, as photochromic substances and photo fading-preventive materials in material science [51,52]. Their hybrid structure, consisting of two motives responsible for their diverse bioactivity, can most likely even bring therapeutic applications, making them interesting research objects for medicinal and synthetic chemists. The interest in these potentially potent pharmaceuticals is further encouraged because, thus far, literature reports associated with them are exceedingly rare in synthetic and spectroscopic fields [30,53,54,55]. Furthermore, the combination in one molecule of two strongly interfering chromophores, amide (β-lactam) and the 2H-1,3-benzoxazine subunit, partly absorbing at similar wavelengths, may considerably complicate the ECD spectra due to their mutual interaction.

**Figure 6:**
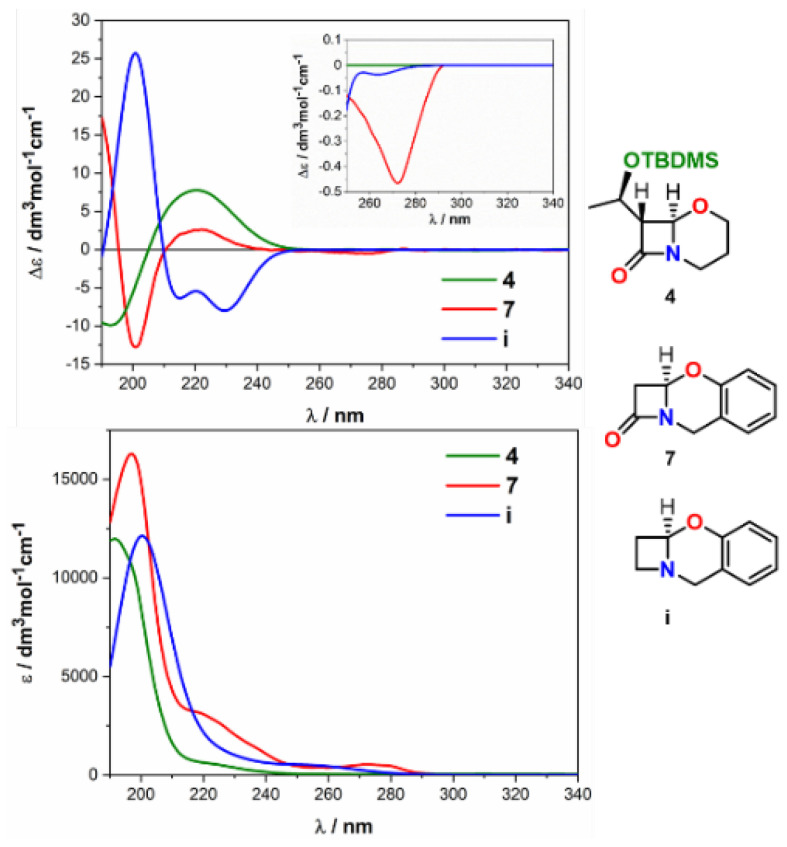
Comparison of experimental ECD and UV spectra of oxacepham **4** (green curve), oxabenzoxal **8** (red curve), and simulated spectrum for model compound ***i*** (blue curve). Calculated ECD spectrum for (***i***) was obtained with 0.44 eV, respectively, exponential half-width; red-shifted by 10 nm.

The thorough visual comparison of the ECD spectra of oxabenzoxals **7**–**9** and the shape of the curves allows one to declare that all electronic transitions within the 220 nm band, i.e., in the region of the amide *n*→π* transition, are complex due to contributions from both strongly committed chromophores, amide and 2H-1,3-benzoxazine. As shown in Figure 6, the spectra show good agreement in the high-energy region with their classical counterparts’ spectra represented here by oxacepham **4**. However, they differ in the longer-wavelength spectral region (between 250–300 nm) by indicating weak to very weak aromatic contributions at around 260 nm, exemplified in Figure 6 by compound **7** and its theoretical counterpart 2H-1,3-benzoxazine analog ***i*** without the carbonyl group, respectively.

**Figure 7:**
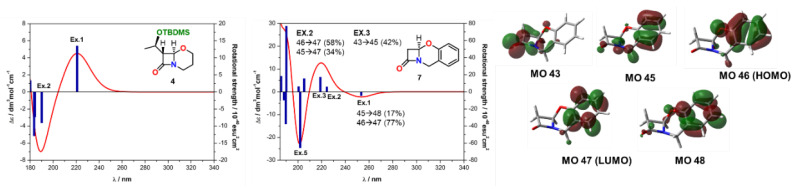
Simulated ECD curves for oxacepham **4** (**left**) and oxabenzoxal **7** (**middle**); visualized for ***7*** molecular orbitals involved in the three lowest energy transitions (**right**).

Detailed theoretical studies taking into consideration all aspects of the bichromophoric system’s electronic complexity, including the impact of absorbing substituents (if present), demonstrated that within the main ECD band at around 220 nm, at least two electronic transitions are observed, i.e., positive ones at 225 and 220 nm (Ex. 2 and Ex. 3) (Figure 7, right). This result, combined with the earlier analysis of the optical properties of oxacepham **4** (Figure 7, left) having only one single amide *n*→π* transition in this region, allows for assuming that the remaining excitation belongs to the 2H-1,3-benzoxazine part. However, the molecular orbital (MO) analysis of oxabenzoxal ***7*** showed that all electronic transitions within the band at ca. 220 nm (Ex. 2 and Ex. 3) are complex, and both amide and 2H-1,3-benzoxazine chromophores are strongly engaged.

**Figure 8:**
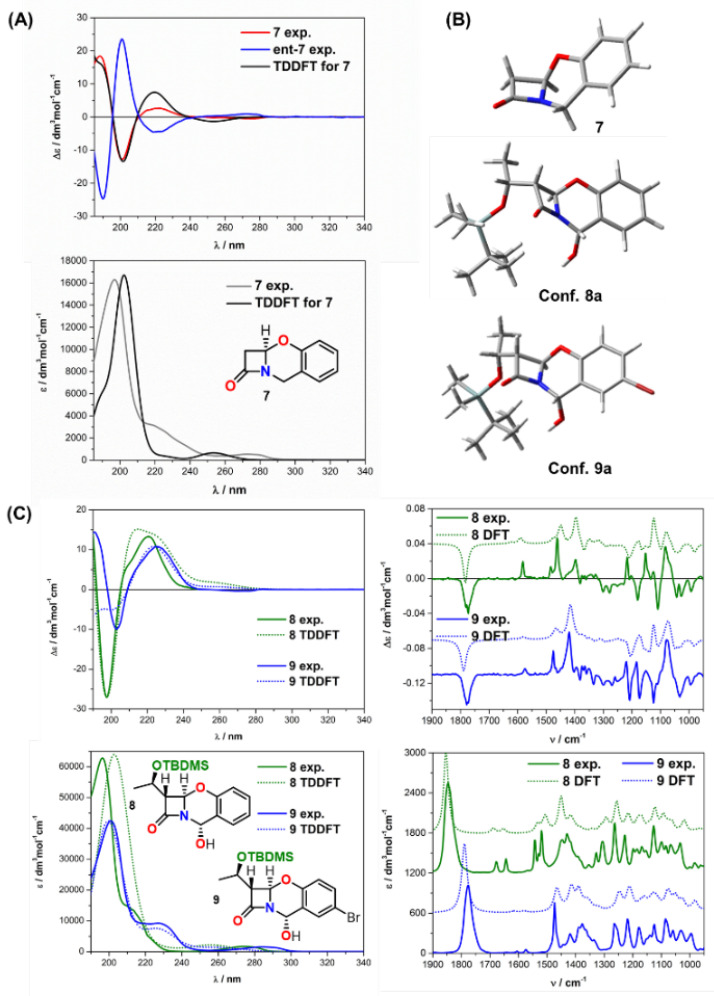
(**A**) Experimental and computed ECD/UV spectra of oxabenzoxal ***7*** compared to the ***ent*-7** experimental spectrum. (**B**) Lowest energy conformers of **7**–**9**; (**C**) Comparison of experimental ECD/VCD and UV/IR spectra of oxabenzoxals **8** and **9**. Calculated ECD spectrum for **7**, **8** and **9** were obtained with 0.26, 0.36, and 0.32 eV, respectively, exponential half-width; red-shifted by 14, 15, and 7 nm, respectively.

Nevertheless, despite the interaction between the chromophores, the amide electrons’ excitation predominates the transition centered at 220 nm. The shape of the MO orbital indicates the dominant contribution of the amide-type *n*→π* transition as well. The relatively high value of rotational strength equal to +15.5 × 10^−40^ esu^2^ cm^2^ for this transition determines the diagnostic for the helicity rule CE sign and is consistent with the rule. Thus, the oxabenzoxals understudy with the (6*R*) absolute configuration possesses a positive CE at around 220 nm. In contrast, this CE is negative for (6*S*) compounds, as the spectrum of ***ent*-7** proves (Figure 8). 

Consequently, the helicity rule’s effectiveness can also be concerned for this class of compounds at first glance. However, recognition of the universality of this rule for oxabenzoxals remains an open question at this stage. For example, if the rotational strengths of electronic transitions present within each ECD band are opposite in signs, the helicity rule’s applicability may become questionable. Therefore, supporting the experiment with theory to obtain accurate electronic transition assignments is particularly justified. In our case, the high compatibility of calculated and experimental ECD spectra with similarity factor values of 0.984 for **7**, 0.959 for **8**, and 0.989 for **9** confirm the assignment’s correctness.

A stereochemical assignment based on combined experimental-theoretical ECD analysis can be considered safe for the rigid oxabenzoxal **7** and its epimer ***ent*-7** without a substituent on the C7 carbon atom. However, for oxabenzoxals **8,9** with large flexible substituents at the C7 carbon atom and absorbing substituents in the aromatic ring, confirmation of the assignment based solely on ECD results seems to justify the use of an additional chiroptical method, including VCD, to strengthen its credibility. Analysis of the VCD spectra of oxabenzoxals **8** and **9**, however, did not bring satisfactory results. Comparing the experimental and theoretical VCD curves of compounds **8** and **9** results in a similarity factor of only 0.602 for **8**, 0.706 for **9**, and their enantiomers 0.069 and 0.065, respectively. Despite the almost ten times higher value of this coefficient for (6*R*) enantiomers, it is challenging to consider such an assignment fully confident.

The reasons for VCD failure for the β-lactam derivatives **5**, **6**, **8**, and **9** tested here are complex. Still, they can be associated, among others, with the structure of the compounds studied and computational constraints. In this context, the advantages of VCD consisting of the entire molecule analysis with its many vibration bands sensitive to the molecular structure’s details may become disadvantages of the method in the case of multi-chromophoric compounds and those with significant conformational freedom. This is because simulated spectra for compounds with many stereogenic centers reproduce experimental chiroptical spectra for only one diastereomer. Therefore, eliminating at least some diastereomers acting as possible contenders by other methods will provide the assignment with a higher degree of certainty [56]. 

In many cases, determining the proper diastereomer and establishing the exact Boltzmann distribution of conformers leads to fully resolving the studied stereochemical problem. Nonetheless, numerous well-populated conformers in a solution reduce the intensity of VCD bands and thus also spectra’s quality. It also imposes increased DFT calculations’ requirements to predict the proper estimation of the conformer population and the bands’ origin, some of which may be distorted or reduced by overlapping and suppressing VCD intensity by neighboring bands with opposite signs.

Further, the interpretation of VCD spectra is strongly dependent on the quality of quantum chemical simulations. The two main drawbacks of standard VCD calculations are their limitation to the harmonic approximation and the solvent effect that must be carefully considered. Generally, calculations are performed in harmonic approximation in which only fundamental transitions involving a single quantum of vibrational energy are allowed, and intensities of overtones and combination bands are predicted to be zero. However, this is not the case at higher frequencies. Therefore, attaching anharmonicity to the calculation increases the accuracy of calculated vibrational frequencies and, thus, the bands’ position [57]. The solvent effects, both implicit and explicit, can, in turn, help better understand the intermolecular interactions in the solution, ultimately leading to increased compatibility between calculated and measured VCD spectra [58,59,60].

**Figure 9:**
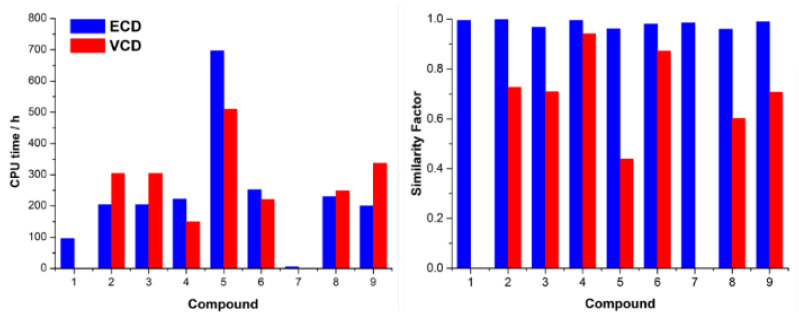
Comparison of CPU times for ECD and VCD simulations (**left**) of tested β-lactams in terms of their similarity factors (SFs) determined based on experimental spectra (**right**). Note: For **1** and **7**, VCD property is not probed.

A good balance between computational cost and accuracy is always required for calculations of chiroptical properties [61]. Thus, to judge the best-quality computational cost-to-time ratio, we collate CPU times and SFs in Figure 9 for all investigated compounds, separately for ECD and VCD simulations. It is shown that we achieved good accuracy for calculations carried out at the same level of theory using the same pool of conformers for most compounds. In particular, for ECD simulated spectra, SFs are very high (more than 0.95) with pretty similar CPU times (calculated for the same number of electronic transitions) for most investigated compounds.

For VCD, CPU times are substantially in line with ECD simulations; however, the analysis of SFs is more complex. Particularly for more rigid compounds such as **4** and **6**, SF values are higher, while those containing flexible and bulk side chains have SFs lower as, for example, for **5**, it equals only 0.439. In general, lower SF values in VCD are mainly because vibrations from the chiral side chain (one chiral center near the TBDMS group) overlap the β-lactam backbone vibrations in the fingerprint part of the spectrum. Thus, many conformers are generated in which bands cancel out after Boltzmann averaging. This is particularly evident in **8** and **9**, which have a rigid β-lactam skeleton with a fused aromatic ring and have a relatively low SF of 0.602 and 0.706, respectively.

**Figure 10:**
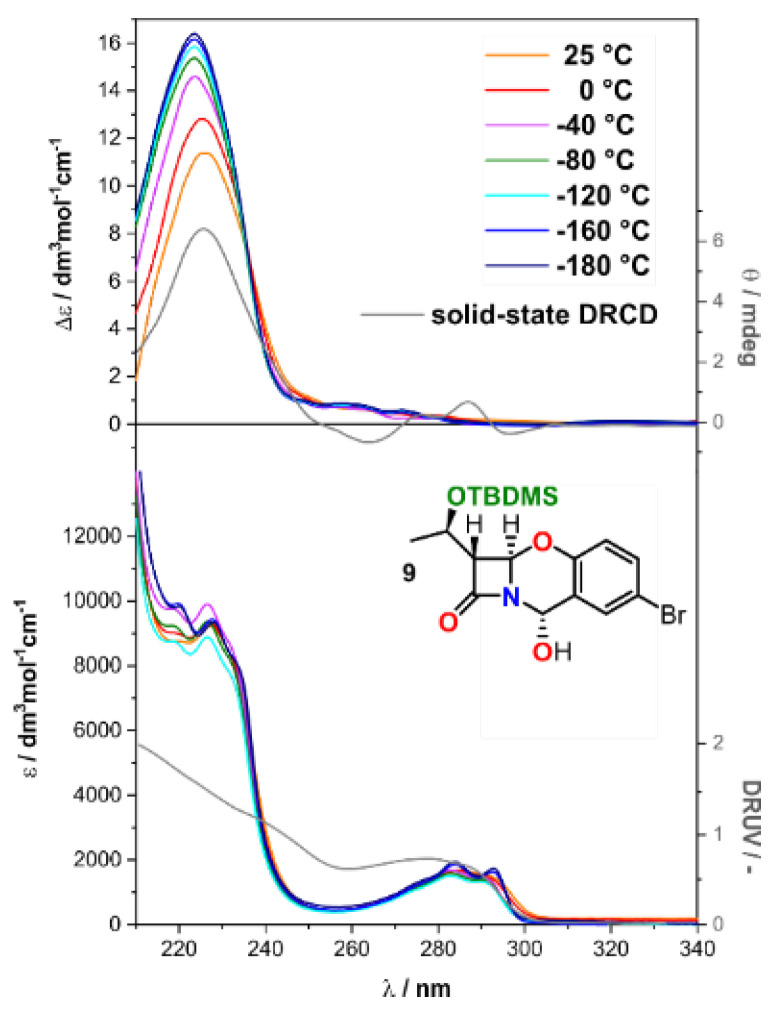
Variable temperature (VT) and diffused reflectance solid-state DRCD (**top**) and UV (**bottom**) spectra of oxabenzoxal **9** recorded in EPA (Et_2_O:iso-pentane:EtOH, 5:5:2, v/v). VT curves include correction for solvent contraction.

We ruled out the solvent effect as causative of inconsistent reproduction of experimental spectra by theory as its impact was accounted for using the PCM model in all calculations. Then, to provide evidence and find experimental confirmation of the effect of conformational flexibility on the chiral analysis result, we performed variable temperature (VT) measurements for compound **9** in the ultraviolet range as a test case. The measurements results performed in a polar solvent EPA (Et_2_O:iso-pentane:EtOH, 5:5:2, v/v) in temperatures ranging from +25 °C to −180 °C indicate pronounced conformational homogeneity of the rings comprising amide and 2H-1,3-benzoxazine chromophores as evidenced by only slight changes in the shape of the ECD curve along with the lowering of the temperature. These changes include a slight and uniform increase in the 225 nm band intensity and its hypsochromic shift of 4 nm with a decrease in temperature, strongly indicative of most molecules’ freezing in their preferred conformation (Figure 10).

The variable temperature measurements corroborated the skeleton’s relative conformational stability, although four conformers in the 2 kcal/mol energy window were found. These conformers mainly differ in the conformation of the C7 substituent and are essentially rotamers of the TBDMS group. However, due to this substituent distance from the rigid molecular scaffold containing chromophores, its rotamers do not adversely affect the simulated ECD spectra that show good agreement with the experiment.

To fully account for conformations of the flexible TBDMS sidechain, explore their real impact on the ECD spectrum, and check the effectiveness of the PCM model used in the calculations to estimate the solvent effect, we decided to measure the solid-state spectrum for the model compound **9**. For the first time, ECD spectroscopy for probing β-lactams in the solid state was successfully applied by us almost ten years ago and demonstrated to be a valuable tool in the chiral analysis of this class of compounds [46]. Therefore, further development in the context of advances in instrumentation and new measurement techniques is needed and continues to grow. We decided to use this developmental technique, namely the diffused reflectance CD technique (DRCD), to probe solid-state chirality. This methodology has great potential and is very attractive, especially in medicinal chemistry, because it minimizes sample preparation and avoids possible sample transformation problems under certain pellet preparation conditions for transmittance measurements. As a result, the recorded spectrum is practically free from artifacts associated with the traditional microcrystalline pellet measurements [62]. In our case, excellent agreement between the solid-state spectrum and the computed one, as well as the close similarity with ECD spectra recorded in the low temperature (Figure 10), indicates the presence of the same molecular species in both states and shows that the observed ECD is based solely on a molecular property. This also means that the solute–solvent interactions, which can result in conformational and vicinal effects, are negligible, thus confirming using the PCM solvent model in the calculations as suitable.

**Figure 11:**
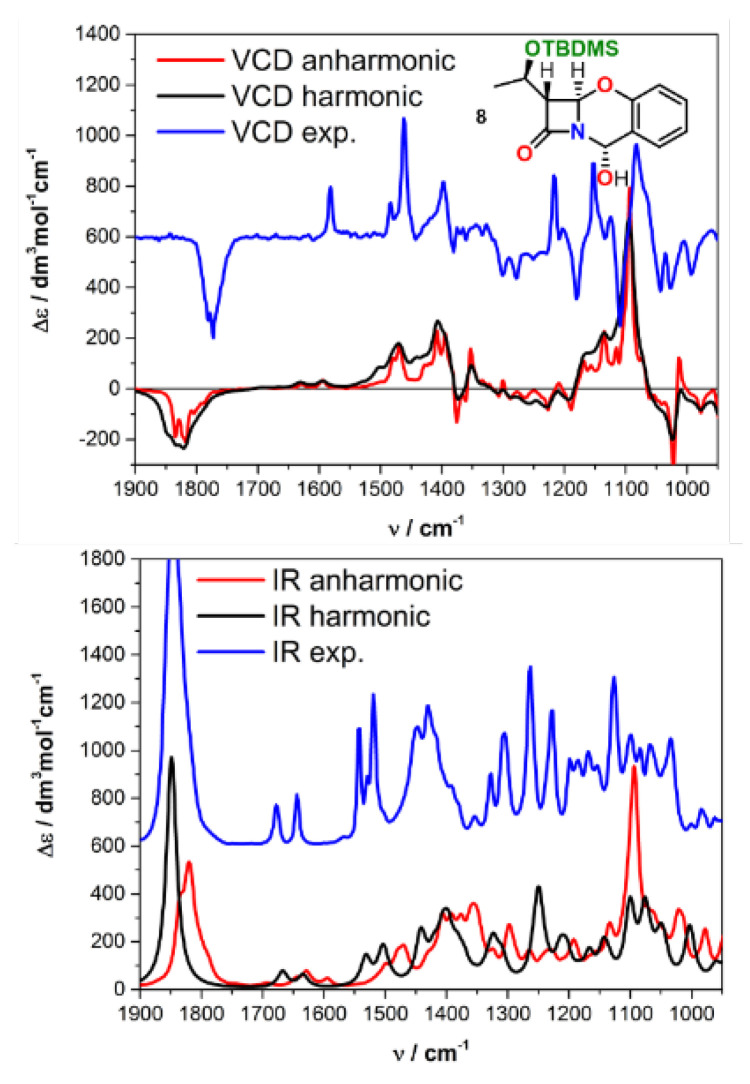
Comparison of simulated VCD (**top**) and IR (**bottom**) spectra of oxabenzoxal **8** with and without anharmonic approximation in calculations.

Obtaining fully reliable VCD spectra results in the case of (*R*)-4-X-2-azetidinone derivatives (X = F, Cl, Br) necessitated conducting calculations at a fully anharmonic level [58]. Since our compounds are, in fact, counterparts to those described, we considered it appropriate to include anharmonicity in our vibrational calculations, expecting better agreement with the experiment for those incompatible. For these calculations performed at the B3LYP/6–31G(d)/PCM(CH_3_CN) level of theory, we chose compound **8** using its previously selected three-conformer pool, mainly differing in the TBDMS group orientation. Oxabenzoxal **8** was selected as a model due to its lower similarity factor (SF) than **9** in harmonic approximation, equal to 0.602 and 0.706, respectively (Figure 9). As shown in Figure 11, the introduction of the anharmonic term into the calculations did not significantly improve the theoretical spectrum quality as indicated by SF of 0.604. At the same time, Δε is 0.520 for an anharmonic calculation vs. 0.533 for harmonic one, where Δε is the difference of SFs for enantiomers. This result means an almost complete convergence with the results calculated based on the harmonic approximation. In contrast, it should be strongly emphasized that the CPU time consumption has increased 100 times regarding harmonic calculations. With no improvement in compliance with the experiment, especially in the most problematic sub-region 1250–1125 cm^−1^, such a result is discouraging while pointing clearly to other reasons for the observed discrepancy.

**Figure 12:**
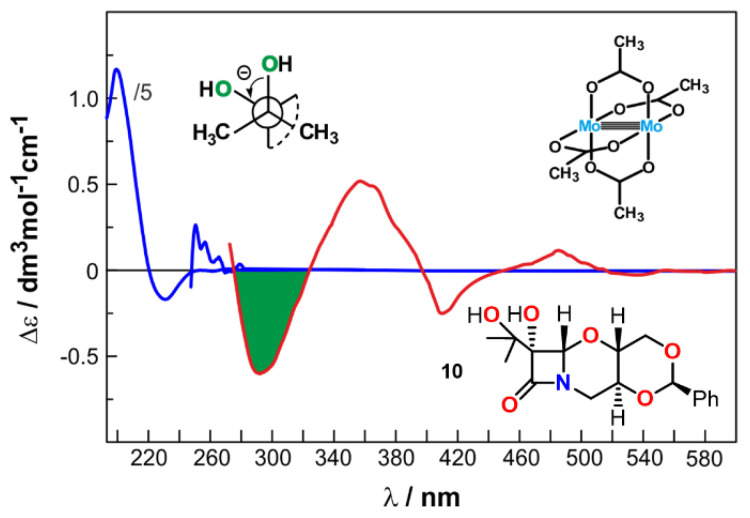
ECD spectra of tricycloxacepham **10** (blue line) and of its in situ formed complex with dimolybdenum tetracetate (red line). The inset shows predicted negative sign of the O-C-C-O *vic*-diol torsion angle (left-top), the formulas of dimolybdenum tetraacetate (right-top), and tricycloxacepham **10** (right-bottom). The ECD curve of **10** (blue line) in the range of 190–250 nm is multiplied five times.

The absolute configuration at C6 carbon atom of tricyclic oxacepham derivative **10**, named according to IUPAC (2*R*,4a*R*,5a*S*,6*S*,9a*S*)-6-hydroxy-6-(10-hydroxy-10-methyl-ethyl)-2-phenyl-1,3,5-trioxa-8-aza-cyclobuta[b]decalin-7-one, was previously established by synthetic route, NMR and CD spectroscopies as (6*S*) [34,35]. The negative CE sign at 230 nm visible in its ECD spectrum corresponds to (6*S*) AC, correlating well with the helicity rule as well as the synthetic route and NMR data (Figure 12). However, an open matter remained the AC at the C7 carbon atom. Considerable mobility of the substituents on this carbon, most likely resulting in a diversity of near-energy conformers and rotamers, hindered the computing-supported CD analysis, including VCD as we have explicated just above and very recently by Batista et al. [63]. On the other hand, transforming the *tert/tert* 1,2-diol subunit present in **10** into an ECD-active cottonogenic derivative is not straightforward. Another solution to this problem offers the so-called in situ dimolybdenum method [23,64,65,66]. This simple, fast, and yet reliable method is highly effective for both flexible and sterically demanding *vic*-diols, including *tert/tert vic*-ones. Despite the empirical nature of the method, its great advantage is a transformation of labile systems into almost rigid and, above all, conformationally defined derivatives. Ligating the flexible diol molecule to the Mo2-core causes a significant reduction in the diol’s internal conformational mobility due to the stock complex’s steric requirements [64,65]. As a result, in most cases, the molecule appears to exist as a single conformer with an antiperiplanar orientation of both O-C-C-R units only. This is very reasonable as only in this conformation are the bulky R groups directed away from the rest of the complex and avoid close interaction with the remaining acetate ligands of the stock complex. Quantum-mechanical computation provides additional evidence to support such a preferred structure [67]. This fixed conformation, in turn, enabled determining the absolute configuration of the 1,2-diol moiety alone from its ECD spectrum recorded in the presence of dimolybdenum tetraacetate acting as an auxiliary chromophore [35]. The AC is assigned according to the helicity rule, binding the sign of the torsion angle of the diol group (O-C-C-O) with the CE signs sequence arising in the 300–400 nm range, namely a positive torsion angle correlates with positive CEs at ca. 300 and 400 nm and a negative one ca. 350 nm and vice versa, i.e., a negative torsion angle correlates with negative CEs at ca. 300 and 400 nm and a positive one ca. 350 nm.

Consequently, a negative sign of CEs at 295 and 415 nm as well a positive one centered at 360 nm in the spectrum of **10** corresponds to a negative sign of the O-C-C-O torsion angle. Based on this result, supported by calculations confirming the O-C-C-O torsional angle value of −53.1 in the lowest energy conformer, we could confidently assign (*R*) AC to carbon C7. This assignment is even more justified since excitations of both other chromophores present in the molecule of **10**, the β-lactam at 230 nm and phenyl with its fine structure in the 255–275 nm range, are outside the Mo-diol complex range and thus do not interfere with its excitations. Once again, the in situ dimolybdenum method has been proven effective, as has been the case in many other examples previously described in the literature.

## 3. Materials and Methods

### 3.1. General Information

Source of Investigated Compounds. Synthesis and full structural characterizations of compounds **1** [48], **4** [31,68], **5** [49], **6** [46], **7** [53,54], ***ent*-7** [53,54], **8** [30], **9** [30] and **10** [34], have already been published. Synthetic details and spectroscopic characterization of compounds **2** and **3** are available in Supplementary Materials. ECD/UV spectra were recorded using a J-815 spectrometer (Jasco, Tokyo, Japan) at room temperature in spectroscopic-grade solvents. Solutions were measured in quartz cells with path lengths of 2, 1, 0.5, and 0.1 cm. All spectra were recorded using a scanning speed of 100 nm min^−1^, a step size of 0.2 nm, a bandwidth of 1 nm, a response time of 0.5 s, and an accumulation of 4 scans. The spectra were background-corrected using the respective solvent spectra recorded under the same conditions. For the ECD measurements with an auxiliary chromophore, the chiral ligand 10 (∼3 × 10^−3^ M) was dissolved in a stock solution of Mo_2_(O_2_CCH_3_)_4_ (∼2 × 10^−3^ M) in DMSO, so that the final molar ratio of ligand used to the stock complex used was ∼1.5:1. VT-ECD measurements of 9 were carried out using an Optistat optical spectroscopy cryostat (Oxford Instruments, Abingdon, UK) attached to the sample chamber of ECD instrument, in the temperature range of +25 to −180 °C using EPA (Et_2_O:*iso*-pentane:EtOH, 5:5:2, v/v) as solvent. Baseline correction was performed by subtracting the spectrum of a reference solvent obtained under the same conditions; all VT-ECD spectra were normalized using a concentration at the proper temperature. The solid-state Diffused Reflectance CD (DRCD) spectrum was obtained by mixing 2.5% of **9** with the KCl powder, and then it was placed in a dedicated holder. All DRCD spectra were carried out with the integrating sphere compartment (model DRCD-466 L) coated with BaSO_4_ dedicated to J-815 using 100 nm/min scanning speed, a step size of 0.2 nm, a bandwidth of 4 nm, a response time of 0.5 s, and an accumulation of 5 scans. Eight independent rotations (every 45 deg) were performed in each case to obtain a high-quality spectrum. Finally, all measured spectra were averaged. VCD/IR spectra were collected using a Chiral*IR*-2X VCD spectrometer from BioTools Inc. (Jupiter, FL, USA) at a resolution of 4 cm^−1^ in the range of 2000–850 cm^−1^ in spectroscopic-grade CH_3_CN or CHCl_3_ for 6 h. The spectrometer was equipped with dual sources and dual ZnSe photoelastic modulators (PEMs) optimized at 1400 cm^−1^. A solution was measured in a BaF_2_ cell with a path length of 100 μm assembled in a rotating holder. Baseline correction was achieved by subtracting the spectrum of solvent recorded under the same conditions.

### 3.2. Computational Section

Conformational searches were run with CONFLEX 7 (Tokyo, Japan) [69,70,71] using default grids and convergence criteria (MMFF94s force fields). All structures thus found were optimized with DFT at the ωB97X-D/6–311+G(d,p) level in the PCM solvent model for CH_3_CN or CHCl3. Further frequency calculations were run at the same level of approximation. All relevant structures had zero imaginary frequencies. DFT and TDDFT calculations were run with the Gaussian16 suite (Wallingford, CT, USA) [72], using default grids and convergence criteria. TDDFT calculations were run on ωB97X-D/6–311+G(d,p). Other functionals (including B3LYP and CAM-B3LYP) were also tested; the results are in line with ωB97X-D. The final ECD/UV spectra were averaged according to the Boltzmann distribution at 293 K using the populations estimated from internal and free energies. All spectra were averaged and plotted using SpecDis (Berlin, Germany) [37,73]. An optimum Gaussian band-shape and UV-correction were selected according to the similarity analysis with the experimental data performed using SpecDis. VCD/IR simulations were carried out for the same pool of conformers as the ECD/UV spectra, using a ωB97X-D functional and 6–311+G(d,p) basis set with a PCM solvent model for CH_3_CN or CHCl_3_. In each case, the Boltzmann-averaged spectrum was converted to Lorentzian bands with a half-width and scaling factor indicated by SpecDis to obtain the best similarity factor with the experimental data. Additionally, for **8**, anharmonic VCD/IR spectra were calculated using the B3LYP functional and 6–31G(d) basis set with the PCM solvent model for CHCl_3_.

## 4. Conclusions

It is commonly believed that in pharmaceutically relevant compounds, one of the most critical tasks is to determine their absolute configuration reliably. With this in mind, in this study, we conducted a systematic circular dichroic study of structurally different β-lactams to demonstrate the importance of chiroptical methods in practical stereochemical analysis and their effectiveness in determining the absolute configuration. In line with generally accepted principles aimed at increasing the results’ reliability, we verified the stereochemical conclusions using as many available methods as possible, including a combined experimental and theoretical analysis. In some cases, however, limitations of particular techniques, e.g., computational time costs, prevent extended comprehensive analysis. In such cases, an in-depth CD study focused on individual classes of compounds may be justified by indicating the most appropriate choice technique for them.

Here, we have shown that ECD spectroscopy is the first-choice method for determining the absolute configuration of various β-lactams and that VCD performs a rather complementary role in most cases. This complementary role of VCD has mainly been observed when a relatively simple compound shows a complex VCD spectrum due to large flexible substitutes, e.g., TBDMS, especially in combination with additional stereogenic center(s). Despite the relatively high rigidity of the molecular skeleton, a large number of rotamers within the TBDMS complicates the VCD spectrum so much that obtaining a good match of theory with experiment is significantly difficult, if not impossible. In contrast, this substituent effect is negligible in the ECD spectrum due to its considerable distance from the chromophoric units and the location of TMDMS rotamers’ contributions only in the short-wavelength part of the spectrum.

Upon trying to determine the causes of the observed discrepancies between measured and calculated VCD spectra, we found that including anharmonicity in the calculations does not improve this compatibility while significantly increasing, even 100 times, the CPU time. The calculations clearly showed this for compound **8**, for which calculations were conducted in both harmonic and anharmonic approximations. Measurements at variable temperatures and the solid state, in turn, allowed us to demonstrate a relatively high rigidity of the molecular skeletons of the studied β-lactams, thus excluding the influence of their possible flexibility on the obtained results. Moreover, present studies proved the effectiveness of the PCM solvent model used in the calculations. Consequently, the only reason for the observed discrepancies between experimental and simulated VCD spectra seems to be the presence of a spatially large and labile substituent on the C7 carbon together with its numerous rotamers, which practically prevents the correct simulation of VCD spectra.

We also showed the usefulness of conventional methods with their helicity and sectors rules, the utility of which cannot be overestimated in some cases. We have also indicated new measurement techniques currently being developed, such as DRCD for the solid state. Although Castiglioni already introduced this methodology in 2000 [62], its use in the field of biologically active substances has been very limited. Due to the non-invasive nature of the measurement, this technique gives a new lease of life in the context of its use for differentiation polymorphs of chiral APIs. Their development, combined with the continuous progress in computational methods, leads to the extension of the available chiroptical methods, thus increasing the scope of their effectiveness to new areas. One example that needs clarification is the limited effectiveness of VCD in the analysis of some of the β-lactam systems presented in this article.

To summarize, extending the collected information should increase the credibility of the assignments made, ultimately strengthening the position of chiral analysis in stereochemical tests.

## Figures and Tables

**Table 1:** Relative energies and populations at 298 K (≥1%) based on DFT-optimized conformers of **1**–**9** within 3 kcal/mol calculated at ωB97X-D/6–311+G(d,p) level with PCM solvent model for CH_3_CN. The results of a similarity analysis (SF) are obtained using Boltzmann-averaged simulated spectrum and experimental one; for each compound, CPU computational efficiency is also provided, separately for DFT optimizations, and ECD/VCD simulations.

Comp.	Conf.	ΔE/ kcal mol^−1^	Pop./%	SF for ECD	SF for VCD	CPU Time/h		
						DFT opt.	ECD	VCD
**1**	**1-up a**	0.00	63.31	0.991	n/a	111	96	132
	**1-up b**	0.60	23.10					
	**1-up c**	0.91	13.59					
	**1-down a**	0.00	62.17	0.000	n/a	127	96	134
	**1-down b**	0.51	26.26					
	**1-down c**	1.00	11.57					
**2**	**2a**	0.00	40.82	0.998	0.726	261	204	304
	**2b**	0.27	25.98					
	**2c**	0.48	18.03					
	**2d**	0.97	7.94					
	**2e**	1.14	5.97					
	**2f**	2.06	1.26					
**3**	**3a**	0.00	39.07	0.967	0.708	282	204	304
	**3b**	0.09	33.46					
	**3c**	0.68	12.42					
	**3d**	1.13	5.75					
	**3e**	1.25	4.72					
	**3f**	1.27	4.57					
**4**	**4a**	0.00	54.87	0.995	0.941	201	222	149
	**4b**	0.61	19.62					
	**4c**	0.93	11.35					
	**4d**	1.32	5.92					
	**4e**	1.52	4.20					
	**4f**	1.54	4.04					
**5**	**5a**	0.00	72.12	0.961	0.439	533	696	510
	**5b**	0.86	16.89					
	**5c**	1.47	6.04					
	**5d**	2.06	2.21					
	**5e**	2.29	1.52					
	**5f**	2.41	1.23					
**6**	**6a**	0.00	60.49	0.980	0.871	352	252	221
	**6b**	0.84	14.63					
	**6c**	1.03	10.68					
	**6d**	1.30	6.70					
	**6e**	1.36	6.07					
	**6f**	2.22	1.43					
**7**		0.00	100	0.984	n/a	3	5	2
**8**	**8a**	0.00	68.05	0.959	0.602	228	230	248
	**8b**	0.65	22.82					
	**8c**	1.67	4.05					
	**8d**	1.92	2.65					
	**8e**	1.97	2.43					
**9**	**9a**	0.00	58.10	0.989	0.706	259	200	337
	**9b**	0.32	34.08					
	**9c**	1.52	4.46					
	**9d**	1.69	3.36					

n/a = not applied.

## Data Availability

Not applicable.

## Supplementary Materials

The following supporting information can be downloaded at: https://www.mdpi.com/article/10.3390/ijms23010273/s1.

## Abbreviations

3D	three-dimensional structure
AC	absolute configuration
CD	circular dichroism
DFT	density functional theory
DRCD	diffused reflectance CD
ECD	electronic circular dichroism
PCM	polarizable continuum model
PES	potential energy surface
QC	quantum chemistry
SF	similarity factor
TBDMS	*tert*-Butyldimethylsilyl
VCD	vibrational circular dichroism
