# Supplementary material for: A Holistic Approach to Determining Stereochemistry of Potential Pharmaceuticals by Circular Dichroism with β-Lactams as Test Cases
Source: Int J Mol Sci. 2021 Dec 27;23(1):273. doi: 10.3390/ijms23010273 (PMC8745598; doi:10.3390/ijms23010273)
Supplement: Supplementary file 1 [file ijms-23-00273-s001.zip › ijms-1511185-supplementary.pdf]

Supplementary Materials

1. **General Techniques** **pag. S2**
2. **Synthesis of model β-lactams 2 and 3** **pag. S2**
3. **References** **pag. S4**
4. **NMR and MS spectra of Comp. 2** **pag. S6**
5. **NMR and MS spectra of Comp. 3** **pag. S13**
6. **Coordinates of calculated structures** **pag. S23**

1. General Techniques

All solvents were dried and distilled before use. All reactions were monitored by thin-layer chromatography using aluminum-backed silica gel plates 60 F_{254}; visualization was accomplished with UV light and/or staining with 50% H_2SO_4. Standard flash chromatography procedures were followed using silica gel with particle size 40-63 µm. FT-IR spectra were recorded on an FT-IR spectrophotometer for films. ^{1}H NMR spectra were recorded at 500 or 600 MHz and ^{13}C NMR at 125 and 150 MHz using $CDCl_3$ or C_6D_6 as solvents and TMS as internal standard and are reported as δ values (ppm) relative to residual $CHCl_3$ signal δ H (7.26 ppm) and $CDCl_3$ δ C (77.16 ppm), respectively. Mass spectra were obtained at 70 eV. Electrospray ionization (ESI) mass spectrometry (MS) experiments were performed on a mass spectrometer under normal conditions. PFK solution was used as a calibrant for HRMS measurements.

2. Synthesis of model β-lactams 2 and 3

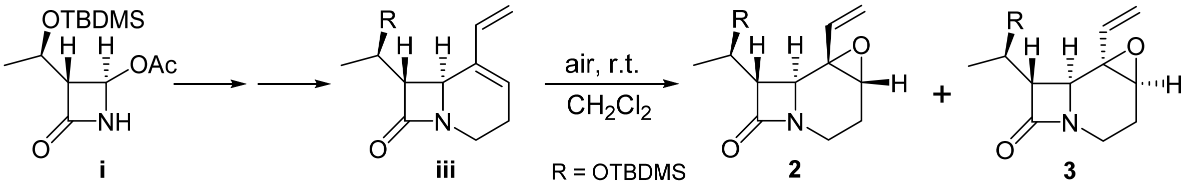

The bicyclic diene **iii**, quite widely used as a convenient and promising substrate for further transformations aimed at obtaining new antibiotics with an enhanced or novel biological activity profile, was synthesized in a few-step synthesis according to the literature procedure.[1-3] Spectroscopic characteristics obtained in a good yield of brown-yellow oil diene **iii** were fully consistent with the literature data.[1]

Under aerobic conditions at room temperature, the diene **iii** underwent a spontaneous epoxidation to yield a mixture of two epoxides in a ratio of 17:1. The yield

of the self-epoxidation reaction accompanied by the decomposition of product **iii** was 25% after three days at room temperature. Thus, the reactivity of **iii** toward atmospheric oxygen is much smaller than its five-membered counterpart with the formation of 4-vinyl-1-azabicyclo[3.2.0]hept-3-en-7-one with a 70% yield.[4] This result is in line with the impact of internal skeletal strain on the epoxidation reaction rate descending with increasing ring size, *i.e.*, 4,5 *vs* 4,6 in this case.[5] As checked in independent experiments, autoxidation of **iii** can be inhibited by a rigorous exclusion of oxygen from the reaction mixture and by keeping it at a low temperature (refrigerator).

Adding an oxygen atom to the starting diene molecule confirmed the MS spectrum by increasing the molecular mass by 16 in **2** and **3**. Moreover, in the ^{1}H and ^{13}C NMR spectra of both epoxides, only the resonances of vinyl protons at C5 carbon atom were visible, as well as lack of resonances originating from the diene double bond inside the six-membered ring, thus giving additional evidence for diene **iii** spontaneous epoxidation.

Oily main product **2**: ^{1}H NMR (600 MHz, $CDCl_3$): δ 0.03 (s, 3H), 0.04 (s, 3H), 0.85 (s, 9H), 1.25 (d, *J*=6.2 Hz, 3H), 2.01 (dt, *J*=15.0, 3.3 Hz, 1H), 2.12 (dddd, *J*=15.0, 11.8, 3.3 Hz, 1H), 2.83 (ddd, *J*=16.2, 11.8, 4.4 Hz, 1H), 3.14 (dd, *J*=6.2, 2.4 Hz, 1H), 3.17 (t, *J*=1.2 Hz, 1H), 3.57 (dd, *J*=13.8, 6.3 Hz, 1H), 3.76 (bs, 1H), 4.14 (dq, *J*=6.2 Hz, 1H), 5.23 (dd, *J*=10.8, 1.3 Hz, 1H), 5.48 (dd, *J*=17.1, 1.3 Hz, 1H), 5.99 (dd, *J*=17.1, 10.8 Hz, 1H). ^{13}C NMR (125 MHz, $CDCl_3$): δ -4.84, -4.36, 17.91, 22.87, 24.95, 25.69 (3C), 33.73, 51.36, 60.21, 61.67, 61.94, 66.13, 118.07, 133.42, 169.64. IR ($CHCl_3$): 1744, 1642, 1472, 1256 cm^{-1}. ESI-MS m/z

[(M+H)$^+$] 324.1996 *(calculated for* $C_{17}H_{29}NO_3Si$ = 324.1995*).* ESI-HRMS: m/z [(M+Na)$^+$] 346.1812 (calculated for $C_{17}H_{29}NNaO_3Si$ = 346.1815).

Oily minor product **3**: ^{1}H NMR (600 MHz, $CDCl_3$): δ 0.06 (s, 3H), 0.08 (s, 3H), 0.85 (s, 9H), 1.19 (d, *J*=6.3 Hz, 3H), 1.99 (dddd, *J*=8.58, 5.72, 3.22 Hz, 1H), 2.16 (dddd, *J*=16.0, 8.7, 6.32, 2.0 Hz, 1H), 2.77 (ddd, *J*=13.5, 7.9, 6.3 Hz, 1H), 3.01 (dd, *J*=4.5, 1.9 Hz, 1H), 3.14 (dd, *J*=3.0, 2.1 Hz, 1H), 3.62 (ddd, *J*=13.4, 8.9, 5.7 Hz, 1H), 3.89 (d, *J*=1.9 Hz, 1H), 4.19 (dq, *J*=10.8, 6.2, 4.7, 1.7 Hz, 1H), 5.28 (dd, *J*=10.8, 1.1 Hz, 1H), 5.48 (dd, *J*=17.3, 1.1 Hz, 1H). 5.88 (dd, *J*=17.3, 1.1 Hz, 1H). ^{13}C NMR (125 MHz, $CDCl_3$): δ -4.91, -4.37, 17.95, 20.13, 23.07, 25.71 (3C), 32.32, 49.30, 57.75, 59.86, 62.37, 64.83, 117.97, 133.97, 168.47. IR ($CHCl_3$): 1746, 1641, 1472, 1258 cm^{-1}. ESI-HRMS: m/z [(M+H)$^+$] 324.1996 (calculated for $C_{17}H_{29}NO_3Si$ = 324.1995).

3. References

1. Desroy, N.; Robert-Peillard, F.; Toueg, J.; Hénaut, C.; Duboc, R.; Rager, M.-N.; Savignac, M.; Genet, J.-P., Synthesis of New Polycyclic β-Lactams via One-Pot Enyne Metathesis and Diels-Alder Reactions, *Synthesis,* **2004,** *16*, 2665-2672.

2. Kumar, Y.; Singh, P.; Bhargava, G., Recent developments in the synthesis of condensed β-lactams, *RSC Adv.* **2016,** *6(101)*, 99220-99250.

3. Desroy, N.; Robert-Peillard, F.; Toueg, J.; Duboc, R.; Hénaut, C.; Rager, M.-N.; Savignac, M.; Genêt, J.-P., An Efficient Route to 4/5/6 Polycyclic β-Lactams, *Eur. J. Org. Chem.,* **2004,** *23*, 4840-4849.

4. Polavarapu, P. L.; Frelek, J.; Woźnica, M., Determination of the absolute configurations using electronic and vibrational circular dichroism measurements and quantum chemical calculations, *Tetrahedron: Asymmetry,* **2011**, *22(18)*, 1720-1724.

5. Bartlett, P. D.; Banavali, R., Spontaneous oxygenation of cyclic olefins. Effects of strain, *J. Org. Chem.*, **1991**, *56(21)*, 6043-6050.

4. NMR and MS spectra of Comp. 2

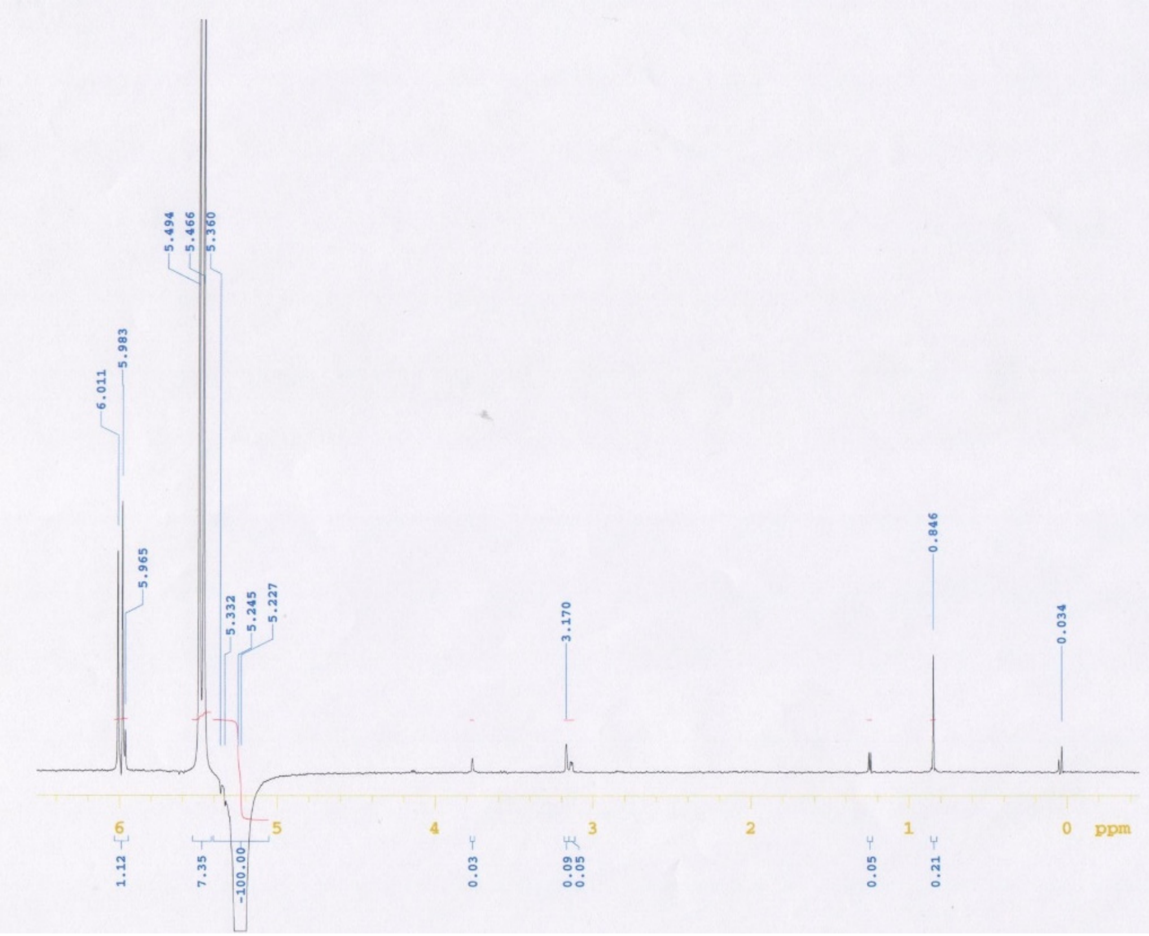

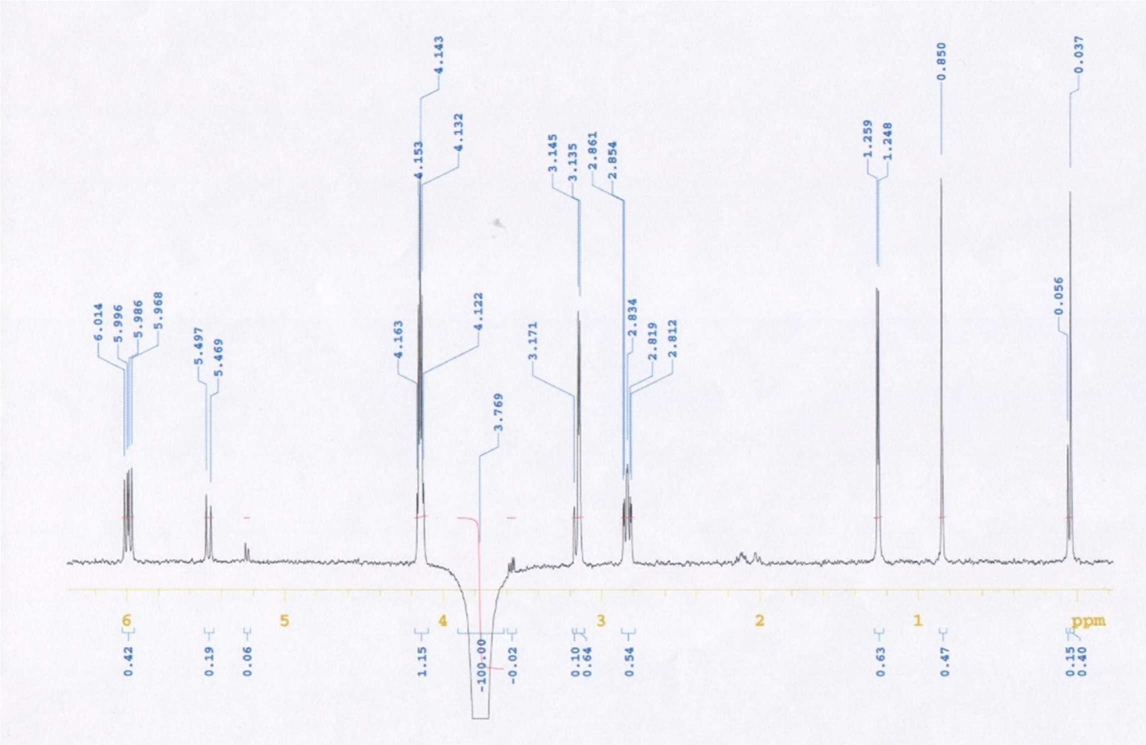
6.014
5.996
5.986
5.968
5.497
5.469
4.163
4.153
4.143
4.132
4.122
3.769
3.171
3.145
3.135
2.861
2.854
2.834
2.819
2.812
1.259
1.248
0.850
0.056
0.037
6
5
4
3
2
1
ppm
0.42
0.19
0.06
1.15
-100.00
-0.02
0.10
0.64
0.54
0.63
0.47
0.15
0.40

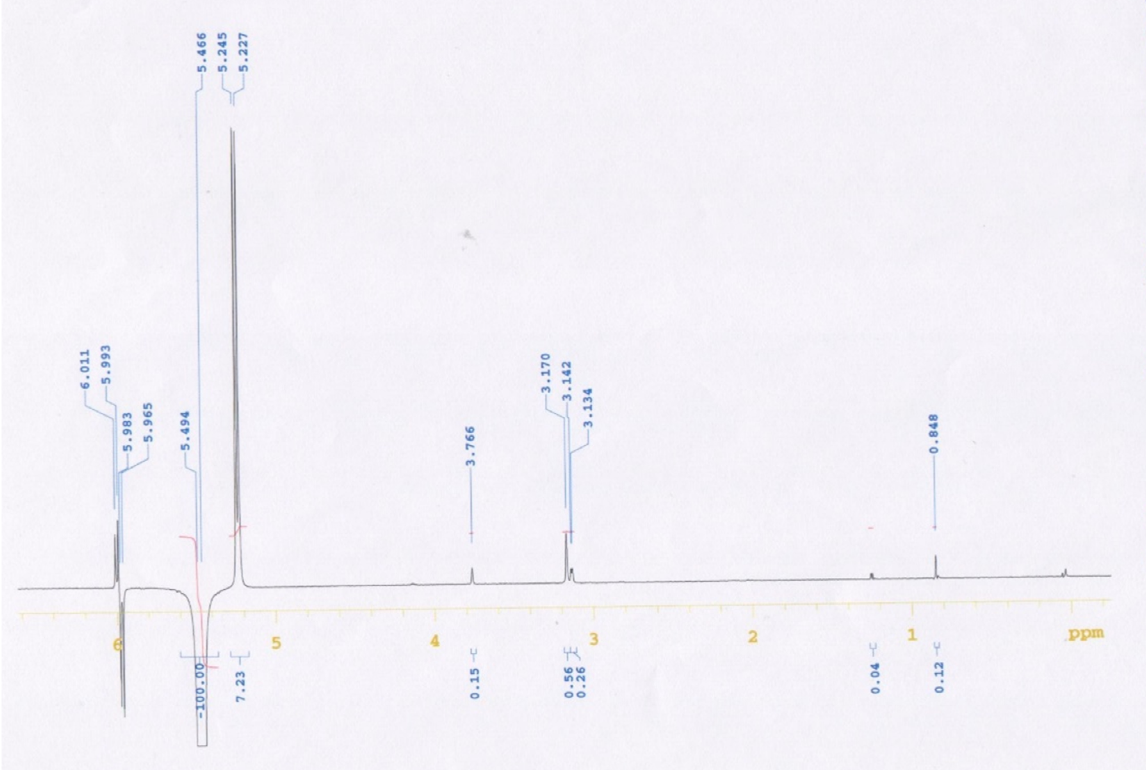
6.011
5.993
5.983
5.965
5.494
5.466
5.245
5.227
3.766
3.170
3.142
3.134
0.848
6
5
4
3
2
1
ppm
-100.00
7.23
0.15
0.56
0.26
0.04
0.12

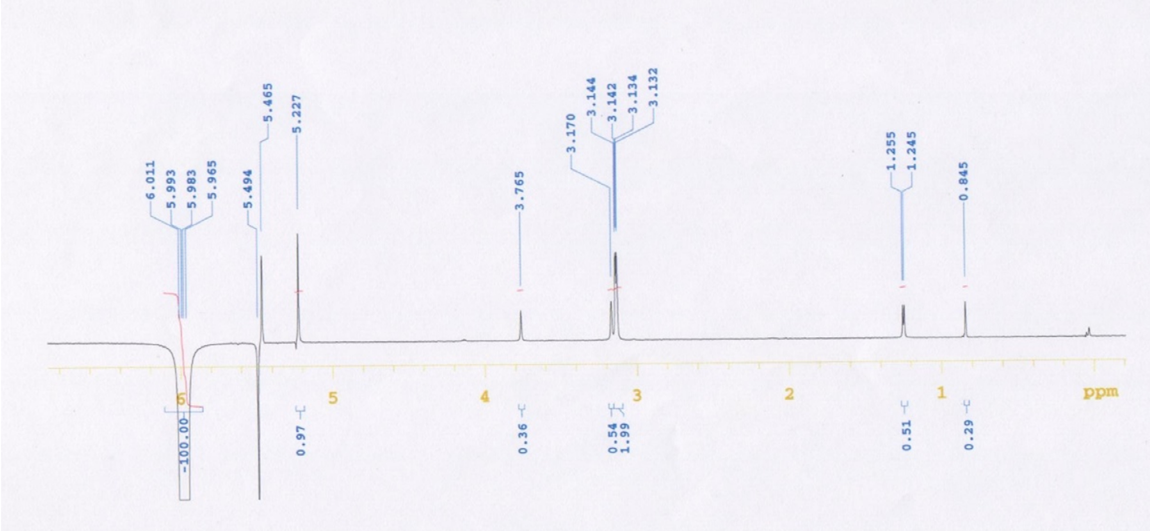
6.011
5.993
5.983
5.965
5.494
5.465
5.227
3.765
3.170
3.144
3.142
3.134
3.132
1.255
1.245
0.845
ppm
-100.00
0.97
0.36
0.54
1.99
0.51
0.29

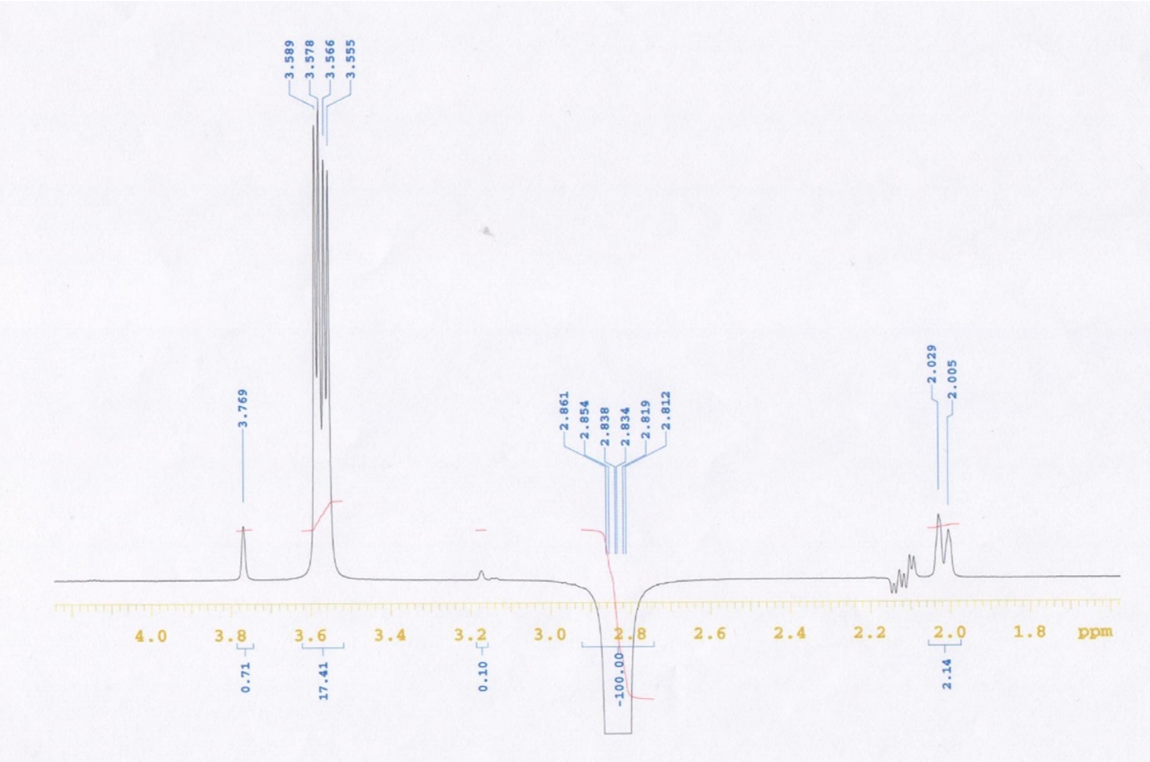
3.769
3.589
3.578
3.566
3.555
2.861
2.854
2.838
2.834
2.819
2.812
2.029
2.005
4.0
3.8
3.6
3.4
3.2
3.0
2.8
2.6
2.4
2.2
2.0
1.8
ppm
0.71
17.41
0.10
-100.00
2.14

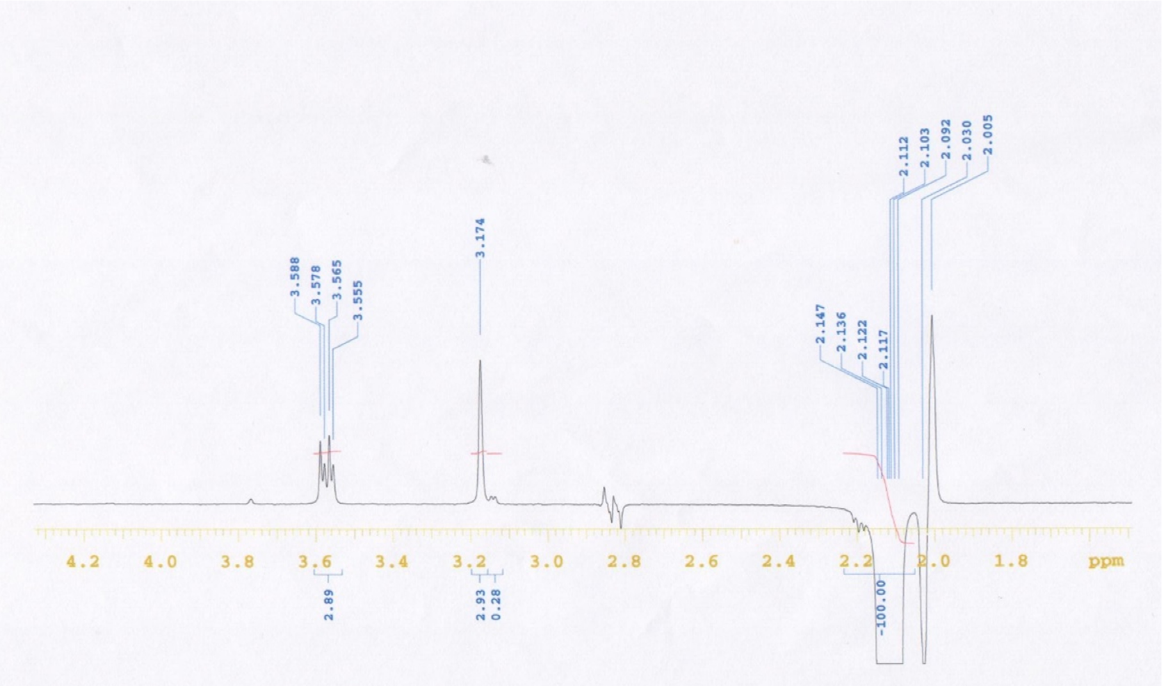

3.588
3.578
3.565
3.555
3.174
2.147
2.136
2.122
2.117
2.112
2.103
2.092
2.030
2.005
4.2
4.0
3.8
3.6
3.4
3.2
3.0
2.8
2.6
2.4
2.2
2.0
1.8
ppm
2.89
2.93
0.28
-100.00

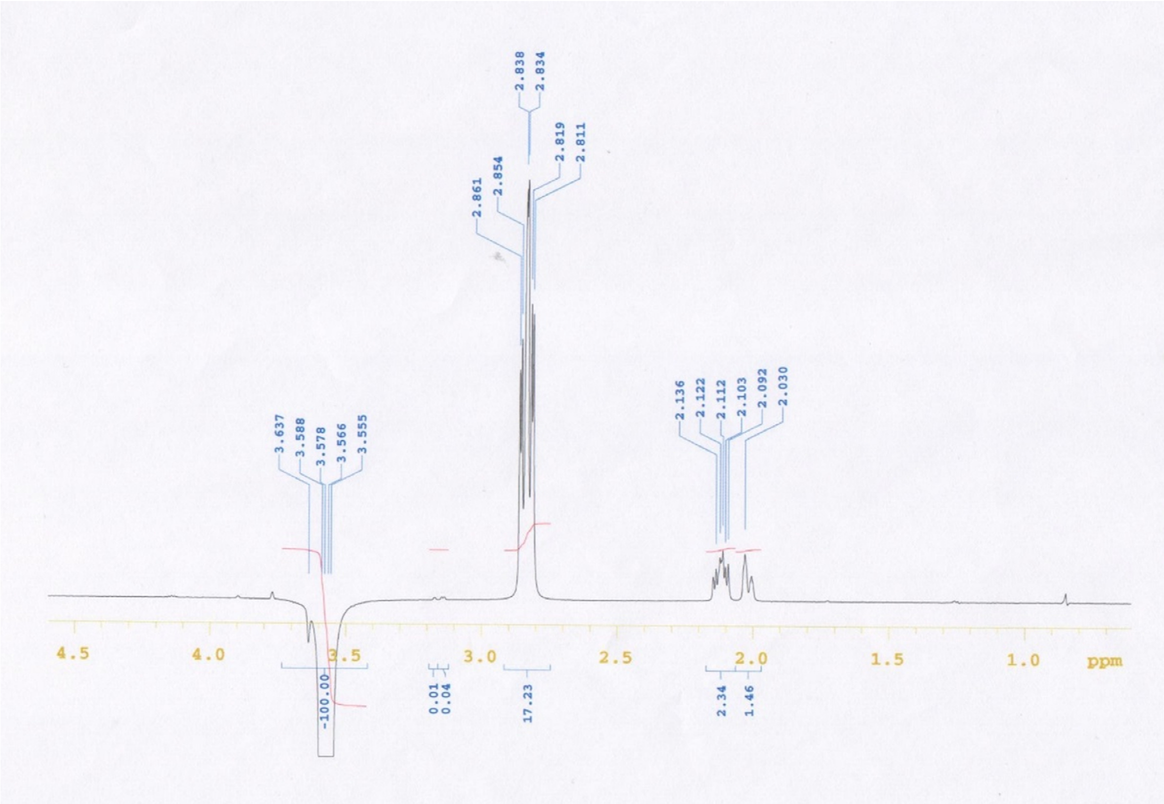

3.637
3.588
3.578
3.566
3.555
2.861
2.854
2.838
2.834
2.819
2.811
2.136
2.122
2.112
2.103
2.092
2.030
4.5
4.0
3.5
3.0
2.5
2.0
1.5
1.0
ppm
-100.00
0.01
0.04
17.23
2.34
1.46

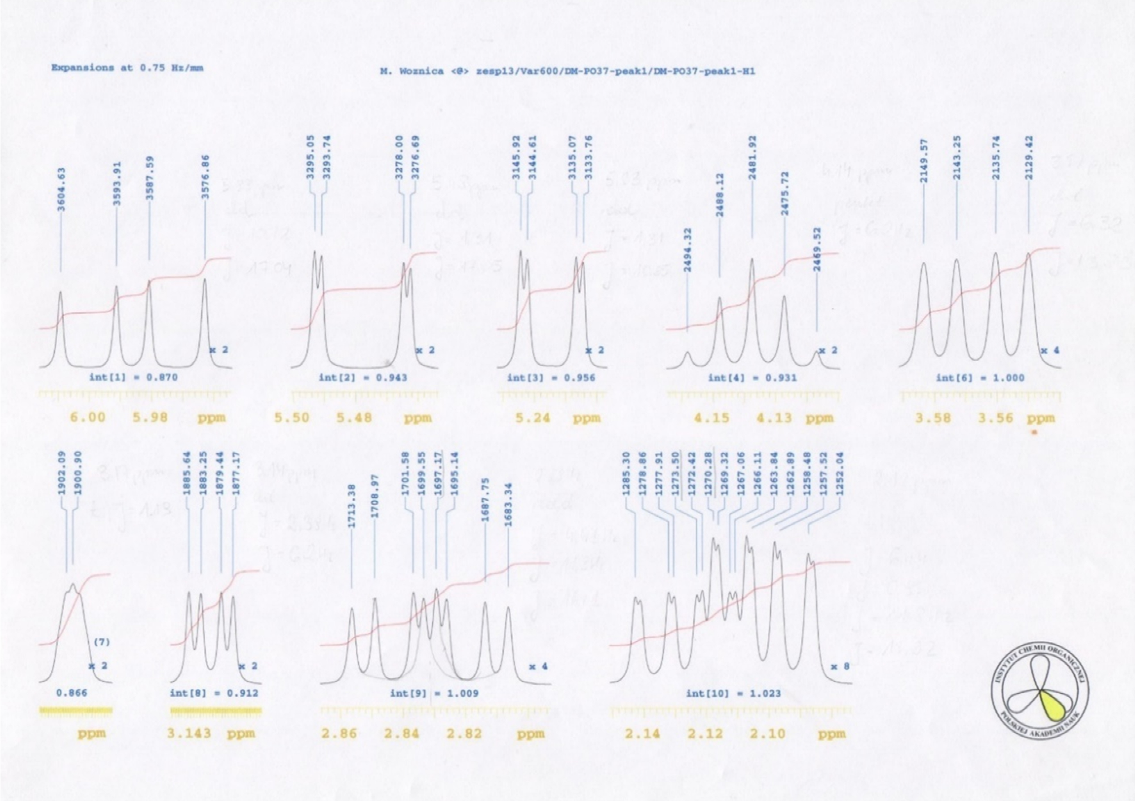
Expansions at 0.75 Hz/mm
M. Woznica <@> zesp13/Var600/DM-PO37-peak1/DM-PO37-peak1-H1
int[1] = 0.870
6.00 5.98 ppm
int[2] = 0.943
5.50 5.48 ppm
int[3] = 0.956
5.24 ppm
int[4] = 0.931
4.15 4.13 ppm
int[6] = 1.000
3.58 3.56 ppm
0.866
ppm
int[8] = 0.912
3.143 ppm
int[9] = 1.009
2.86 2.84 2.82 ppm
int[10] = 1.023
2.14 2.12 2.10 ppm

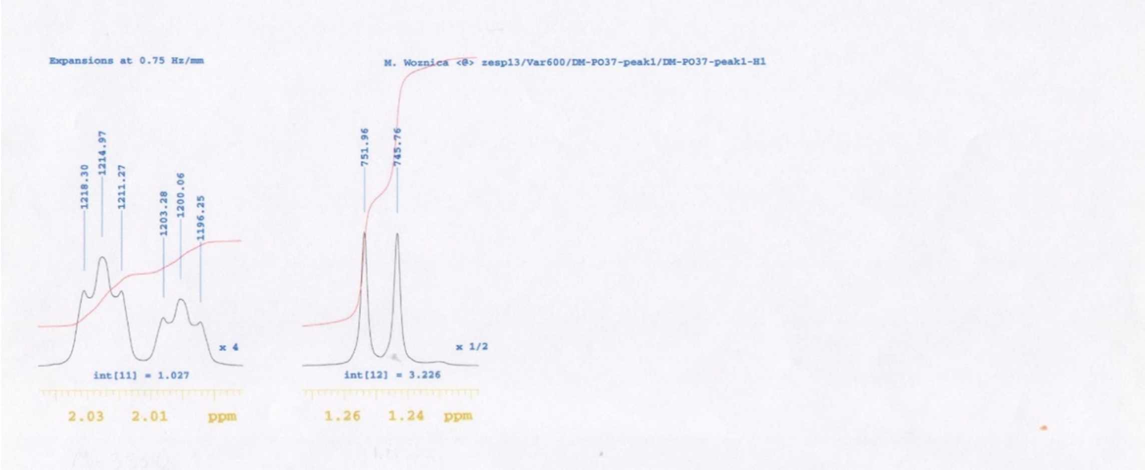
Expansions at 0.75 Hz/mm
M. Woznica <@> zesp13/Var600/DM-PO37-peak1/DM-PO37-peak1-H1
int[11] = 1.027
2.03 2.01 ppm
int[12] = 3.226
1.26 1.24 ppm

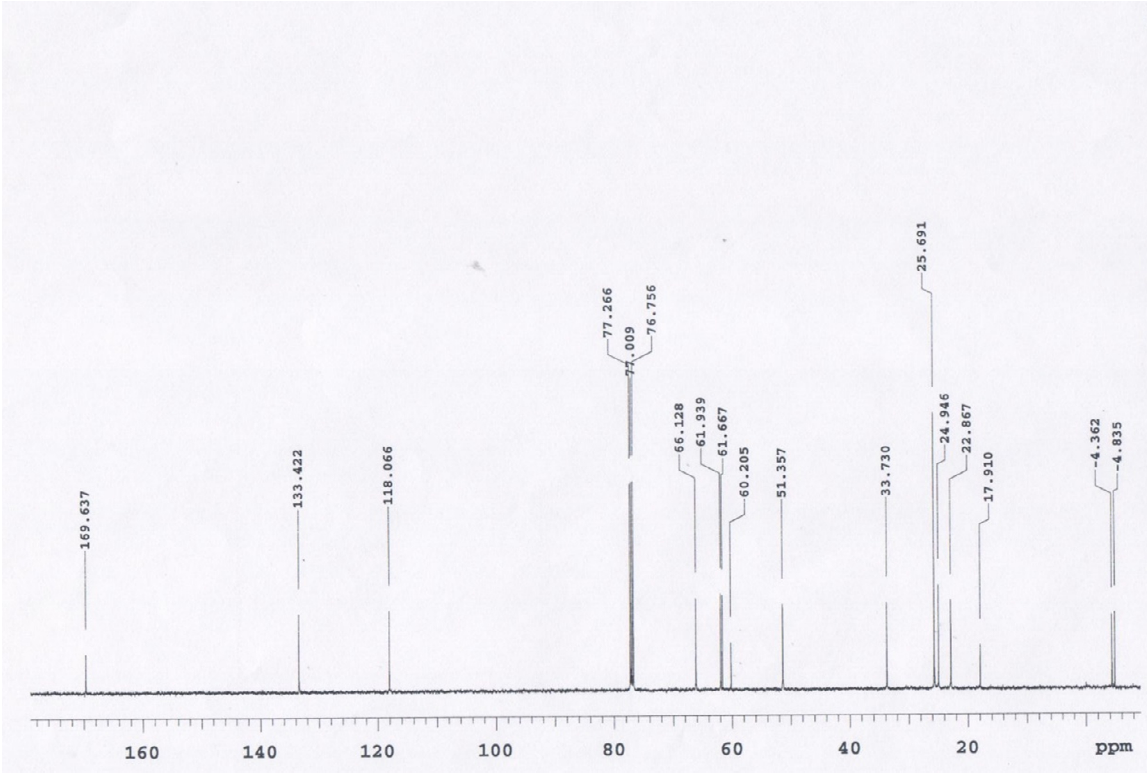
169.637
133.422
118.066
77.266
77.009
76.756
66.128
61.939
61.667
60.205
51.357
33.730
25.691
24.946
22.867
17.910
-4.362
-4.835
160
140
120
100
80
60
40
20
ppm

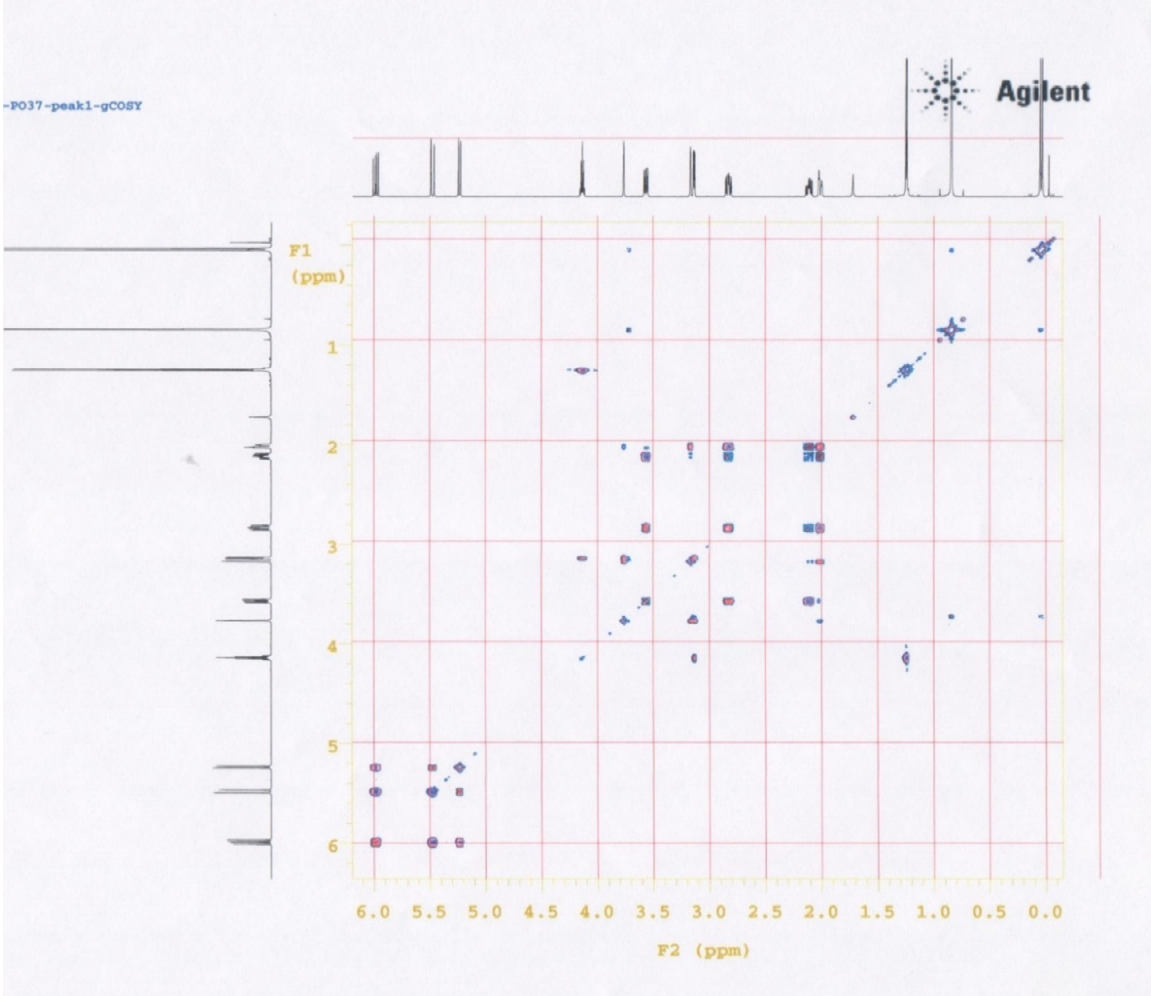
-P037-peak1-gCOSY
Agilent
F1 (ppm)
F2 (ppm)

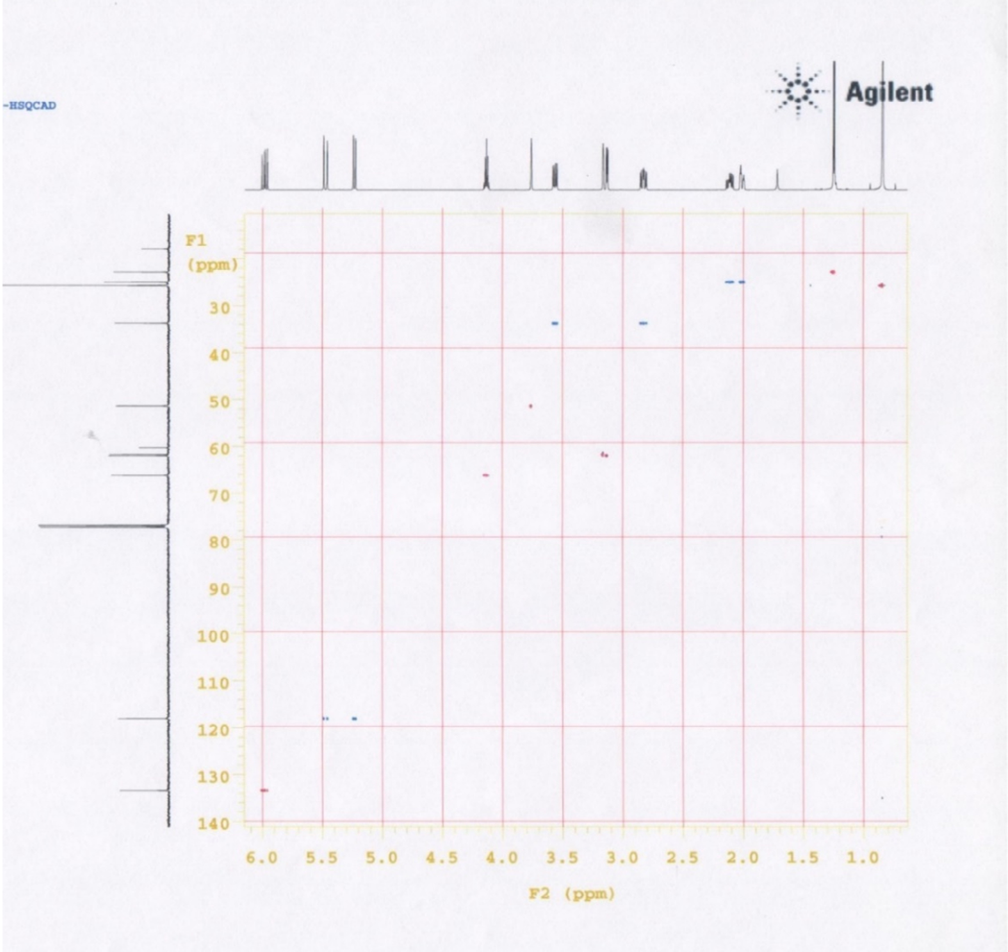

-HSQCAD
Agilent
F1 (ppm)
F2 (ppm)

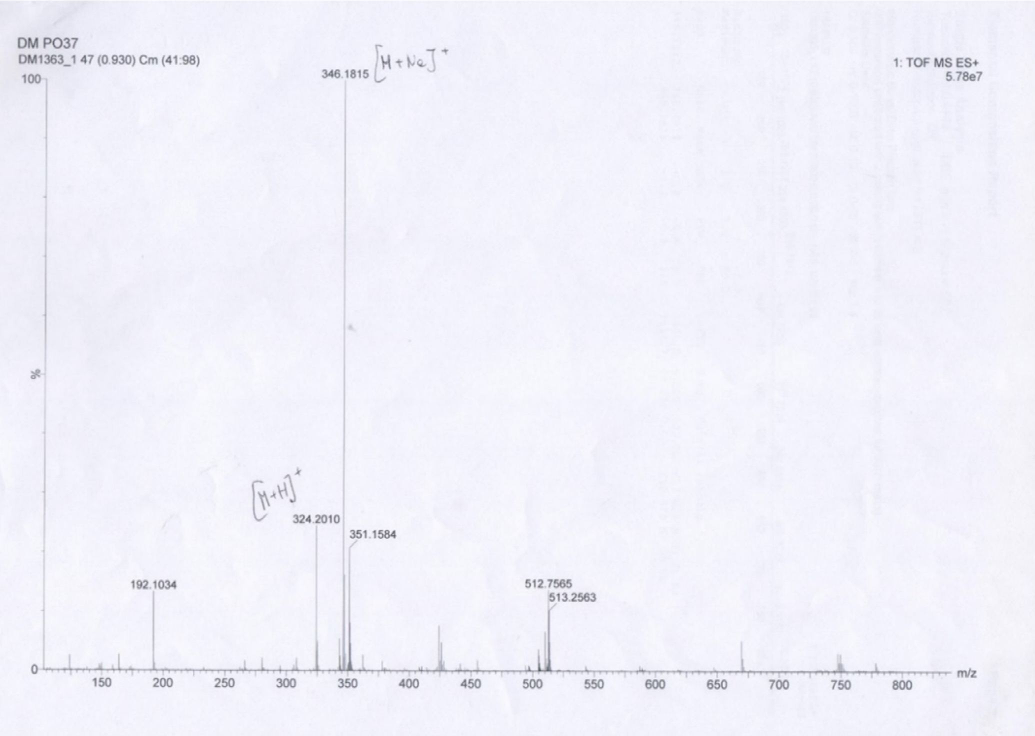

DM PO37
DM1363_1 47 (0.930) Cm (41:98)
1: TOF MS ES+
5.78e7
346.1815 [M+Na]+
[M+H]+
324.2010
351.1584
192.1034
512.7565
513.2563
m/z

5. NMR and MS spectra of Comp. 3

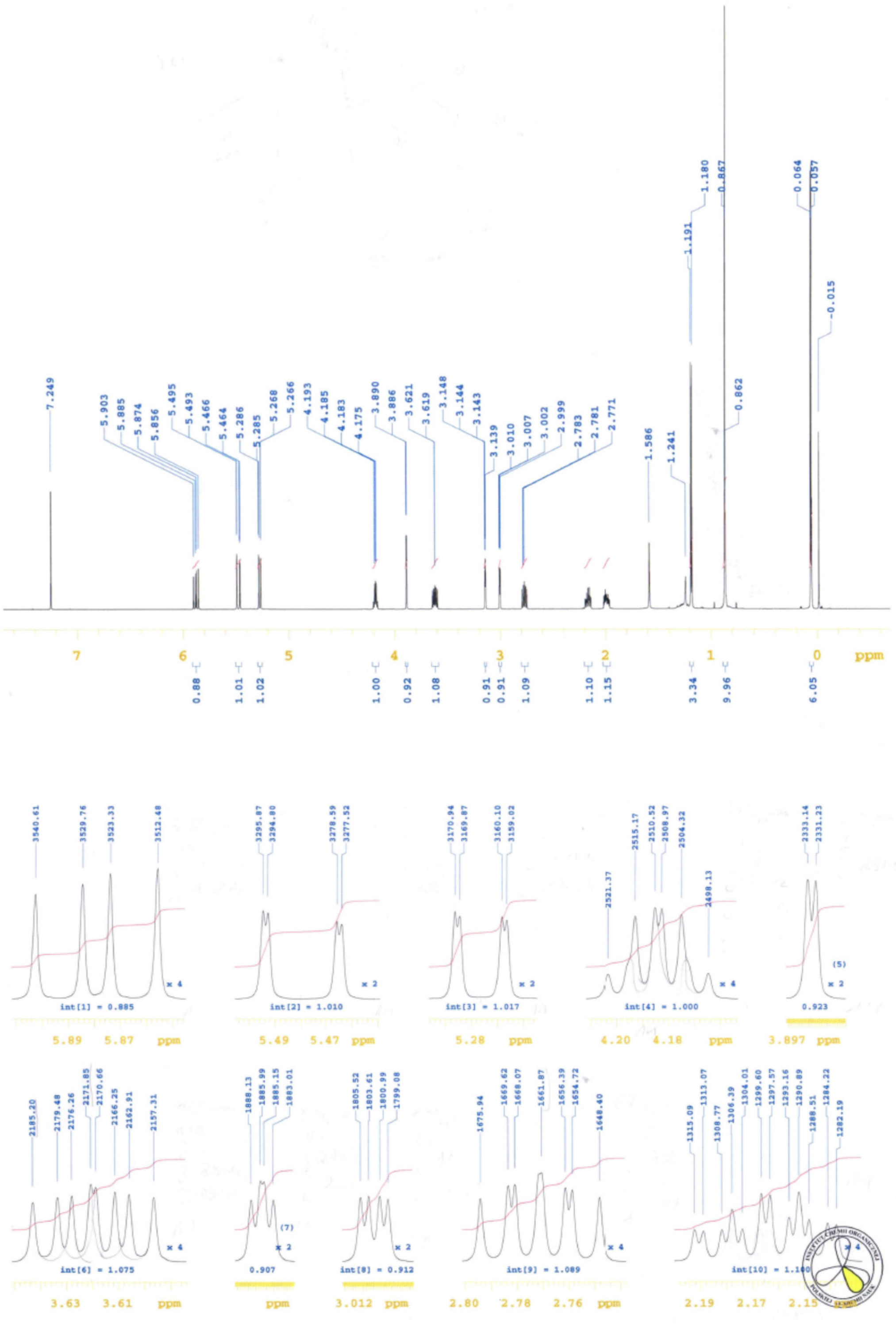

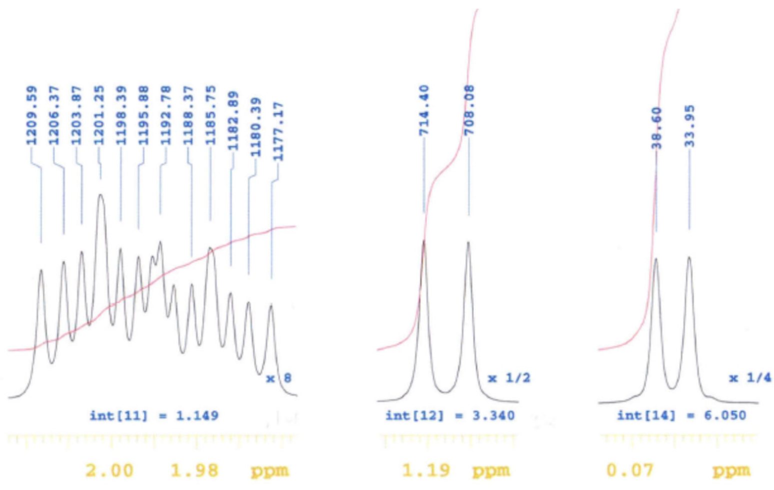
1209.59
1206.37
1203.87
1201.25
1198.39
1195.88
1192.78
1188.37
1185.75
1182.89
1180.39
1177.17
x 8
int[11] = 1.149
2.00 1.98 ppm
714.40
708.08
x 1/2
int[12] = 3.340
1.19 ppm
38.60
33.95
x 1/4
int[14] = 6.050
0.07 ppm

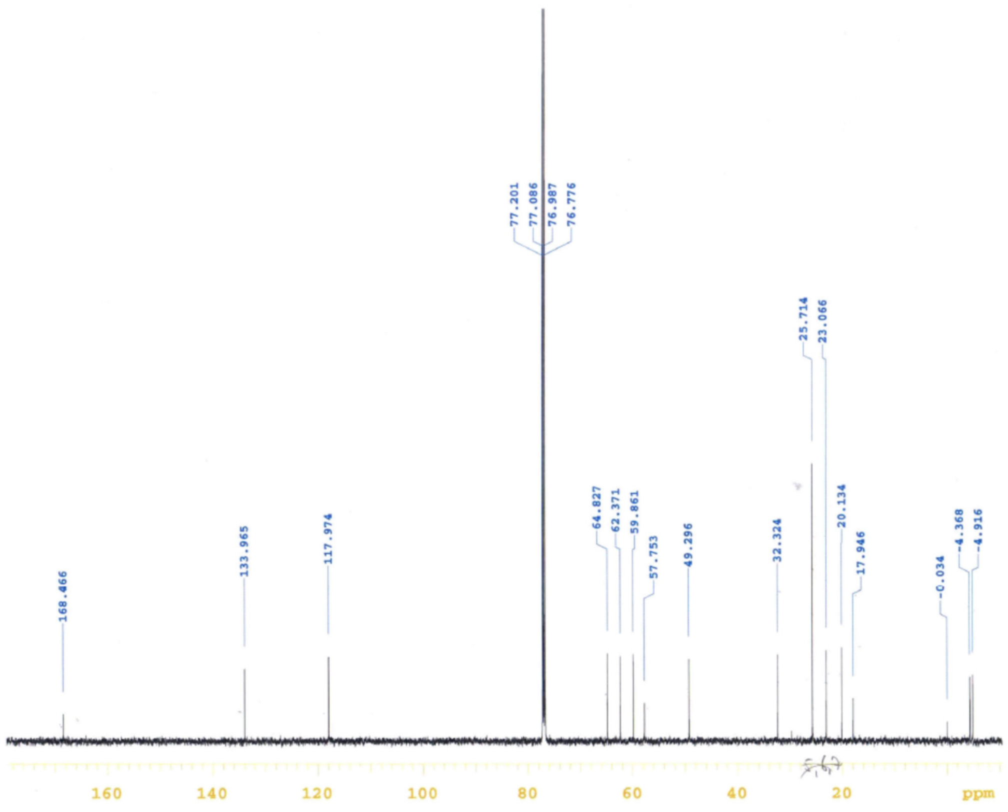
77.201
77.086
76.987
76.776
25.714
23.066
64.827
62.371
59.861
57.753
49.296
32.324
20.134
17.946
168.466
133.965
117.974
-0.034
-4.368
-4.916
160 140 120 100 80 60 40 20 ppm

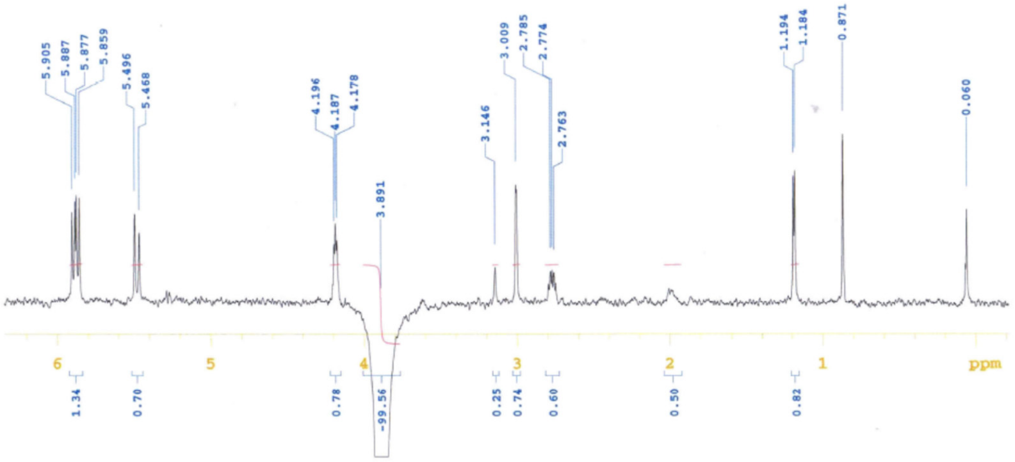
5.905
5.887
5.877
5.859
5.496
5.468
4.196
4.187
4.178
3.891
3.146
3.009
2.785
2.774
2.763
1.194
1.184
0.871
0.060
6
5
4
3
2
1
ppm
1.34
0.70
0.78
-99.56
0.25
0.74
0.60
0.50
0.82

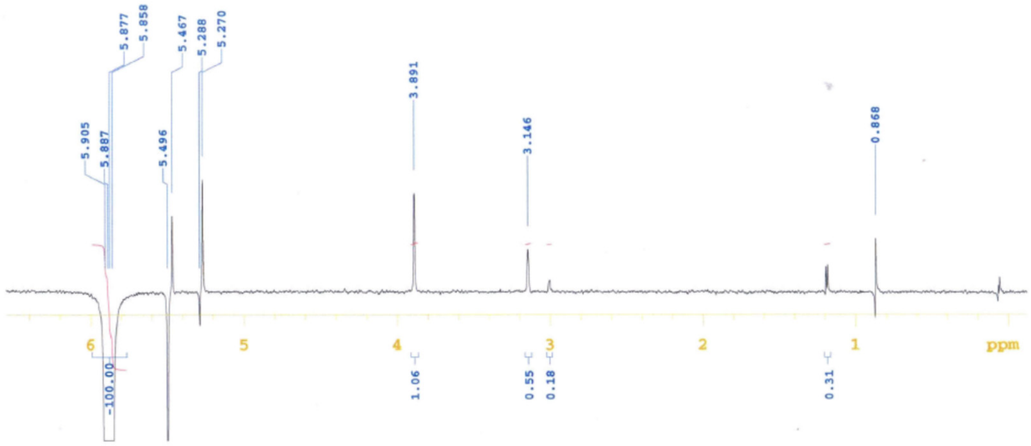
5.905
5.887
5.877
5.858
5.496
5.467
5.288
5.270
3.891
3.146
0.868
6
5
4
3
2
1
ppm
-100.00
1.06
0.55
0.18
0.31

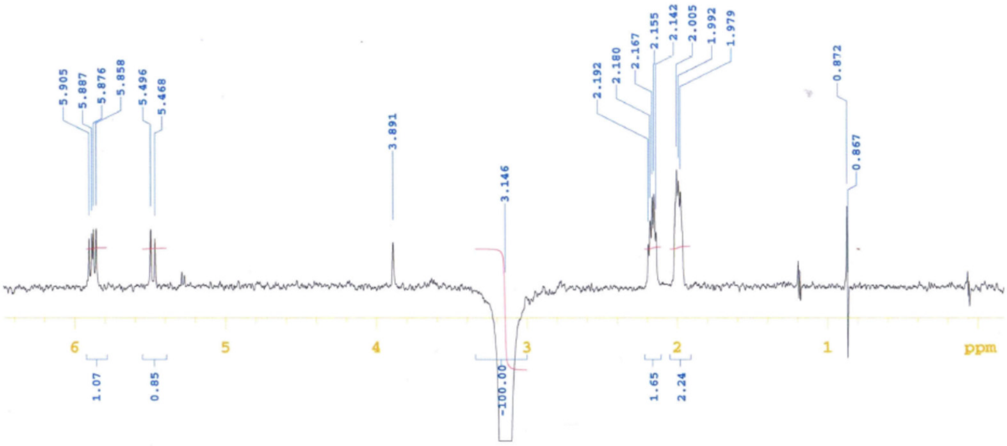
5.905
5.887
5.876
5.858
5.496
5.468
3.891
3.146
2.192
2.180
2.167
2.155
2.142
2.005
1.992
1.979
0.872
0.867
6
5
4
3
2
1
ppm
1.07
0.85
-100.00
1.65
2.24

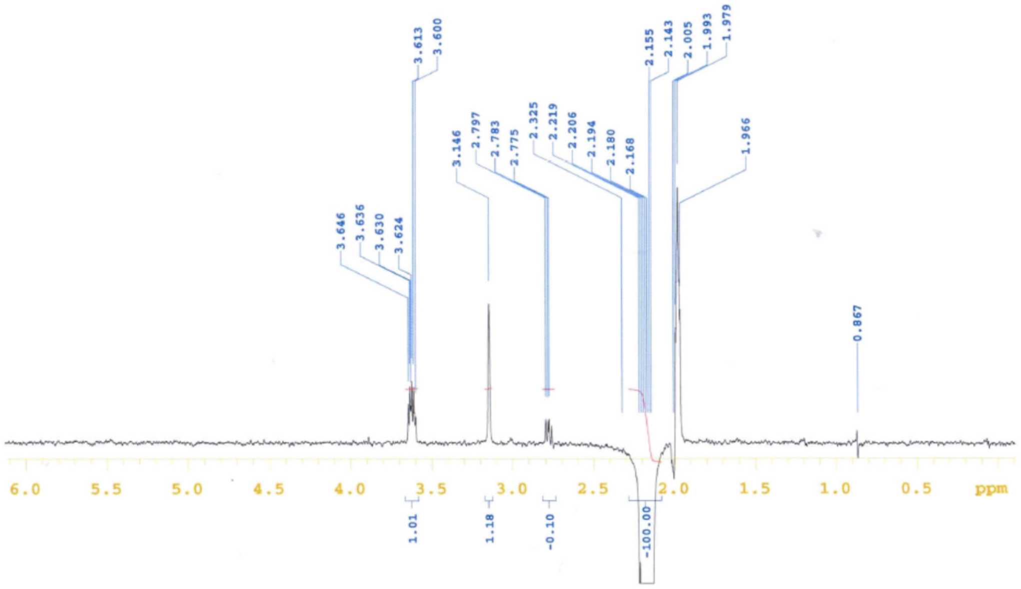
3.646
3.636
3.630
3.624
3.613
3.600
3.146
2.797
2.783
2.775
2.325
2.219
2.206
2.194
2.180
2.168
2.155
2.143
2.005
1.993
1.979
1.966
0.867
6.0 5.5 5.0 4.5 4.0 3.5 3.0 2.5 2.0 1.5 1.0 0.5 ppm
1.01
1.18
-0.10
-100.00

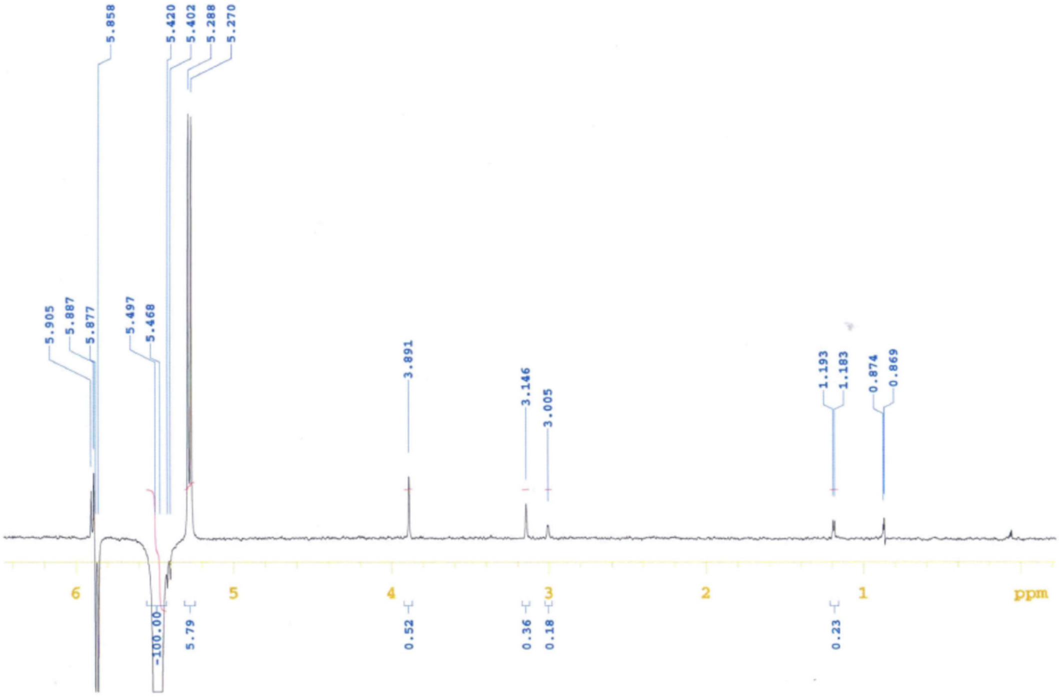
5.905
5.887
5.877
5.858
5.497
5.468
5.420
5.402
5.288
5.270
3.891
3.146
3.005
1.193
1.183
0.874
0.869
6 5 4 3 2 1 ppm
-100.00
5.79
0.52
0.36
0.18
0.23

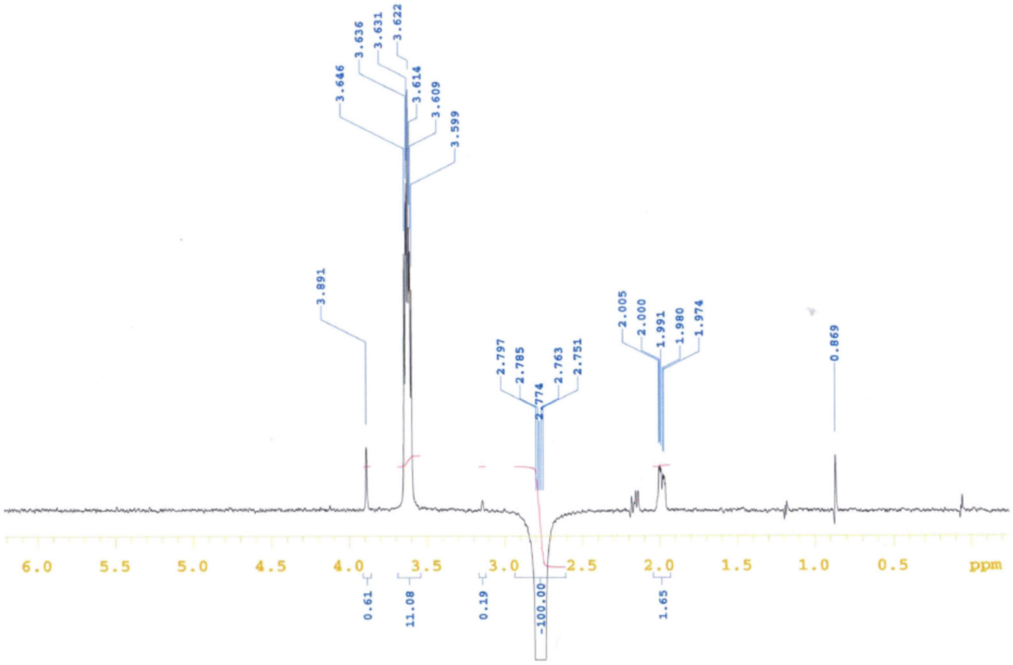
3.646
3.636
3.631
3.622
3.614
3.609
3.599
3.891
2.797
2.785
2.774
2.763
2.751
2.005
2.000
1.991
1.980
1.974
0.869
6.0 5.5 5.0 4.5 4.0 3.5 3.0 2.5 2.0 1.5 1.0 0.5 ppm
0.61
11.08
0.19
-100.00
1.65

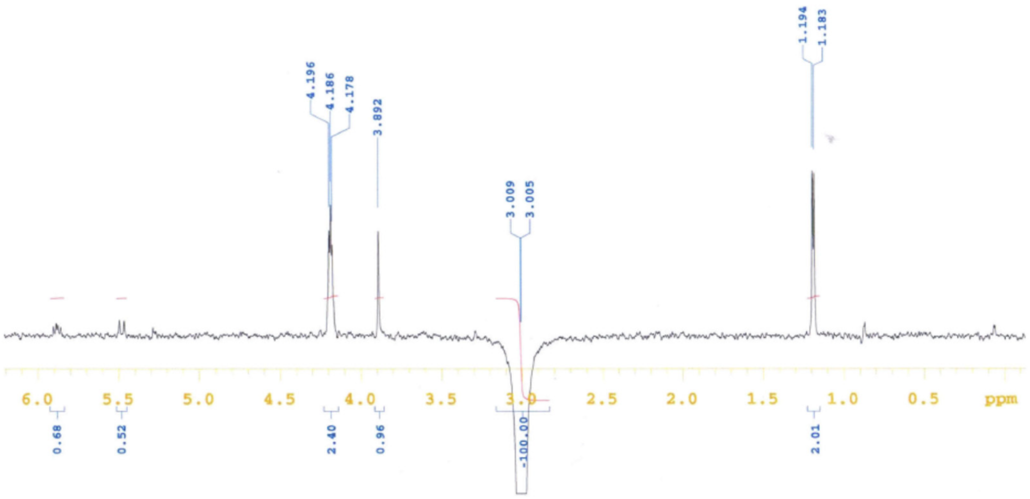
4.196
4.186
4.178
3.892
3.009
3.005
1.194
1.183
6.0 5.5 5.0 4.5 4.0 3.5 3.0 2.5 2.0 1.5 1.0 0.5 ppm
0.68
0.52
2.40
0.96
-100.00
2.01

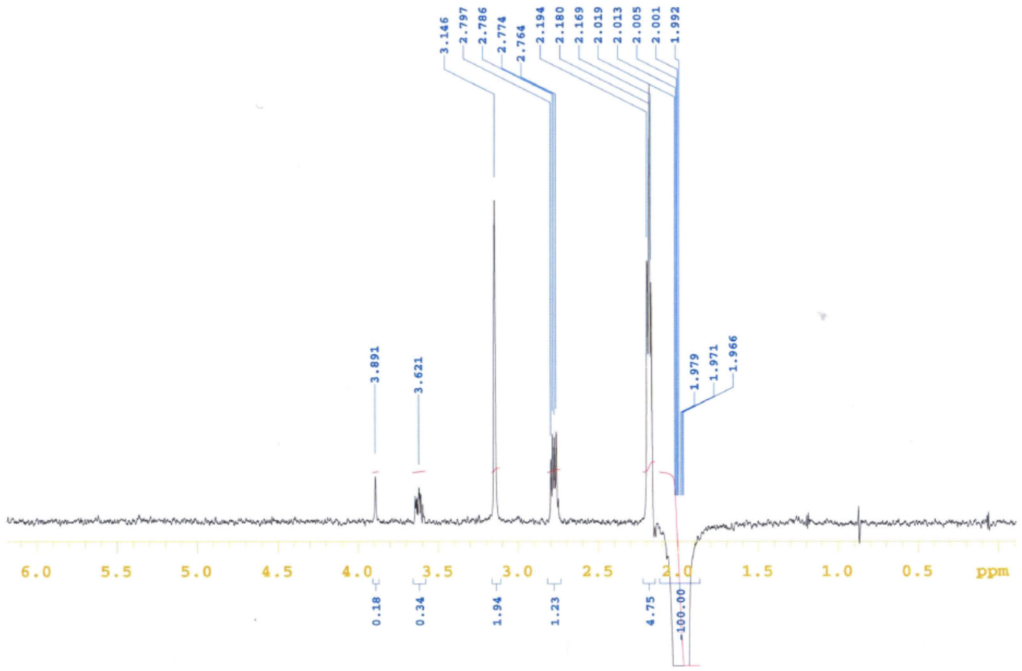
3.146
2.797
2.786
2.774
2.764
2.194
2.180
2.169
2.019
2.013
2.005
2.001
1.992
3.891
3.621
1.979
1.971
1.966
6.0
5.5
5.0
4.5
4.0
3.5
3.0
2.5
2.0
1.5
1.0
0.5
ppm
0.18
0.34
1.94
1.23
4.75
-100.00

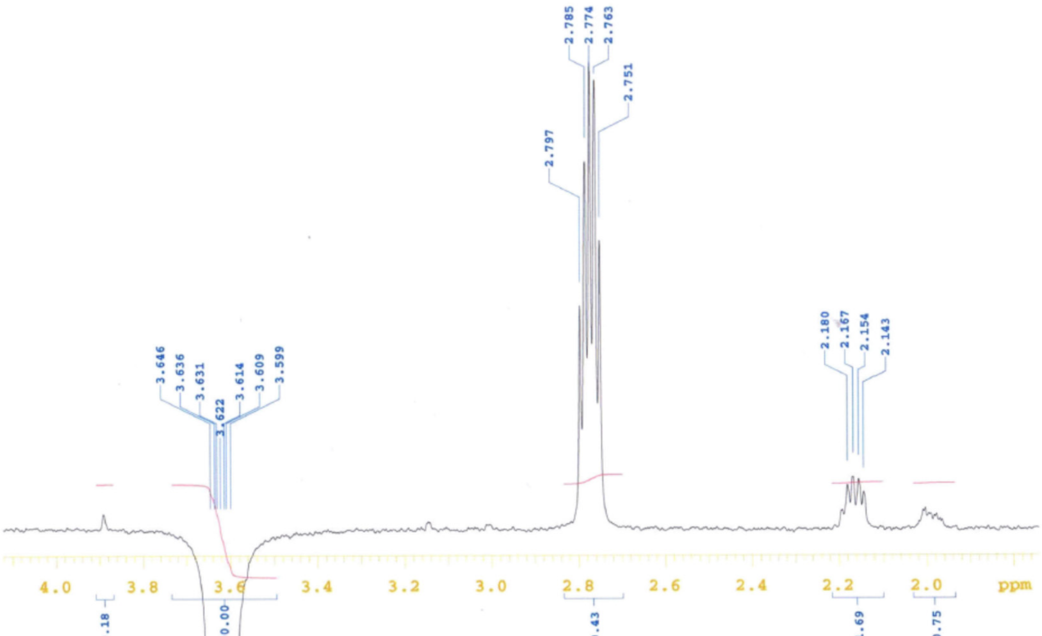
2.785
2.774
2.763
2.751
2.797
3.646
3.636
3.631
3.622
3.614
3.609
3.599
2.180
2.167
2.154
2.143
4.0
3.8
3.6
3.4
3.2
3.0
2.8
2.6
2.4
2.2
2.0
ppm
0.18
-100.00
10.43
1.69
0.75

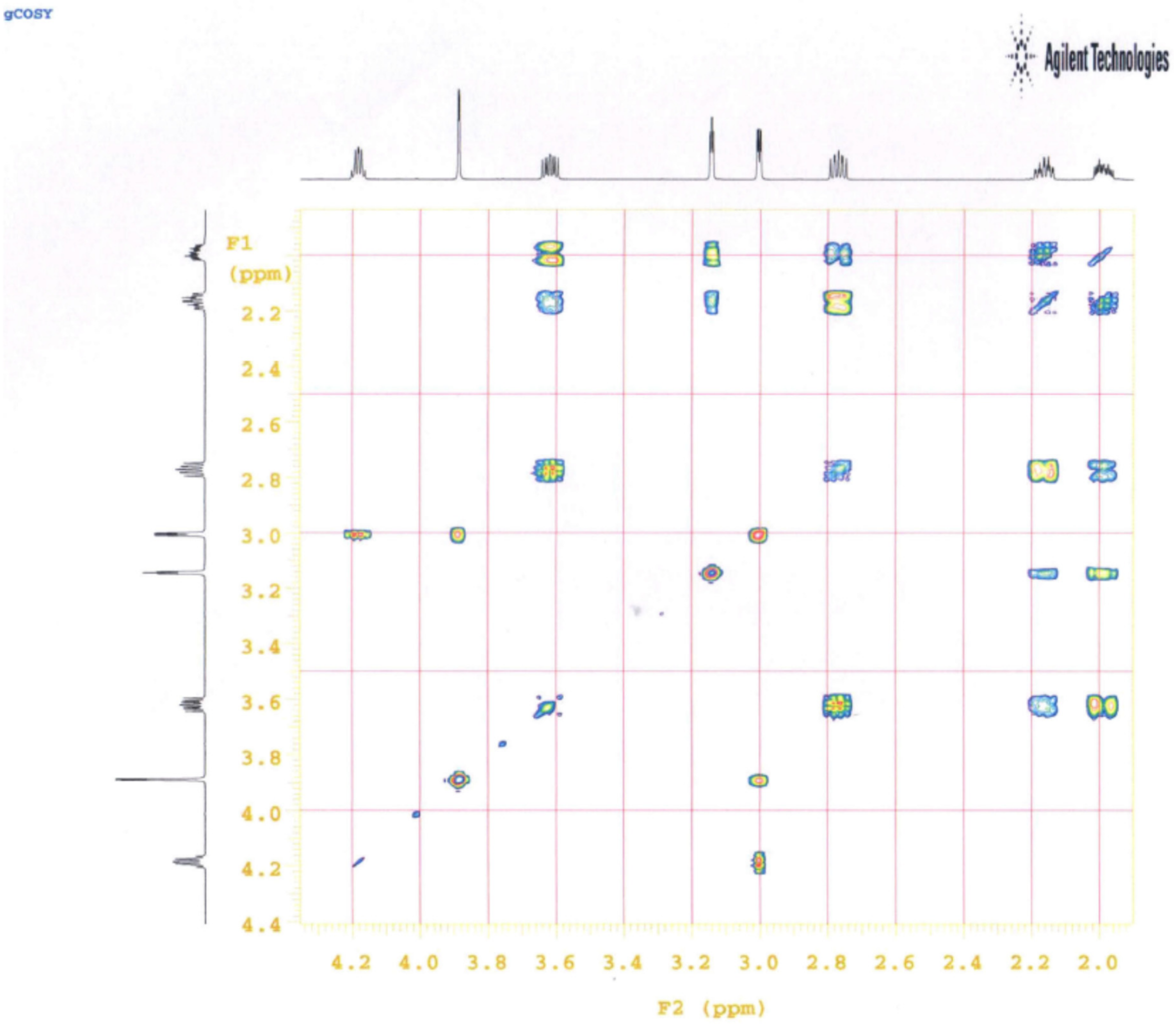
gCOSY
Agilent Technologies
F1
(ppm)
2.2
2.4
2.6
2.8
3.0
3.2
3.4
3.6
3.8
4.0
4.2
4.4
4.2 4.0 3.8 3.6 3.4 3.2 3.0 2.8 2.6 2.4 2.2 2.0
F2 (ppm)

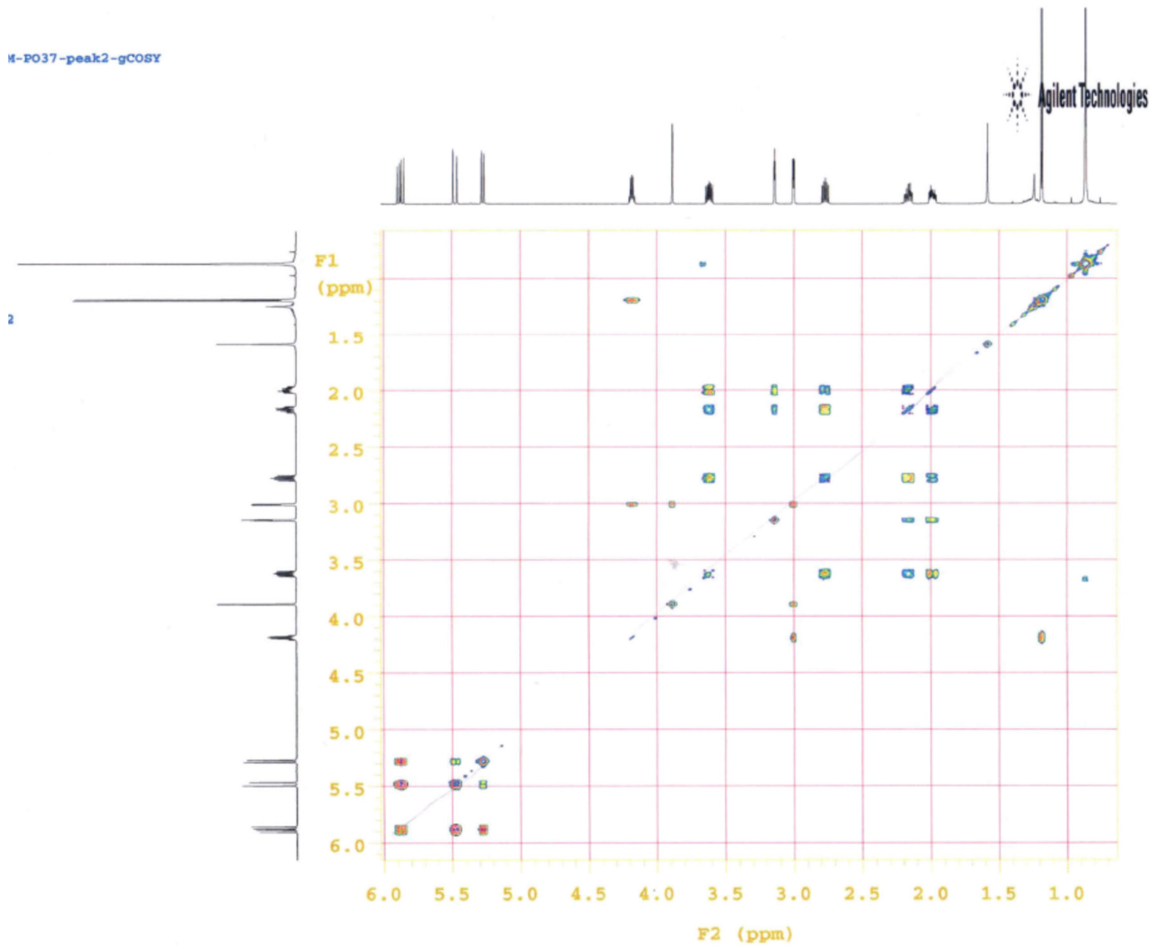
M-PO37-peak2-gCOSY
Agilent Technologies
F1
(ppm)
F2 (ppm)

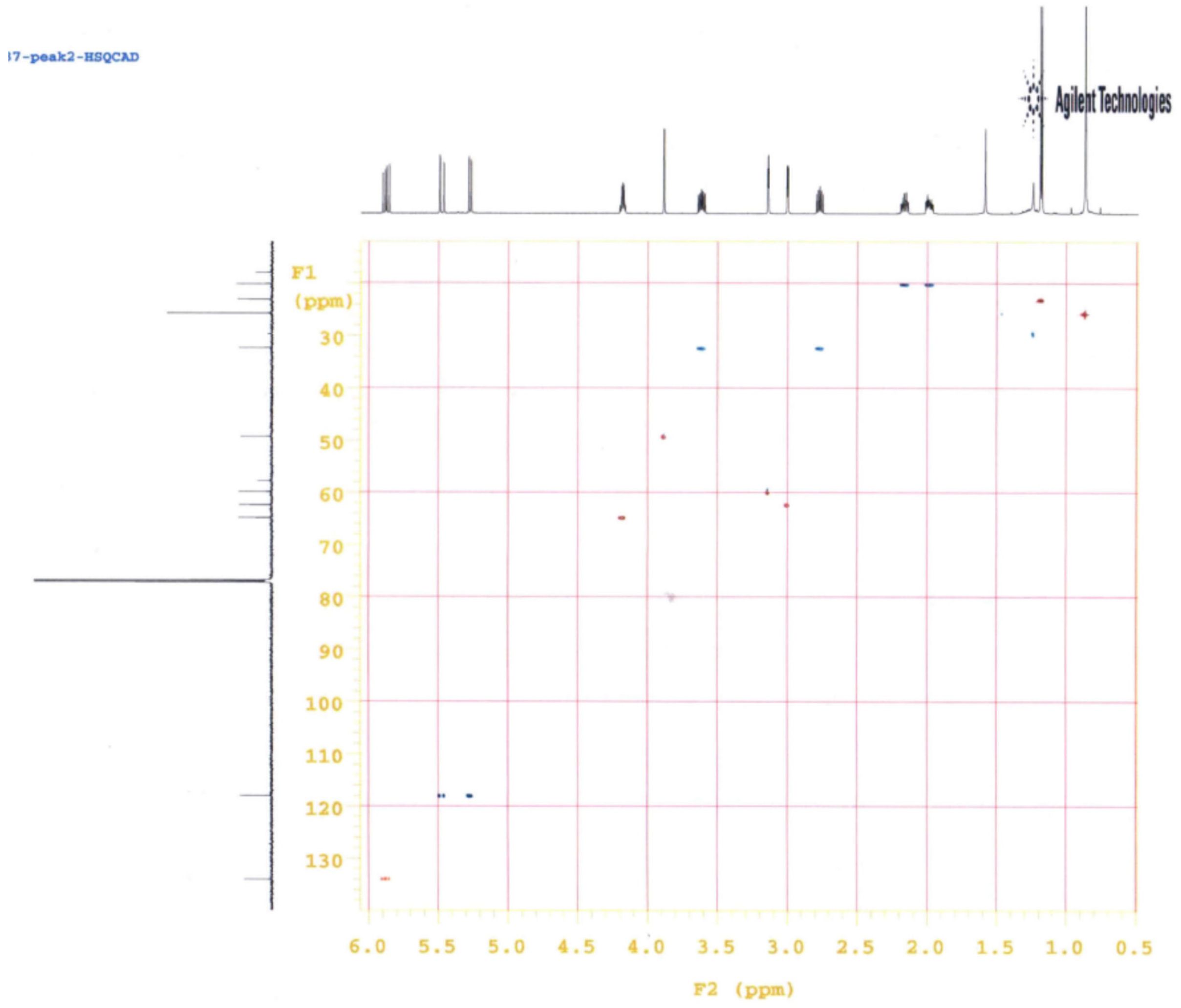

)7-peak2-HSQCAD
Agilent Technologies
F1 (ppm)
30
40
50
60
70
80
90
100
110
120
130
6.0 5.5 5.0 4.5 4.0 3.5 3.0 2.5 2.0 1.5 1.0 0.5
F2 (ppm)

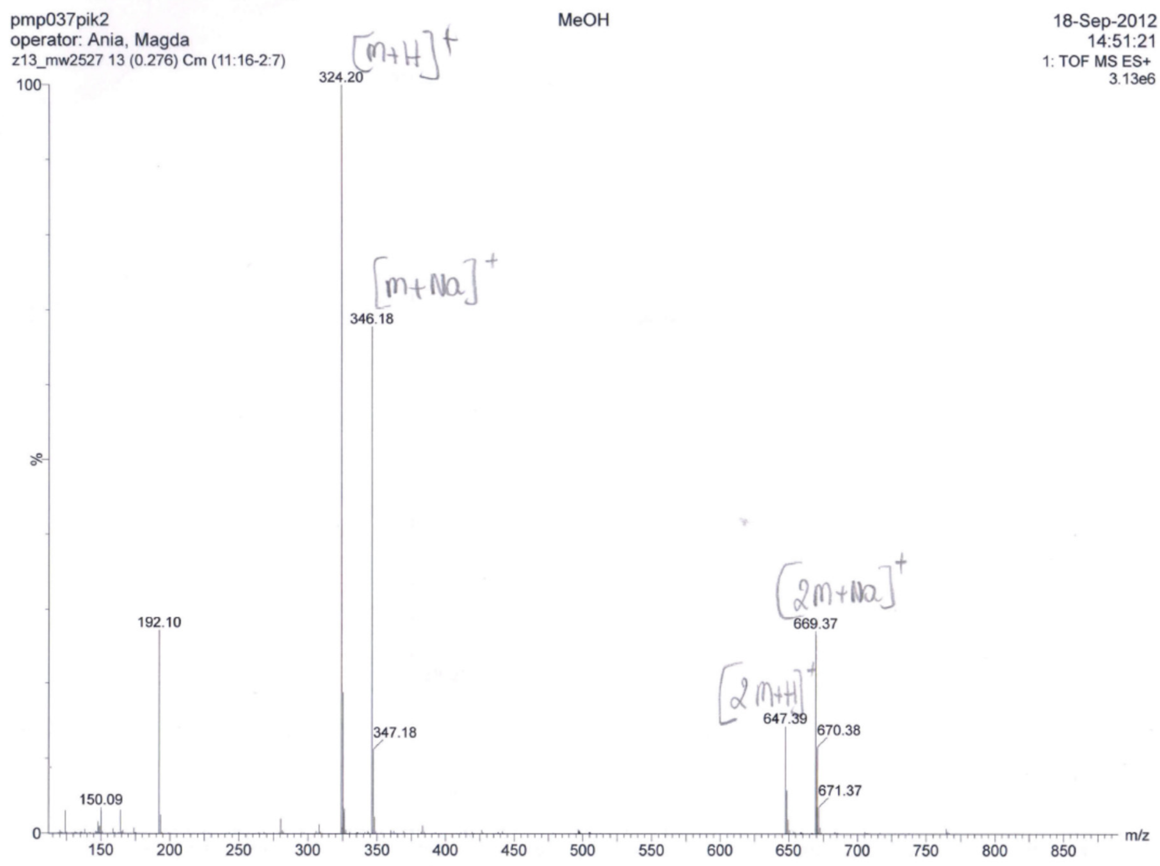
pmp037pik2
operator: Ania, Magda
z13_mw2527 13 (0.276) Cm (11:16-2:7)
MeOH
18-Sep-2012
14:51:21
1: TOF MS ES+
3.13e6
[M+H]+
324.20
[M+Na]+
346.18
347.18
192.10
150.09
[2M+Na]+
669.37
[2M+H]+
647.39
670.38
671.37
%
m/z

6. Coordinates of calculated structures

Conf. 1 up a

C	0.809997	1.521488	-0.857367
C	2.139914	2.109667	-0.313227
C	1.501671	0.195476	-0.615206
N	2.660426	0.853974	-0.160754
C	1.377653	-1.119792	-0.692938
C	2.561441	-1.940747	-0.315020
C	3.800189	-1.270331	0.249980
C	3.810719	0.243047	0.469969
O	2.581557	3.208883	-0.072149
C	-0.431188	1.803997	-0.017373
C	-0.914202	3.234820	-0.194195
O	-1.432169	0.887277	-0.419441
Si	-2.378940	-0.006109	0.644823
C	-3.426285	-1.124345	-0.468136
C	-3.437174	1.161433	1.659655
C	-1.292307	-1.007367	1.797120
H	0.624621	1.751949	-1.909359
H	0.472898	-1.586868	-1.057545
H	4.730208	0.685668	0.075917
H	3.772901	0.444552	1.545486
H	-0.170432	1.640719	1.038882
H	-1.795665	3.415200	0.424734
H	-0.132097	3.941251	0.095304
H	-1.179341	3.414051	-1.239731
H	-2.807993	1.811382	2.276194
H	-4.058994	1.796259	1.022414
H	-4.094613	0.603132	2.333141
H	-0.637347	-0.359505	2.387428
H	-0.661187	-1.708616	1.246524
H	-1.912938	-1.574635	2.497845
C	3.831131	-1.818028	-1.148669
C	4.577891	-2.012214	1.314495
H	4.422066	-2.708412	-1.332841
H	3.791014	-1.112573	-1.972326
H	2.344682	-2.926565	0.082791
H	4.601645	-3.083897	1.104498
H	4.126118	-1.867495	2.300557
H	5.612318	-1.655755	1.358357
C	-2.512120	-2.004297	-1.334959
H	-1.867517	-1.398607	-1.978599
H	-1.872749	-2.650410	-0.724352
H	-3.115874	-2.654630	-1.980529
C	-4.321108	-0.269305	-1.379232
H	-4.930358	-0.915226	-2.024095

H	-5.006376	0.357942	-0.800313
H	-3.727969	0.384530	-2.025160
C	-4.310982	-2.023950	0.411461
H	-4.983583	-1.438612	1.046894
H	-4.934036	-2.670237	-0.219187
H	-3.713772	-2.673477	1.059410

Conf. 1 up b

C	0.787459	1.524192	-0.865726
C	1.865023	2.181315	0.040001
C	1.651383	0.290854	-0.697196
N	2.560430	1.006068	0.106155
C	1.798854	-0.982061	-1.027545
C	3.022679	-1.678454	-0.542398
C	3.983716	-0.962153	0.387926
C	3.664915	0.456337	0.862021
O	2.048257	3.266056	0.541034
C	-0.616994	1.480228	-0.264910
C	-1.294141	2.835409	-0.397005
O	-1.360369	0.486127	-0.942289
Si	-2.133918	-0.840320	-0.253122
C	-3.781999	-0.303641	0.529397
C	-1.048517	-1.636141	1.051840
C	-2.418673	-2.001675	-1.687945
H	0.760534	1.923514	-1.882539
H	1.086919	-1.495449	-1.659828
H	4.541214	1.103879	0.765252
H	3.387336	0.420867	1.920556
H	-0.523388	1.224240	0.799946
H	-2.274060	2.827427	0.083434
H	-0.683724	3.612507	0.070109
H	-1.428773	3.081082	-1.453833
H	-0.158576	-2.079077	0.599293
H	-0.718264	-0.920416	1.809898
H	-1.604800	-2.427344	1.564652
H	-1.472258	-2.231591	-2.185451
H	-3.094861	-1.565428	-2.428427
H	-2.856167	-2.943040	-1.343339
C	4.379649	-1.196410	-1.041851
C	4.677866	-1.783037	1.452425
H	5.152437	-1.930801	-1.240367
H	4.362069	-0.351623	-1.722895
H	2.923280	-2.746396	-0.379239
H	4.937322	-2.773598	1.072052
H	4.035599	-1.911135	2.328969
H	5.602799	-1.294739	1.776264
C	-4.582273	0.562136	-0.455771

H	-4.045128	1.476836	-0.721663
H	-4.802700	0.023863	-1.383480
H	-5.540899	0.854572	-0.008703
C	-3.524846	0.492961	1.819279
H	-4.476313	0.816569	2.259956
H	-3.002571	-0.108780	2.569272
H	-2.928694	1.392879	1.639345
C	-4.605448	-1.556062	0.878105
H	-4.072180	-2.220878	1.565547
H	-5.543318	-1.263849	1.367058
H	-4.866007	-2.132329	-0.015033

Conf. 1 up c

C	-0.644731	1.806906	0.678961
C	-1.764832	2.232363	-0.311139
C	-1.425289	0.509151	0.727076
N	-2.360430	1.007456	-0.199975
C	-1.513068	-0.681772	1.297081
C	-2.679362	-1.532498	0.933271
C	-3.661442	-1.061662	-0.122499
C	-3.429646	0.270633	-0.837334
O	-2.032536	3.212493	-0.966160
C	0.746564	1.768473	0.048680
C	1.315803	3.177289	-0.049242
O	1.571864	0.930729	0.838457
Si	2.828928	-0.036566	0.266753
C	2.182001	-1.625211	-0.563253
C	3.816175	-0.452601	1.796278
C	3.868382	0.936310	-0.954456
H	-0.634686	2.368177	1.616243
H	-0.796792	-1.014525	2.035301
H	-4.343894	0.871553	-0.842576
H	-3.152154	0.071253	-1.877298
H	0.648381	1.351525	-0.962682
H	2.277772	3.173244	-0.562977
H	0.630009	3.821693	-0.605391
H	1.455121	3.594641	0.951773
H	4.298466	0.442342	2.199032
H	3.174189	-0.874766	2.574110
H	4.596167	-1.185385	1.568120
H	4.239864	1.857765	-0.497707
H	3.306749	1.202032	-1.855385
H	4.734958	0.345722	-1.265651
C	-4.076044	-1.063666	1.321725
C	-4.270528	-2.105677	-1.032212
H	-4.803697	-1.801966	1.640080
H	-4.131792	-0.110083	1.836740
H	-2.508551	-2.603533	0.966245

H	-4.479494	-3.026843	-0.483608
H	-3.593800	-2.346565	-1.857679
H	-5.213243	-1.746709	-1.457960
C	1.060520	-1.321692	-1.570337
H	0.169408	-0.930482	-1.071778
H	1.375357	-0.600938	-2.332722
H	0.769324	-2.242699	-2.091855
C	1.649563	-2.601976	0.497059
H	1.255789	-3.504450	0.012230
H	2.435427	-2.915847	1.190697
H	0.838116	-2.161221	1.080644
C	3.348231	-2.299191	-1.309787
H	4.199869	-2.501551	-0.651112
H	3.017600	-3.260569	-1.722546
H	3.705135	-1.688925	-2.144605

Conf. 1 down a

C	0.840546	1.588357	-0.835360
C	2.185831	2.113079	-0.264876
C	1.509730	0.236721	-0.695637
N	2.659454	0.835641	-0.144647
C	1.358578	-1.068239	-0.856570
C	2.448033	-1.949098	-0.351321
C	3.726789	-1.336089	0.188002
C	3.916462	0.180300	0.141788
C	2.738727	-1.937668	1.145973
O	2.672292	3.190018	-0.009888
C	-0.392962	1.830343	0.028126
C	-0.864716	3.273138	-0.059471
O	-1.406619	0.947393	-0.415188
Si	-2.328629	-0.011115	0.613704
C	-1.213360	-1.062660	1.692753
C	-3.390535	-1.075919	-0.537800
C	-3.377935	1.093596	1.705494
H	0.661141	1.900140	-1.867329
H	0.450774	-1.491314	-1.264396
C	5.020427	-2.084237	-0.046958
H	4.320592	0.550224	1.088639
H	4.634549	0.423190	-0.648631
H	2.125453	-1.268537	1.740801
H	3.007939	-2.868832	1.632193
H	-0.123042	1.604284	1.070472
H	-1.733621	3.426161	0.584114
H	-1.146043	3.512483	-1.088601
H	-0.071383	3.956014	0.255308
H	-1.817224	-1.677892	2.367193
H	-0.578101	-1.721181	1.096078
H	-0.561129	-0.439083	2.311731

H	-4.023087	1.745552	1.110162
H	-4.011538	0.496874	2.368761
H	-2.743668	1.726234	2.334668
H	2.562296	-2.902728	-0.855518
H	5.775924	-1.790097	0.688883
H	5.418653	-1.877130	-1.044823
H	4.868022	-3.162085	0.042622
C	-2.489867	-1.935868	-1.437986
H	-1.849663	-1.315549	-2.072225
H	-1.847296	-2.601443	-0.852340
H	-3.103474	-2.564995	-2.095143
C	-4.278760	-0.180670	-1.415782
H	-4.901101	-0.798377	-2.075674
H	-4.951473	0.438424	-0.813781
H	-3.679978	0.483765	-2.045271
C	-4.282441	-1.995862	0.313423
H	-4.954033	-1.424850	0.962596
H	-4.906564	-2.621204	-0.336931
H	-3.690962	-2.666563	0.944944

Conf. 1 down b

C	0.813295	1.587212	-0.843526
C	1.920873	2.189960	0.063337
C	1.668735	0.339964	-0.757990
N	2.573290	0.988199	0.105454
C	1.788304	-0.920184	-1.144504
C	2.904534	-1.711226	-0.556059
C	3.937640	-1.027264	0.319678
C	3.857352	0.483473	0.541181
C	2.915433	-1.925521	0.954359
O	2.161237	3.265586	0.559355
C	-0.584370	1.524668	-0.229373
C	-1.254604	2.888290	-0.289333
O	-1.343603	0.568442	-0.942817
Si	-2.100636	-0.794120	-0.306164
C	-2.409714	-1.882208	-1.792174
C	-3.735264	-0.301433	0.531748
C	-0.991027	-1.651629	0.938146
H	0.780185	2.040784	-1.837299
H	1.065925	-1.387514	-1.800073
C	5.364228	-1.524775	0.248364
H	4.014501	0.728153	1.595613
H	4.648167	0.969456	-0.040131
H	2.099538	-1.464999	1.501760
H	3.250859	-2.879935	1.344788
H	-0.479921	1.218365	0.821050
H	-2.231595	2.861040	0.196308
H	-1.394595	3.188074	-1.331404

H	-0.637417	3.638218	0.212231
H	-2.857825	-2.833321	-1.490752
H	-3.084176	-1.401281	-2.506065
H	-1.469426	-2.100621	-2.306242
H	-0.620257	-0.965481	1.704912
H	-1.547554	-2.445136	1.446988
H	-0.126411	-2.101314	0.445473
H	3.273770	-2.533325	-1.159744
H	5.907590	-1.275086	1.165615
H	5.894334	-1.073531	-0.595767
H	5.392655	-2.610001	0.127241
C	-4.560963	0.597654	-0.401255
H	-4.034925	1.526273	-0.639893
H	-4.796090	0.095111	-1.345191
H	-5.512433	0.864464	0.076044
C	-3.457996	0.444725	1.847477
H	-4.403414	0.734038	2.323600
H	-2.908360	-0.178849	2.559143
H	-2.880616	1.360929	1.690644
C	-4.543649	-1.572536	0.846444
H	-3.995253	-2.256698	1.502127
H	-5.476785	-1.306006	1.358643
H	-4.812507	-2.118772	-0.062915

Conf. 1 down c

C	-0.688417	1.770309	0.671764
C	-1.853860	2.215199	-0.253776
C	-1.472439	0.474629	0.737423
N	-2.426867	0.975494	-0.170760
C	-1.538949	-0.727938	1.286571
C	-2.643496	-1.624538	0.845625
C	-3.731207	-1.090007	-0.067107
C	-3.709184	0.380226	-0.483370
C	-2.704889	-2.035714	-0.621240
O	-2.180524	3.224906	-0.831921
C	0.691455	1.755048	0.020302
C	1.223993	3.175651	-0.111503
O	1.545262	0.954623	0.819034
Si	2.850085	0.033314	0.281076
C	3.878195	1.026715	-0.932903
C	2.271220	-1.585070	-0.539978
C	3.822881	-0.329722	1.833152
H	-0.662777	2.319719	1.616478
H	-0.798237	-1.073560	1.993976
C	-5.136728	-1.614633	0.126543
H	-3.914502	0.483609	-1.552650
H	-4.492523	0.915224	0.064293
H	-1.929408	-1.632605	-1.263663

H	-3.023411	-3.043257	-0.864660
H	0.590741	1.314987	-0.980582
H	2.179474	3.185789	-0.637076
H	1.365220	3.615400	0.879614
H	0.516006	3.792983	-0.670515
H	4.773130	0.463642	-1.213757
H	3.328834	1.256499	-1.851029
H	4.204618	1.968568	-0.483429
H	3.179873	-0.758292	2.606600
H	4.626957	-1.043423	1.629466
H	4.273156	0.584279	2.230171
H	-2.961255	-2.370877	1.565619
H	-5.724672	-1.489107	-0.788507
H	-5.646747	-1.083075	0.935655
H	-5.123644	-2.678790	0.372724
C	1.161727	-1.319204	-1.569645
H	0.259552	-0.933357	-1.087614
H	1.474029	-0.604513	-2.338611
H	0.890626	-2.252925	-2.078979
C	1.739756	-2.561193	0.521323
H	1.382653	-3.480856	0.040425
H	2.515818	-2.843339	1.239150
H	0.901532	-2.134479	1.077135
C	3.468758	-2.236800	-1.254722
H	4.309549	-2.412215	-0.574842
H	3.171811	-3.209727	-1.665992
H	3.829681	-1.625141	-2.086820

Conf. 2a

C	1.160593	1.115798	-0.508411
C	2.492770	1.831198	-0.256916
N	2.914102	0.870774	0.615130
C	1.712660	0.037389	0.461835
C	-0.159502	1.780227	-0.158943
O	-1.182162	0.835929	-0.432246
C	-0.400394	3.049099	-0.959201
C	1.999244	-1.342377	-0.088931
C	3.401825	-1.690193	-0.406855
C	4.541845	-0.714480	-0.236306
C	4.246997	0.345874	0.829115
O	3.039407	2.825063	-0.683444
Si	-2.371822	0.348434	0.649695
C	-3.166559	1.859490	1.421853
C	-3.600710	-0.634902	-0.402457
C	-1.600286	-0.713211	1.986944
C	-4.712752	-1.190551	0.503184
C	-4.225083	0.280993	-1.467183
C	-2.884231	-1.802661	-1.098092

H	1.128638	0.769982	-1.546442
H	1.146618	-0.053168	1.393150
H	-0.145359	2.030239	0.912255
H	-1.362363	3.485684	-0.681795
H	0.386606	3.782777	-0.768964
H	-0.416600	2.822825	-2.028878
H	4.719168	-0.232272	-1.202848
H	5.450644	-1.264581	0.024238
H	4.959391	1.168809	0.769996
H	4.302010	-0.082558	1.831373
H	-3.954826	1.563487	2.120844
H	-2.432008	2.443801	1.984639
H	-3.609838	2.511722	0.664502
H	-2.367284	-1.112748	2.657681
H	-1.050880	-1.553722	1.552905
H	-0.907091	-0.127490	2.598797
H	-5.444222	-1.743315	-0.099319
H	-4.319548	-1.880940	1.256027
H	-5.252648	-0.393238	1.024474
H	-4.923149	-0.290775	-2.091755
H	-4.786767	1.105076	-1.016261
H	-3.463493	0.710544	-2.124835
H	-3.602827	-2.391379	-1.682345
H	-2.111366	-1.444911	-1.784253
H	-2.409409	-2.477606	-0.379299
C	0.853750	-2.021870	-0.752471
H	3.551828	-2.465634	-1.155111
O	2.778380	-2.160254	0.789382
C	0.587384	-3.316075	-0.623894
H	0.206167	-1.381661	-1.343769
H	-0.266192	-3.760469	-1.122401
H	1.211298	-3.960361	-0.013390

Conf. 2b

C	-1.036131	-1.082044	-0.571459
C	-2.142071	-2.048627	-0.127784
N	-2.665687	-1.142475	0.745311
C	-1.693715	-0.089339	0.422848
C	0.433134	-1.422499	-0.381862
O	1.181367	-0.306861	-0.836576
C	0.829094	-2.662983	-1.165361
C	-2.325125	1.172730	-0.121267
C	-3.798176	1.215019	-0.254129
C	-4.682282	0.038985	0.086982
C	-4.043068	-0.897457	1.117823
O	-2.498693	-3.163997	-0.439998
Si	2.241803	0.674622	0.021313
C	1.456593	1.230300	1.630200

C	3.866545	-0.250699	0.364997
C	2.520220	2.132897	-1.110662
C	4.883652	0.727512	0.978273
C	3.633104	-1.407171	1.351023
C	4.438035	-0.809056	-0.947782
H	-1.208226	-0.803632	-1.616084
H	-1.060063	0.163222	1.276883
H	0.615662	-1.595226	0.688176
H	1.886817	-2.885984	-1.011862
H	0.239380	-3.525096	-0.845169
H	0.660629	-2.501208	-2.233443
H	-4.884346	-0.507277	-0.839762
H	-5.640770	0.408527	0.462669
H	-4.566531	-1.852780	1.157044
H	-4.066426	-0.453303	2.114561
H	2.167026	1.829838	2.207663
H	0.579974	1.853694	1.430819
H	1.149436	0.389618	2.259146
H	3.194875	2.861193	-0.651232
H	2.955129	1.821394	-2.064082
H	1.570680	2.635148	-1.314382
H	5.823920	0.204493	1.193319
H	5.115928	1.552511	0.297756
H	4.524920	1.156381	1.919461
H	4.579572	-1.923223	1.555868
H	3.237396	-1.052537	2.307915
H	2.936867	-2.153003	0.955135
H	5.375573	-1.345500	-0.754115
H	3.746077	-1.507874	-1.426373
H	4.658745	-0.012835	-1.666014
C	-1.440329	2.035069	-0.951082
H	-4.199556	1.908318	-0.990270
O	-3.136628	1.858559	0.836213
C	-1.390998	3.357672	-0.845725
H	-0.796835	1.512552	-1.653038
H	-0.728068	3.943910	-1.471604
H	-2.012487	3.887828	-0.131845

Conf. 2c

C	-1.132488	-1.174621	-0.549352
C	-2.391270	-1.889440	-0.045646
N	-2.698877	-0.872936	0.809412
C	-1.578495	-0.020259	0.383693
C	0.227394	-1.788377	-0.267345
O	1.186087	-0.787430	-0.555676
C	0.468309	-3.046861	-1.086233
C	-2.026373	1.282056	-0.246873
C	-3.479185	1.556501	-0.328199

C	-4.526935	0.579997	0.149818
C	-3.992962	-0.373607	1.223019
O	-2.964164	-2.926076	-0.303295
Si	2.719067	-0.644715	0.121671
C	3.975271	-1.413331	-1.034920
C	3.021566	1.215432	0.318663
C	2.747557	-1.526028	1.775299
C	4.427347	1.448563	0.896844
C	2.912703	1.911891	-1.047047
C	1.975067	1.811532	1.272921
H	-1.228819	-0.937833	-1.612909
H	-0.873389	0.186738	1.194284
H	0.269392	-2.041611	0.801484
H	1.437501	-3.485930	-0.837542
H	-0.307917	-3.788817	-0.882665
H	0.457316	-2.811293	-2.153940
H	-4.871307	0.008081	-0.717678
H	-5.386937	1.134979	0.535163
H	-4.665419	-1.220122	1.362820
H	-3.882518	0.136576	2.181496
H	4.988487	-1.305515	-0.635707
H	3.779218	-2.481537	-1.166023
H	3.947048	-0.940670	-2.020669
H	3.723310	-1.392231	2.252078
H	1.984411	-1.141065	2.457732
H	2.585741	-2.601550	1.655268
H	4.604883	2.523312	1.027988
H	4.551482	0.974792	1.876153
H	5.210071	1.065719	0.234151
H	3.066206	2.992455	-0.931024
H	3.666588	1.546023	-1.751487
H	1.926474	1.762913	-1.494599
H	2.147845	2.888122	1.397894
H	0.963145	1.684389	0.879842
H	2.019786	1.353021	2.265987
C	-1.064228	1.918827	-1.187465
H	-3.797743	2.242499	-1.110352
O	-2.668192	2.170212	0.674033
C	-0.872310	3.229627	-1.273214
H	-0.489220	1.237870	-1.807318
H	-0.154402	3.642352	-1.972354
H	-1.421679	3.919827	-0.641632

Conf. 2d

C	1.012259	1.199310	-0.549510
C	2.138512	1.962509	0.157857
N	2.457065	0.890188	0.936816
C	1.480502	-0.008338	0.303178

C	-0.417275	1.684177	-0.372839
O	-1.265274	0.674513	-0.892638
C	-0.634609	3.008129	-1.091014
C	2.125514	-1.180030	-0.408627
C	3.600787	-1.301800	-0.357739
C	4.486176	-0.291415	0.331222
C	3.749007	0.469382	1.436981
O	2.629324	3.066928	0.069213
Si	-2.816167	0.272861	-0.372619
C	-3.628596	-0.487182	-1.872488
C	-2.723590	-0.977509	1.056426
C	-3.724356	1.815875	0.183747
C	-4.143914	-1.466013	1.392109
C	-1.869185	-2.186234	0.644700
C	-2.118053	-0.325181	2.309972
H	1.236963	1.087955	-1.614255
H	0.730973	-0.377219	1.007986
H	-0.605595	1.821740	0.701496
H	-1.649775	3.373643	-0.928498
H	0.069473	3.758971	-0.723933
H	-0.477627	2.877944	-2.165281
H	4.847539	0.410888	-0.426579
H	5.360438	-0.802041	0.744781
H	4.307427	1.353600	1.744626
H	3.597613	-0.161855	2.314217
H	-4.665020	-0.764262	-1.659072
H	-3.634545	0.228584	-2.699353
H	-3.098493	-1.384760	-2.202917
H	-4.688626	1.550460	0.627359
H	-3.157360	2.374486	0.934471
H	-3.917865	2.482326	-0.661386
H	-4.104131	-2.194480	2.211663
H	-4.796722	-0.648179	1.713372
H	-4.616750	-1.960224	0.537750
H	-1.818384	-2.911461	1.466695
H	-2.290628	-2.702718	-0.223641
H	-0.846073	-1.899062	0.390034
H	-2.065937	-1.055635	3.127078
H	-1.101616	0.041195	2.137642
H	-2.720452	0.519014	2.659633
C	1.337132	-1.798199	-1.510473
H	4.065898	-1.857990	-1.169115
O	2.766379	-2.101385	0.479968
C	1.309650	-3.103011	-1.754895
H	0.751076	-1.113903	-2.116522
H	0.720196	-3.505095	-2.570514
H	1.871699	-3.800564	-1.142771

Conf. 2e

C	1.133608	0.996358	-0.612054
C	2.430168	1.770556	-0.342943
N	2.796872	0.923999	0.660944
C	1.650016	0.023644	0.478752
C	-0.200187	1.679713	-0.359426
O	-1.208954	0.691911	-0.484171
C	-0.454262	2.813546	-1.339506
C	2.057171	-1.373185	0.057024
C	3.488593	-1.695339	-0.040913
C	4.570541	-0.664570	0.178712
C	4.114003	0.490518	1.076163
O	2.986253	2.732483	-0.827292
Si	-2.440007	0.426187	0.627514
C	-3.304533	2.046511	0.999033
C	-3.589198	-0.816716	-0.218464
C	-1.714650	-0.263007	2.212481
C	-4.731618	-1.187952	0.742173
C	-4.180988	-0.196799	-1.494340
C	-2.807745	-2.086166	-0.592395
H	1.142013	0.554782	-1.612724
H	1.011645	-0.035586	1.365113
H	-0.187817	2.086581	0.663234
H	-1.415883	3.285535	-1.126353
H	0.331736	3.569314	-1.268212
H	-0.475958	2.425560	-2.361581
H	4.864214	-0.277495	-0.802315
H	5.449319	-1.149367	0.612750
H	4.798583	1.335764	1.003066
H	4.067387	0.179329	2.121424
H	-4.096480	1.900914	1.739997
H	-2.599875	2.776209	1.410553
H	-3.752335	2.479327	0.100475
H	-2.501349	-0.427216	2.955217
H	-1.202636	-1.214991	2.046663
H	-0.996618	0.438658	2.648672
H	-5.413556	-1.897546	0.257456
H	-4.358469	-1.662833	1.655146
H	-5.321695	-0.312713	1.033378
H	-4.836577	-0.920294	-1.995156
H	-4.780485	0.691956	-1.273668
H	-3.398153	0.091069	-2.202213
H	-3.476078	-2.811602	-1.073084
H	-1.996457	-1.863733	-1.291173
H	-2.375966	-2.573317	0.288247
C	1.015446	-2.146976	-0.670383
H	3.770230	-2.524501	-0.685832
O	2.731373	-2.093683	1.107693

C	1.151703	-2.610478	-1.906154
H	0.082479	-2.290284	-0.130480
H	0.347970	-3.151655	-2.393259
H	2.065014	-2.460111	-2.473554

Conf. 2f

C	-1.009053	1.131645	0.482265
C	-2.108204	1.874348	-0.288281
N	-2.380430	0.786311	-1.063936
C	-1.449118	-0.098069	-0.349762
C	0.422192	1.618112	0.315473
O	1.269751	0.623552	0.863878
C	0.622555	2.956920	1.011064
C	-2.156183	-1.227261	0.376067
C	-3.611183	-1.373187	0.209392
C	-4.445666	-0.397338	-0.584942
C	-3.631685	0.349513	-1.645770
O	-2.607762	2.977618	-0.245817
Si	2.836426	0.231479	0.389732
C	3.618281	-0.502616	1.918439
C	2.794175	-1.036681	-1.026667
C	3.747161	1.774150	-0.162885
C	4.227976	-1.515553	-1.316382
C	1.940433	-2.250586	-0.629283
C	2.215759	-0.404934	-2.303536
H	-1.252346	1.040085	1.544351
H	-0.671358	-0.508356	-0.999220
H	0.623712	1.736431	-0.759006
H	1.636263	3.327962	0.852557
H	-0.083424	3.695373	0.622872
H	0.455130	2.844916	2.085789
H	-4.878316	0.318322	0.121695
H	-5.276209	-0.930187	-1.056102
H	-4.170800	1.224188	-2.009906
H	-3.413014	-0.295875	-2.498517
H	4.663681	-0.766998	1.734697
H	3.593176	0.221732	2.737395
H	3.092532	-1.404087	2.245097
H	4.729890	1.511243	-0.565670
H	3.202318	2.311405	-0.944931
H	3.902485	2.458927	0.675411
H	4.218691	-2.247958	-2.133237
H	4.883810	-0.694082	-1.621538
H	4.678665	-2.002120	-0.445789
H	1.941570	-2.994867	-1.435257
H	2.321922	-2.740155	0.272288
H	0.901175	-1.969733	-0.442437
H	2.192818	-1.144935	-3.113282

H	1.191037	-0.048031	-2.161067
H	2.818211	0.441709	-2.646890
C	-1.448111	-1.788883	1.559767
H	-4.148460	-1.902566	0.992823
O	-2.706549	-2.205241	-0.526223
C	-1.917958	-1.732772	2.799669
H	-0.479659	-2.241402	1.359714
H	-1.358276	-2.144091	3.632209
H	-2.873934	-1.270008	3.024798

Conf. 3a

C	-1.152316	-0.987237	-0.688594
C	-2.435277	-1.760740	-0.335664
N	-2.727603	-0.902772	0.677875
C	-1.615931	0.002084	0.410341
C	0.181414	-1.677088	-0.456084
O	1.190135	-0.683580	-0.544219
C	0.435479	-2.780170	-1.470488
C	-2.084195	1.365457	-0.043993
C	-3.512315	1.705275	0.170786
C	-4.531098	0.755421	0.770102
C	-3.932560	-0.526153	1.376503
O	-3.009384	-2.739203	-0.763791
Si	2.429004	-0.496845	0.576624
C	3.308113	-2.135602	0.807583
C	3.564222	0.821791	-0.165186
C	1.711636	0.051769	2.219819
C	4.709645	1.111523	0.819493
C	4.150305	0.315224	-1.492720
C	2.775076	2.115046	-0.422938
H	-1.194419	-0.563036	-1.694909
H	-0.932990	0.104526	1.257713
H	0.167842	-2.115317	0.553615
H	1.392838	-3.265895	-1.269527
H	-0.355985	-3.532433	-1.425851
H	0.463225	-2.359433	-2.479307
H	-5.235133	0.499401	-0.026623
H	-5.097226	1.284335	1.539454
H	-4.640780	-1.353121	1.326886
H	-3.678558	-0.364575	2.427489
H	4.110834	-2.043846	1.545604
H	2.613562	-2.899567	1.171247
H	3.744541	-2.494078	-0.128649
H	2.499465	0.124514	2.975724
H	1.227455	1.029358	2.144253
H	0.973471	-0.668551	2.586505
H	5.390971	1.859179	0.394571
H	4.337969	1.508796	1.769490

H	5.300285	0.215220	1.035596
H	4.802630	1.080451	-1.932078
H	4.751948	-0.588328	-1.352783
H	3.364070	0.088234	-2.218747
H	3.435333	2.877441	-0.855674
H	1.947731	1.950911	-1.118559
H	2.358005	2.529634	0.500772
C	-1.031167	2.412399	-0.098635
H	-3.747106	2.763856	0.252002
O	-3.010090	1.343274	-1.121020
C	-0.912664	3.295024	-1.082689
H	-0.323292	2.408295	0.725920
H	-0.118646	4.032510	-1.078773
H	-1.606377	3.298685	-1.916810

Conf. 3b

C	1.130325	1.013095	-0.689779
C	2.426423	1.782328	-0.403970
N	2.793014	0.921188	0.582613
C	1.654124	0.019043	0.374666
C	-0.199618	1.692064	-0.411310
O	-1.207192	0.698966	-0.512286
C	-0.477722	2.824966	-1.385835
C	2.089003	-1.349941	-0.095388
C	3.398519	-1.801391	0.398114
C	4.199221	-0.937887	1.344907
C	4.128388	0.560056	1.032741
O	2.988597	2.749043	-0.873453
Si	-2.426148	0.464651	0.620207
C	-3.311444	2.087711	0.925805
C	-3.565235	-0.831687	-0.155048
C	-1.676025	-0.139607	2.228754
C	-4.705276	-1.153812	0.825780
C	-4.158920	-0.285342	-1.463311
C	-2.776826	-2.115525	-0.458515
H	1.134742	0.590440	-1.698786
H	1.014637	-0.084808	1.255905
H	-0.167286	2.098807	0.611069
H	-1.433894	3.297715	-1.151131
H	0.309906	3.580604	-1.333240
H	-0.523057	2.436052	-2.406756
H	5.246500	-1.247369	1.337352
H	3.818784	-1.135068	2.352422
H	4.828749	0.815019	0.232961
H	4.407358	1.136431	1.917661
H	-4.098220	1.964540	1.676270
H	-2.614607	2.843838	1.301228
H	-3.769298	2.474838	0.011450

H	-2.450974	-0.256158	2.992552
H	-1.174961	-1.104282	2.108731
H	-0.945194	0.579124	2.612887
H	-5.384658	-1.892692	0.382891
H	-4.328574	-1.575188	1.763345
H	-5.299098	-0.266450	1.068347
H	-4.815673	-1.036107	-1.920610
H	-4.757631	0.614909	-1.292989
H	-3.376780	-0.038725	-2.187263
H	-3.439195	-2.865141	-0.909637
H	-1.957090	-1.927288	-1.157321
H	-2.353620	-2.558437	0.449210
C	0.992697	-2.295142	-0.419405
H	3.556286	-2.873403	0.485793
O	3.230834	-1.351339	-0.951738
C	0.982405	-3.094058	-1.479003
H	0.155590	-2.286459	0.273366
H	0.148945	-3.761291	-1.666117
H	1.805131	-3.099471	-2.186173

Conf. 3c

C	-1.132386	-1.056667	-0.578611
C	-2.363578	-1.828319	-0.073648
N	-2.637983	-0.874087	0.855742
C	-1.587980	0.039937	0.417566
C	0.230385	-1.675567	-0.317164
O	1.185428	-0.651027	-0.519067
C	0.487754	-2.873228	-1.219773
C	-2.154627	1.312149	-0.174930
C	-3.590302	1.600987	0.061686
C	-4.526675	0.688763	0.829077
C	-3.828276	-0.468563	1.563869
O	-2.918559	-2.868089	-0.358685
Si	2.770319	-0.625222	0.044027
C	3.915764	-1.273033	-1.287994
C	3.129098	1.194861	0.429914
C	2.894543	-1.690843	1.580033
C	4.621118	1.376243	0.753640
C	2.762979	2.066930	-0.781923
C	2.292041	1.633368	1.641806
H	-1.230184	-0.761582	-1.626201
H	-0.864726	0.275657	1.203253
H	0.262472	-2.002907	0.732386
H	1.447444	-3.338103	-0.980988
H	-0.297376	-3.622671	-1.091898
H	0.505392	-2.558331	-2.266808
H	-5.249200	0.294619	0.108709
H	-5.088041	1.285782	1.550517

H	-4.489185	-1.329948	1.660013
H	-3.533645	-0.157260	2.569539
H	4.956783	-1.244825	-0.951723
H	3.673082	-2.310112	-1.537323
H	3.838739	-0.678290	-2.202695
H	3.891945	-1.601435	2.021427
H	2.164969	-1.390296	2.337405
H	2.729393	-2.747484	1.349107
H	4.824292	2.423102	1.011880
H	4.934979	0.761765	1.604272
H	5.256233	1.119630	-0.099970
H	2.974220	3.121833	-0.564127
H	3.341991	1.792585	-1.670029
H	1.701880	1.981938	-1.030085
H	2.430592	2.705141	1.831471
H	1.224812	1.459486	1.475702
H	2.580756	1.096390	2.550645
C	-1.175457	2.405898	-0.406083
H	-3.882693	2.647321	0.015828
O	-3.120436	1.100933	-1.195666
C	-1.127325	3.135908	-1.513606
H	-0.464236	2.576998	0.397487
H	-0.386839	3.918069	-1.634293
H	-1.826360	2.967156	-2.325893

Conf. 3d

C	-1.138902	-1.118889	-0.586203
C	-2.449180	-1.818376	-0.183020
N	-2.777055	-0.813138	0.671094
C	-1.631820	0.025481	0.337010
C	0.181296	-1.770202	-0.210125
O	1.203123	-0.821800	-0.475603
C	0.438586	-3.048021	-0.990996
C	-2.047539	1.322587	-0.318754
C	-3.469163	1.708281	-0.250189
C	-4.540598	0.872350	0.424306
C	-4.007230	-0.318453	1.240547
O	-3.018886	-2.844817	-0.487170
Si	2.398128	-0.372219	0.617124
C	3.247018	-1.907937	1.273611
C	3.579142	0.719862	-0.380939
C	1.628278	0.573926	2.040066
C	4.645712	1.303409	0.561128
C	4.268380	-0.112899	-1.473574
C	2.802542	1.869872	-1.041136
H	-1.139137	-0.849278	-1.645425
H	-0.989666	0.234941	1.195738
H	0.152954	-2.007736	0.864236

H	1.390255	-3.488567	-0.685785
H	-0.357559	-3.775398	-0.813669
H	0.482608	-2.831190	-2.061787
H	-5.210410	0.516595	-0.363470
H	-5.131861	1.516876	1.077971
H	-4.732168	-1.131816	1.270213
H	-3.804062	-0.012690	2.270399
H	4.036802	-1.639870	1.982084
H	2.535540	-2.550495	1.801730
H	3.696953	-2.492138	0.466277
H	2.397352	0.885937	2.753346
H	1.106311	1.468547	1.687592
H	0.912393	-0.049293	2.585365
H	5.354687	1.915280	-0.010243
H	4.203256	1.944972	1.329551
H	5.220647	0.519394	1.065080
H	4.928197	0.526174	-2.073939
H	4.882593	-0.912547	-1.048181
H	3.540265	-0.569456	-2.150865
H	3.492227	2.524378	-1.588978
H	2.064709	1.491653	-1.754344
H	2.276842	2.486385	-0.304567
C	-0.956324	2.320890	-0.484951
H	-3.697605	2.766642	-0.345077
O	-2.907720	1.149839	-1.447893
C	-0.894830	3.476621	0.163699
H	-0.158904	2.022548	-1.161411
H	-0.060440	4.155299	0.024527
H	-1.671538	3.781101	0.858689

Conf. 3e

C	1.141335	1.124947	-0.610587
C	2.456027	1.835635	-0.265085
N	2.846174	0.839683	0.574320
C	1.685017	-0.011671	0.289388
C	-0.175240	1.761474	-0.199361
O	-1.195245	0.808242	-0.449188
C	-0.461074	3.043841	-0.962920
C	2.078666	-1.313893	-0.367320
C	3.393854	-1.839687	-0.003948
C	4.244390	-1.124597	1.022249
C	4.189430	0.403260	0.920992
O	3.013945	2.854683	-0.613873
Si	-2.373237	0.350593	0.658072
C	-3.184851	1.881651	1.371041
C	-3.596956	-0.690754	-0.342742
C	-1.581861	-0.644730	2.034449
C	-4.772509	-1.100360	0.560715

C	-4.131213	0.130889	-1.526610
C	-2.904464	-1.954287	-0.876608
H	1.111980	0.853716	-1.670022
H	1.075529	-0.224055	1.171624
H	-0.121979	1.989421	0.876085
H	-1.408052	3.474269	-0.629754
H	0.333556	3.776245	-0.800392
H	-0.531428	2.835066	-2.033911
H	5.285118	-1.442205	0.931510
H	3.897600	-1.454258	2.007089
H	4.865712	0.754727	0.137115
H	4.510438	0.850284	1.864501
H	-3.926727	1.607061	2.126982
H	-2.445309	2.527595	1.854510
H	-3.688939	2.465445	0.595775
H	-2.340898	-1.016110	2.730128
H	-1.035807	-1.503268	1.632710
H	-0.883074	-0.030775	2.611456
H	-5.486472	-1.709242	-0.007636
H	-4.441520	-1.696465	1.417340
H	-5.314135	-0.229624	0.943899
H	-4.838142	-0.469969	-2.112759
H	-4.660563	1.028790	-1.192212
H	-3.323408	0.444290	-2.194018
H	-3.614510	-2.556622	-1.457413
H	-2.066002	-1.703730	-1.532506
H	-2.522068	-2.583142	-0.066662
C	0.947931	-2.175684	-0.802206
H	3.552260	-2.912485	-0.076268
O	3.188854	-1.199984	-1.273589
C	0.653009	-3.345611	-0.249925
H	0.322237	-1.763312	-1.590027
H	-0.204934	-3.920826	-0.579857
H	1.252259	-3.762961	0.553539

Conf. 3f

C	1.032794	1.037611	-0.642449
C	2.096995	1.887982	0.071734
N	2.347120	0.901639	0.975255
C	1.478063	-0.081255	0.334201
C	-0.408773	1.513308	-0.554453
O	-1.227028	0.424679	-0.948652
C	-0.628880	2.729247	-1.443581
C	2.265521	-1.225762	-0.270551
C	3.693526	-1.359283	0.109775
C	4.415205	-0.407391	1.043356
C	3.488105	0.563075	1.792615
O	2.572251	2.993530	-0.074258

Si	-2.813479	0.091358	-0.500680
C	-3.389998	-1.131811	-1.788807
C	-2.875754	-0.684085	1.236279
C	-3.849599	1.652628	-0.540230
C	-4.345135	-0.948081	1.611469
C	-2.112827	-2.016990	1.245440
C	-2.259572	0.253649	2.287489
H	1.301109	0.831394	-1.681489
H	0.697515	-0.462897	0.997162
H	-0.623432	1.787071	0.487624
H	-1.643880	3.113810	-1.335560
H	0.072510	3.522685	-1.173236
H	-0.464838	2.461108	-2.490879
H	5.132894	0.153615	0.438080
H	4.989768	-0.988426	1.767325
H	4.011008	1.480414	2.062890
H	3.117241	0.103270	2.712261
H	-4.389439	-1.511239	-1.557276
H	-3.428795	-0.657695	-2.773514
H	-2.708401	-1.984785	-1.848990
H	-4.893512	1.418887	-0.311160
H	-3.506267	2.393648	0.187625
H	-3.821529	2.110456	-1.532865
H	-4.397254	-1.442318	2.589632
H	-4.920869	-0.020291	1.682661
H	-4.844007	-1.601406	0.888453
H	-2.141950	-2.465033	2.246553
H	-2.544028	-2.740152	0.546575
H	-1.062501	-1.878586	0.975822
H	-2.373828	-0.179971	3.289050
H	-1.188918	0.406973	2.122847
H	-2.742666	1.235864	2.297829
C	1.470591	-2.411306	-0.685705
H	4.122810	-2.354485	0.023629
O	3.298275	-0.814660	-1.154945
C	1.597363	-3.012312	-1.862319
H	0.752271	-2.773809	0.044489
H	0.994422	-3.876252	-2.116671
H	2.309692	-2.656076	-2.598848

Conf. 4a

C	2.083937	0.500314	-0.553014
H	1.547936	0.332425	-1.493688
C	4.151455	-0.340898	-1.199642
C	4.178900	-1.394836	-0.092722
C	2.756908	-1.811955	0.301435
N	1.956313	-0.609355	0.380616
C	1.429384	0.141264	1.383263

C	1.393088	1.397519	0.496447
O	1.120561	-0.126077	2.524343
O	3.425759	0.823683	-0.794151
H	3.698300	-0.751266	-2.113516
H	5.158566	-0.001768	-1.438938
H	4.744214	-2.264514	-0.435662
H	4.693277	-0.978115	0.777830
H	2.325007	-2.478541	-0.452158
H	2.728638	-2.322077	1.264793
H	2.057932	2.180391	0.866762
C	0.016639	1.950181	0.153054
O	-0.761941	0.894003	-0.375044
C	0.095824	3.090911	-0.850989
Si	-2.287916	0.402039	0.122184
C	-2.413199	-1.392072	-0.469023
C	-2.409489	0.530966	1.986068
C	-3.583333	1.490125	-0.680458
H	-0.426348	2.323102	1.086310
H	-0.906882	3.458512	-1.079521
H	0.686929	3.918403	-0.450434
H	0.558197	2.748762	-1.781048
H	-3.316592	0.033127	2.342635
H	-1.546253	0.063702	2.467872
H	-2.458315	1.574372	2.311597
H	-4.590539	1.198244	-0.367442
H	-3.434694	2.535003	-0.390382
H	-3.535094	1.431472	-1.771487
C	-1.386704	-2.253133	0.284683
H	-0.367470	-1.889367	0.125358
H	-1.577049	-2.260798	1.362691
H	-1.435117	-3.291743	-0.066931
C	-2.122627	-1.466951	-1.976478
H	-2.193863	-2.506297	-2.322004
H	-2.837740	-0.877304	-2.559404
H	-1.116973	-1.104307	-2.207821
C	-3.827884	-1.929676	-0.198191
H	-4.590371	-1.363443	-0.742607
H	-3.900617	-2.975479	-0.522242
H	-4.080886	-1.898983	0.866772

Conf. 4b

C	-2.213505	0.686050	0.222044
H	-1.805489	1.065191	1.164705
C	-4.423182	0.407264	0.894871
C	-4.442857	-1.082048	0.553908
C	-3.041035	-1.689854	0.682260
N	-2.101143	-0.761452	0.089447
C	-1.416205	-0.713793	-1.084263

C	-1.328030	0.819705	-1.035361
O	-1.054001	-1.579987	-1.850158
O	-3.531739	1.124147	0.035891
H	-4.123414	0.561822	1.941406
H	-5.406397	0.853359	0.750513
H	-5.137766	-1.597790	1.220737
H	-4.803844	-1.203465	-0.471339
H	-2.779001	-1.838382	1.734340
H	-2.960162	-2.649768	0.171169
H	-1.868435	1.279168	-1.865331
C	0.047157	1.449317	-0.864172
O	0.645928	0.895861	0.293518
C	-0.054876	2.963928	-0.736976
Si	2.269482	0.545988	0.552931
C	2.659304	-1.194967	-0.094983
C	3.337024	1.823006	-0.309015
C	2.478770	0.634169	2.406519
H	0.637120	1.201791	-1.756951
H	0.936989	3.402769	-0.614649
H	-0.515099	3.394943	-1.629719
H	-0.661088	3.230997	0.133176
H	4.394213	1.551699	-0.232796
H	3.092595	1.909508	-1.371861
H	3.211480	2.807831	0.149742
H	3.510927	0.415611	2.695667
H	2.231764	1.636065	2.768960
H	1.826421	-0.081673	2.914252
C	2.488562	-1.243009	-1.622123
H	1.456916	-1.037614	-1.921253
H	3.146286	-0.528844	-2.128358
H	2.741081	-2.243841	-1.996073
C	1.709330	-2.214758	0.553727
H	1.916130	-3.220805	0.166589
H	1.834079	-2.249433	1.640896
H	0.662276	-1.984772	0.338835
C	4.110208	-1.562038	0.259965
H	4.279733	-1.562408	1.341456
H	4.341915	-2.569274	-0.108932
H	4.830546	-0.874357	-0.194762

Conf. 4c

C	2.248325	0.732836	-0.299052
H	1.747976	0.940762	-1.250797
C	4.449255	0.805352	-1.049471
C	4.758349	-0.616580	-0.583490
C	3.492302	-1.481321	-0.594451
N	2.413039	-0.691704	-0.038597
C	1.763965	-0.664084	1.156436

C	1.391352	0.817794	0.981964
O	1.590813	-1.509647	2.006655
O	3.465518	1.422095	-0.213966
H	4.094148	0.804016	-2.090073
H	5.335082	1.436526	-0.990438
H	5.517527	-1.055072	-1.235378
H	5.165909	-0.573788	0.430526
H	3.231311	-1.771026	-1.617019
H	3.608891	-2.388995	-0.001464
H	1.861403	1.442716	1.744039
C	-0.082326	1.169490	0.816561
O	-0.618975	0.395356	-0.239185
C	-0.256698	2.650584	0.510634
Si	-1.897641	-0.694283	-0.198012
C	-3.549099	0.242035	-0.277299
C	-1.642453	-1.745703	-1.719699
C	-1.810782	-1.728171	1.359366
H	-0.588253	0.933082	1.762326
H	-1.313965	2.894319	0.391785
H	0.150665	3.262003	1.319630
H	0.260088	2.909363	-0.417628
H	-2.397070	-2.535023	-1.782666
H	-1.700916	-1.147325	-2.633180
H	-0.657702	-2.220749	-1.686272
H	-2.642531	-2.439147	1.384905
H	-0.874779	-2.290849	1.396950
H	-1.869820	-1.116717	2.264133
C	-3.523874	1.249623	-1.437155
H	-2.728392	1.989795	-1.312770
H	-3.371352	0.754210	-2.401514
H	-4.478388	1.788532	-1.491032
C	-3.803999	0.989583	1.041936
H	-4.744052	1.552491	0.981191
H	-3.886866	0.300910	1.888173
H	-3.010082	1.707207	1.271757
C	-4.691988	-0.761702	-0.507821
H	-4.747143	-1.510921	0.288853
H	-5.654904	-0.235851	-0.530002
H	-4.583901	-1.289424	-1.460485

Conf. 4d

C	1.820849	-0.479450	0.034648
H	1.185478	-0.850034	0.847307
C	3.125998	-2.381412	-0.262184
C	4.437898	-1.640964	-0.007626
C	4.218007	-0.446829	0.928593
N	3.006386	0.226975	0.507613
C	2.738150	1.368636	-0.184397

C	1.381397	0.820499	-0.665031
O	3.365618	2.393773	-0.332772
O	2.147804	-1.515715	-0.846525
H	2.728194	-2.800733	0.673110
H	3.268802	-3.196714	-0.970156
H	5.164870	-2.329926	0.428563
H	4.835154	-1.290085	-0.964277
H	4.096868	-0.783344	1.962735
H	5.046704	0.261038	0.894364
H	1.338732	0.705997	-1.750726
C	0.121453	1.494704	-0.144850
O	-0.970499	0.653548	-0.473814
C	-0.071313	2.877012	-0.747658
Si	-2.189822	0.212064	0.596027
C	-3.363304	-0.853330	-0.435894
C	-1.451698	-0.754375	2.022151
C	-3.024705	1.752219	1.257976
H	0.210269	1.591586	0.948033
H	-0.974989	3.340636	-0.346440
H	0.783554	3.518355	-0.519190
H	-0.171236	2.800467	-1.833791
H	-2.226936	-1.008153	2.751667
H	-0.989752	-1.685619	1.682527
H	-0.690780	-0.166998	2.545617
H	-3.832555	1.490261	1.948037
H	-2.309958	2.373015	1.807611
H	-3.447269	2.357001	0.451009
C	-2.600382	-2.053234	-1.020177
H	-1.777411	-1.730678	-1.664292
H	-2.184222	-2.692278	-0.234864
H	-3.277650	-2.671487	-1.622914
C	-3.954053	-0.020501	-1.584799
H	-4.623443	-0.641329	-2.193666
H	-4.539203	0.826613	-1.213112
H	-3.171596	0.371162	-2.241408
C	-4.504798	-1.364827	0.459080
H	-5.084590	-0.543081	0.891988
H	-5.196235	-1.979976	-0.130145
H	-4.132365	-1.985150	1.280646

Conf. 4e

C	1.979460	-0.459610	-0.032129
H	1.351596	-1.032897	0.658317
C	3.693188	-2.011944	-0.284156
C	4.772043	-1.046286	0.203271
C	4.186973	-0.023842	1.184676
N	2.917368	0.424665	0.649884
C	2.489094	1.551112	0.017034

C	1.353785	0.785148	-0.690112
O	2.889440	2.693875	0.035752
O	2.626566	-1.312425	-0.933030
H	3.287717	-2.597245	0.553602
H	4.093909	-2.703932	-1.023658
H	5.573726	-1.611033	0.684725
H	5.195446	-0.526407	-0.660734
H	4.018366	-0.480866	2.164523
H	4.839908	0.839233	1.317825
H	1.478517	0.775158	-1.775518
C	-0.080319	1.141551	-0.323992
O	-0.930553	0.175490	-0.917448
C	-0.438691	2.534087	-0.818713
Si	-2.053803	-0.819596	-0.158044
C	-3.568470	0.180279	0.407032
C	-2.518553	-2.062911	-1.469863
C	-1.264877	-1.669479	1.315429
H	-0.177510	1.111680	0.771071
H	-1.458166	2.794841	-0.529364
H	0.243620	3.276882	-0.398683
H	-0.368315	2.571511	-1.909169
H	-3.206180	-2.813219	-1.069010
H	-3.002274	-1.581865	-2.324351
H	-1.626270	-2.581980	-1.831077
H	-2.019883	-2.228646	1.876493
H	-0.501189	-2.381409	0.989513
H	-0.801718	-0.959654	2.007043
C	-4.123987	1.004957	-0.764718
H	-3.385474	1.711794	-1.152749
H	-4.441793	0.365686	-1.594563
H	-4.999382	1.581031	-0.439119
C	-3.197524	1.120878	1.565587
H	-4.081648	1.683646	1.890821
H	-2.822321	0.568513	2.432757
H	-2.436134	1.852464	1.279120
C	-4.658899	-0.792020	0.891552
H	-4.320143	-1.403963	1.733680
H	-5.537656	-0.228661	1.229707
H	-4.988046	-1.466014	0.094648

Conf. 4f

C	1.698993	-0.392504	-0.187368
H	0.980711	-1.001858	0.372202
C	3.123186	-2.105794	-0.848796
C	4.347963	-1.473738	-0.189489
C	3.941032	-0.624858	1.020789
N	2.767765	0.139524	0.648767
C	2.534043	1.438654	0.315523

C	1.292243	1.056495	-0.513795
O	3.116680	2.463902	0.590568
O	2.195571	-1.105894	-1.282789
H	2.618265	-2.793104	-0.154820
H	3.404314	-2.664143	-1.740659
H	5.039267	-2.260573	0.120909
H	4.858044	-0.845274	-0.924961
H	3.694033	-1.262559	1.875017
H	4.730170	0.063407	1.324927
H	1.421822	1.282611	-1.574916
C	-0.065155	1.541017	-0.028355
O	-1.045973	0.858394	-0.790762
C	-0.201787	3.047862	-0.191103
Si	-2.573382	0.359778	-0.291000
C	-2.456139	-1.347236	0.536786
C	-3.295466	1.611677	0.901953
C	-3.575056	0.267507	-1.863624
H	-0.167037	1.285364	1.036279
H	-1.171748	3.385465	0.178158
H	0.582658	3.565353	0.366514
H	-0.116618	3.318156	-1.247165
H	-4.244886	1.248420	1.306715
H	-2.627688	1.805232	1.746621
H	-3.489608	2.562171	0.397155
H	-4.603559	-0.041440	-1.655578
H	-3.608354	1.246707	-2.349496
H	-3.142782	-0.447637	-2.568926
C	-1.697348	-1.250235	1.870498
H	-0.675251	-0.881756	1.742738
H	-2.203331	-0.585272	2.577278
H	-1.629002	-2.240113	2.339028
C	-1.733945	-2.331453	-0.397454
H	-1.635246	-3.310771	0.087641
H	-2.287754	-2.480945	-1.329719
H	-0.730517	-1.986417	-0.661868
C	-3.873853	-1.876373	0.816433
H	-4.453395	-1.996979	-0.103995
H	-3.817337	-2.859568	1.300360
H	-4.434677	-1.215384	1.485065

Conf. 5a

C	2.932683	-0.038820	-1.573887
C	2.179748	1.260400	-1.925266
N	2.021116	1.577425	-0.609656
C	2.838696	0.472651	-0.118258
C	1.838390	2.810141	0.131296
C	3.178606	3.165804	0.784564
C	3.832048	1.927308	1.395154

O	4.057149	0.918021	0.403979
O	1.832740	1.809619	-2.945008
C	2.203746	-1.336705	-1.873085
C	2.892926	-2.549597	-1.276518
O	0.902754	-1.125731	-1.277682
H	2.311822	-0.159308	0.603773
C	-0.142873	-1.887587	-1.562455
O	-0.124151	-2.853490	-2.282958
C	-1.366954	-1.391397	-0.807555
C	-1.443259	-2.056694	0.593456
C	-0.320608	-1.675688	1.525711
C	0.776127	-2.519121	1.704944
C	1.823734	-2.161110	2.546824
C	1.791089	-0.945948	3.222788
C	0.702859	-0.095810	3.053392
C	-0.344948	-0.461164	2.215319
N	-1.343330	0.060325	-0.756192
O	-3.530413	0.116871	-0.379862
C	-4.811620	0.678379	0.060410
C	-5.257572	1.781859	-0.892086
C	-5.745262	-0.522299	-0.029531
C	-4.695519	1.160543	1.502588
C	-2.405666	0.835138	-0.406950
O	-2.308920	2.025752	-0.170230
H	3.954317	-0.059392	-1.953963
H	1.491690	3.586269	-0.551364
H	1.067056	2.651473	0.891186
H	3.026717	3.916156	1.563815
H	3.853163	3.588031	0.034386
H	3.205093	1.512654	2.196455
H	4.810346	2.165793	1.810158
H	2.066733	-1.454112	-2.950663
H	2.309570	-3.452465	-1.457153
H	3.872454	-2.670523	-1.743918
H	3.031957	-2.424501	-0.200695
H	-2.237207	-1.713286	-1.377774
H	-1.470302	-3.140208	0.451543
H	-2.404735	-1.762002	1.020174
H	0.811856	-3.467778	1.178113
H	2.666730	-2.831435	2.672888
H	2.607989	-0.663833	3.877497
H	0.666435	0.852665	3.577833
H	-1.195871	0.203075	2.105516
H	-0.442442	0.505992	-0.654236
H	-6.272620	2.090487	-0.629893
H	-4.600500	2.648259	-0.829541
H	-5.267497	1.413301	-1.920945
H	-6.751132	-0.230675	0.280430

H	-5.399205	-1.326797	0.623672
H	-5.792161	-0.896835	-1.054633
H	-4.042833	2.029274	1.580622
H	-5.688012	1.434418	1.868486
H	-4.305425	0.361036	2.138130

Conf. 5b

C	1.422249	-1.753645	-0.212949
C	2.634454	-2.428064	-0.886614
N	3.466646	-1.426756	-0.491894
C	2.457284	-0.739610	0.308264
C	4.887142	-1.342334	-0.217417
C	5.068503	-1.375329	1.304131
C	4.048186	-0.469495	1.990525
O	2.707206	-0.864582	1.679731
O	2.819024	-3.440709	-1.522436
C	0.343826	-1.237114	-1.142852
C	-0.557418	-2.346724	-1.650234
O	-0.392846	-0.274335	-0.350408
H	2.319405	0.309824	0.029941
C	-1.127662	0.627529	-0.990890
O	-1.302825	0.648060	-2.184951
C	-1.690383	1.677223	-0.032937
C	-0.974570	3.015739	-0.274170
C	0.524796	2.936304	-0.115810
C	1.106543	2.949355	1.153055
C	2.487715	2.907329	1.300416
C	3.308685	2.846945	0.177809
C	2.739353	2.815171	-1.089959
C	1.355728	2.858173	-1.233696
N	-3.112304	1.863063	-0.238414
O	-3.483732	-0.145580	0.610275
C	-4.205195	-1.389657	0.896470
C	-4.565858	-2.075718	-0.415948
C	-3.169056	-2.199260	1.666048
C	-5.424831	-1.113329	1.768563
C	-4.035989	0.877243	-0.043746
O	-5.191805	0.960401	-0.411918
H	0.999965	-2.357409	0.593111
H	5.393216	-2.171934	-0.711797
H	5.272395	-0.406904	-0.634029
H	6.077063	-1.049091	1.568413
H	4.936711	-2.398264	1.668014
H	4.196603	0.576268	1.691490
H	4.135315	-0.533181	3.074348
H	0.790980	-0.694155	-1.980543
H	-1.323032	-1.951758	-2.317424
H	0.045365	-3.073255	-2.199922

H	-1.036721	-2.861832	-0.814475
H	-1.510661	1.340952	0.988340
H	-1.389410	3.739417	0.431977
H	-1.217895	3.361166	-1.283377
H	0.472309	3.007608	2.032206
H	2.923996	2.924990	2.292907
H	4.386706	2.819939	0.291176
H	3.370977	2.761406	-1.969460
H	0.916535	2.843044	-2.226078
H	-3.409682	2.595131	-0.864805
H	-4.991800	-3.059102	-0.203068
H	-5.297102	-1.494675	-0.977908
H	-3.671356	-2.214240	-1.028484
H	-3.574440	-3.184504	1.906019
H	-2.902854	-1.695299	2.597986
H	-2.263774	-2.327923	1.069671
H	-6.189324	-0.562141	1.223137
H	-5.845569	-2.066125	2.099499
H	-5.135505	-0.542507	2.654594

Conf. 5c

C	-1.425893	-1.495782	-0.713731
C	-2.354404	-2.724220	-0.833336
N	-3.078518	-2.340063	0.252046
C	-2.383803	-1.068781	0.419057
C	-4.444218	-2.556443	0.687994
C	-5.240072	-1.279285	0.392598
C	-4.438594	-0.036926	0.776468
O	-3.197074	0.012093	0.067708
O	-2.443218	-3.672070	-1.579446
C	0.024564	-1.782857	-0.373441
C	0.764052	-2.465931	-1.505604
O	0.702447	-0.523047	-0.139066
H	-1.957687	-0.932870	1.418075
C	0.886109	-0.106459	1.107682
O	0.444192	-0.649790	2.092215
C	1.786995	1.125544	1.188367
C	3.161556	0.708779	1.758547
C	3.894843	-0.291004	0.896287
C	3.659972	-1.660245	1.042806
C	4.289608	-2.582470	0.216106
C	5.167414	-2.149375	-0.773237
C	5.419673	-0.790317	-0.920328
C	4.790740	0.130398	-0.087452
N	1.948092	1.826832	-0.062648
O	-0.116390	2.627879	0.107306
C	-1.302890	3.408732	-0.269925
C	-1.966859	2.797987	-1.498760

C	-2.198392	3.255097	0.953206
C	-0.913636	4.867877	-0.477490
C	0.943823	2.505230	-0.691282
O	1.048606	2.941035	-1.822477
H	-1.502177	-0.836812	-1.581716
H	-4.848966	-3.423423	0.165328
H	-4.442252	-2.772152	1.760477
H	-6.181769	-1.290013	0.945956
H	-5.474268	-1.230625	-0.674507
H	-4.241004	-0.017590	1.857655
H	-4.973162	0.873743	0.508881
H	0.086556	-2.371685	0.544131
H	1.807282	-2.626019	-1.228072
H	0.300428	-3.431178	-1.717622
H	0.727478	-1.855824	-2.411376
H	1.316432	1.793947	1.909830
H	2.991826	0.294054	2.753720
H	3.753159	1.619258	1.875539
H	2.977663	-2.007174	1.812666
H	4.095227	-3.641437	0.345042
H	5.657782	-2.868210	-1.419749
H	6.111250	-0.443841	-1.680013
H	5.009937	1.188540	-0.196686
H	2.669361	1.502876	-0.689863
H	-2.911322	3.317620	-1.681373
H	-1.337876	2.898244	-2.382195
H	-2.190186	1.744642	-1.318757
H	-3.130799	3.801954	0.794750
H	-1.707117	3.656646	1.842737
H	-2.436379	2.202147	1.115671
H	-0.281895	4.990634	-1.356739
H	-1.820940	5.461306	-0.614811
H	-0.385631	5.249764	0.400132

Conf. 5d

C	-1.037824	-2.068797	0.010756
C	-2.088350	-3.142374	0.361633
N	-3.090666	-2.223136	0.369619
C	-2.247671	-1.117740	-0.072696
C	-4.520008	-2.275498	0.136159
C	-4.781801	-1.711820	-1.265021
C	-3.957263	-0.448651	-1.503442
O	-2.555476	-0.709942	-1.372304
O	-2.074615	-4.336310	0.555230
C	0.016330	-1.777367	1.057738
C	1.015552	-2.907535	1.209870
O	0.675162	-0.574417	0.589817
H	-2.257042	-0.265970	0.617290

C	1.277378	0.206181	1.478617
O	1.330203	-0.016302	2.664536
C	1.934718	1.428706	0.835673
C	3.470839	1.267791	0.839206
C	3.962305	0.125734	-0.016359
C	4.074324	-1.159996	0.515364
C	4.472307	-2.227437	-0.280049
C	4.769729	-2.023614	-1.623959
C	4.675639	-0.745022	-2.162434
C	4.277856	0.321513	-1.362212
N	1.450743	1.721819	-0.492541
O	-0.327823	2.756465	0.352613
C	-1.544944	3.576252	0.331767
C	-2.728409	2.738754	-0.136401
C	-1.707737	3.963411	1.796263
C	-1.330309	4.815603	-0.530358
C	0.236699	2.284473	-0.761158
O	-0.228119	2.349099	-1.883354
H	-0.586420	-2.240764	-0.968770
H	-4.856634	-3.307713	0.235377
H	-5.026168	-1.674018	0.897142
H	-5.842541	-1.478913	-1.382370
H	-4.512889	-2.457621	-2.018283
H	-4.248157	0.342130	-0.798331
H	-4.097940	-0.072683	-2.515779
H	-0.452289	-1.547364	2.018089
H	1.747118	-2.673585	1.983662
H	0.487505	-3.820505	1.493137
H	1.538205	-3.082878	0.266795
H	1.690275	2.268322	1.485770
H	3.785007	1.125404	1.875076
H	3.893473	2.213035	0.492254
H	3.848434	-1.325541	1.564148
H	4.550716	-3.219584	0.150331
H	5.080298	-2.855576	-2.245756
H	4.915877	-0.574697	-3.205886
H	4.220716	1.319456	-1.786526
H	1.812967	1.164613	-1.252525
H	-3.636286	3.345611	-0.095299
H	-2.587850	2.385425	-1.157078
H	-2.866407	1.881094	0.525917
H	-2.611928	4.563564	1.919702
H	-0.851450	4.549771	2.137261
H	-1.794782	3.071428	2.420930
H	-1.254489	4.560644	-1.586524
H	-2.176826	5.492757	-0.393529
H	-0.421851	5.339240	-0.221819

Conf. 5e

C	2.612148	1.446114	-0.826665
C	4.113534	1.628109	-0.525585
N	4.256874	0.310580	-0.217293
C	2.894735	-0.050868	-0.598671
C	5.366049	-0.621303	-0.265705
C	5.204478	-1.470274	-1.531420
C	3.754403	-1.918989	-1.699357
O	2.866940	-0.798095	-1.781902
O	4.893556	2.552580	-0.542818
C	1.655544	2.136103	0.123938
C	1.522494	3.617981	-0.168800
O	0.391212	1.449096	-0.061824
H	2.337368	-0.555943	0.196611
C	-0.507664	1.521790	0.910984
O	-0.396112	2.196211	1.906364
C	-1.692954	0.608725	0.647970
C	-1.663028	-0.571427	1.643085
C	-0.403546	-1.398274	1.570825
C	-0.221077	-2.326598	0.543648
C	0.926214	-3.108950	0.490104
C	1.911905	-2.971454	1.463676
C	1.749786	-2.036822	2.479852
C	0.599930	-1.254329	2.529553
N	-2.902634	1.398873	0.782876
O	-3.986318	-0.001511	-0.554257
C	-5.131108	-0.629180	-1.221813
C	-5.812277	0.371226	-2.148656
C	-4.470245	-1.744195	-2.023155
C	-6.085027	-1.208087	-0.182812
C	-4.115690	1.030056	0.282006
O	-5.152512	1.600539	0.565483
H	2.364861	1.659617	-1.868621
H	6.300466	-0.059569	-0.261149
H	5.337910	-1.249929	0.629297
H	5.850869	-2.349342	-1.478081
H	5.499428	-0.885004	-2.407019
H	3.449764	-2.561534	-0.863420
H	3.621401	-2.474375	-2.626817
H	1.963132	1.973048	1.160536
H	0.848021	4.096474	0.540824
H	2.505631	4.087359	-0.088474
H	1.147224	3.771083	-1.183499
H	-1.627930	0.236299	-0.372353
H	-2.534155	-1.193167	1.421732
H	-1.790502	-0.167236	2.650755
H	-0.992302	-2.447876	-0.211167
H	1.049890	-3.829894	-0.310390

H	2.804053	-3.586700	1.427382
H	2.515737	-1.918218	3.237952
H	0.473401	-0.532809	3.330500
H	-2.940366	2.062873	1.542190
H	-6.569912	-0.149543	-2.739470
H	-6.295655	1.168732	-1.585738
H	-5.083781	0.808262	-2.836234
H	-5.229879	-2.300563	-2.576543
H	-3.947321	-2.435663	-1.358232
H	-3.752821	-1.331507	-2.736135
H	-6.578484	-0.422042	0.387399
H	-6.848025	-1.802231	-0.691581
H	-5.543460	-1.863447	0.504274

Conf. 5f

C	-3.083143	-1.350548	-0.582045
C	-4.503117	-1.687694	-0.074028
N	-4.573339	-0.529826	0.637433
C	-3.328298	0.011508	0.103465
C	-5.674199	0.305664	1.077430
C	-5.791046	1.482063	0.101979
C	-4.411611	2.044125	-0.232883
O	-3.571535	1.040949	-0.813084
O	-5.279632	-2.602301	-0.221324
C	-1.989628	-2.250935	-0.037057
C	-2.062014	-3.653830	-0.605687
O	-0.693420	-1.714265	-0.413228
H	-2.620122	0.332388	0.875649
C	0.047980	-1.113612	0.512374
O	-0.327421	-0.834616	1.624871
C	1.445938	-0.829420	-0.015714
C	1.416528	0.180138	-1.191592
C	0.935170	1.546561	-0.781678
C	1.823147	2.487591	-0.256840
C	1.371614	3.731592	0.169343
C	0.020995	4.053686	0.074661
C	-0.870649	3.126213	-0.453557
C	-0.414759	1.883266	-0.878830
N	2.250176	-0.393618	1.103171
O	4.121287	-0.788550	-0.015185
C	5.547579	-0.686449	-0.337347
C	6.377551	-1.464906	0.677367
C	5.622593	-1.342680	-1.710491
C	5.951984	0.781477	-0.421886
C	3.601976	-0.286562	1.109779
O	4.230112	0.198001	2.034251
H	-3.029480	-1.266770	-1.669433
H	-6.582330	-0.296775	1.107785

H	-5.463105	0.660492	2.090245
H	-6.408625	2.270471	0.537917
H	-6.269788	1.148240	-0.822806
H	-3.926405	2.447006	0.667760
H	-4.482252	2.841378	-0.971921
H	-2.035035	-2.270610	1.053193
H	-1.264670	-4.271386	-0.189488
H	-3.023879	-4.103292	-0.352163
H	-1.964273	-3.632636	-1.693695
H	1.836514	-1.774465	-0.400509
H	0.782406	-0.234162	-1.977991
H	2.433682	0.235195	-1.582905
H	2.879428	2.247586	-0.186398
H	2.075476	4.450903	0.573160
H	-0.332413	5.024144	0.404727
H	-1.923017	3.369395	-0.542557
H	-1.121845	1.175259	-1.297647
H	1.768834	-0.012237	1.903166
H	7.415521	-1.499435	0.336955
H	6.345955	-0.995873	1.659744
H	6.010617	-2.491207	0.758841
H	6.652591	-1.321965	-2.073185
H	4.991258	-0.809817	-2.425414
H	5.293520	-2.383015	-1.657825
H	5.897464	1.266272	0.552367
H	6.979199	0.849224	-0.788144
H	5.304249	1.312564	-1.124631

Conf. 6a

C	1.106027	1.572823	0.625335
C	1.164845	0.332421	1.524324
N	1.860016	-0.367008	0.602591
C	1.914388	0.717341	-0.388249
O	0.757553	0.043511	2.634746
C	-0.284787	2.022135	0.198439
C	-0.227622	3.160104	-0.810725
H	1.344704	0.467478	-1.287585
C	2.459765	-1.679604	0.599398
C	4.371584	-1.108742	-0.989685
C	4.097489	0.102669	-1.475596
O	-0.956228	0.911734	-0.364873
Si	-2.476846	0.322603	0.024985
C	-2.419310	-1.495640	-0.501136
C	-2.768996	0.504695	1.865236
C	-3.777245	1.274983	-0.928879
C	-1.443174	-2.260064	0.407023
C	-1.937885	-1.602124	-1.956951
C	-3.820698	-2.115973	-0.382180

H	1.673536	2.411218	1.037095
H	-0.809622	2.366692	1.099841
H	-1.237916	3.456738	-1.101291
H	0.281201	4.029207	-0.385593
H	0.310716	2.844378	-1.708916
H	2.234260	-2.135977	1.565700
H	1.992435	-2.291245	-0.180532
H	4.928973	-1.797463	-1.618964
H	4.441293	0.345503	-2.477253
H	-3.651493	-0.067460	2.168336
H	-2.943249	1.549042	2.140926
H	-1.907915	0.140185	2.432275
H	-4.783150	0.923832	-0.678792
H	-3.638604	1.169648	-2.008719
H	-3.725531	2.340274	-0.682899
H	-1.371133	-3.307833	0.088302
H	-1.769980	-2.253599	1.451620
H	-0.440257	-1.825920	0.366183
H	-1.909114	-2.654448	-2.267738
H	-2.604228	-1.073932	-2.646964
H	-0.932118	-1.188606	-2.074733
H	-4.539970	-1.626214	-1.046292
H	-4.210100	-2.055771	0.639668
H	-3.787282	-3.177514	-0.658059
C	3.974916	-1.612763	0.378847
H	4.385460	-2.612798	0.533027
H	4.404956	-0.968029	1.154990
C	3.324496	1.169361	-0.737009
H	3.260486	2.077388	-1.341425
H	3.846033	1.436151	0.191737

Conf. 6b

C	1.137539	1.731969	0.473753
C	1.163415	0.696401	1.605259
N	1.894867	-0.163534	0.869586
C	1.968609	0.688201	-0.322553
O	0.715538	0.627626	2.735895
C	-0.233816	2.092449	-0.082747
C	-0.131313	3.039944	-1.269422
H	1.405773	0.260357	-1.156545
C	2.501635	-1.450782	1.125370
C	3.975883	-1.445674	-0.984014
C	4.190383	-0.145914	-1.202393
O	-0.878410	0.898151	-0.479613
Si	-2.394506	0.330793	-0.047898
C	-2.272625	-1.537532	-0.333223
C	-2.738931	0.744722	1.744603
C	-3.692104	1.116594	-1.146170

C	-1.245837	-2.134585	0.641855
C	-1.810117	-1.813392	-1.772856
C	-3.642915	-2.194034	-0.102870
H	1.702933	2.631924	0.728694
H	-0.798902	2.581969	0.722148
H	-1.127457	3.271000	-1.653370
H	0.355156	3.974541	-0.978330
H	0.449925	2.578556	-2.073027
H	3.393018	-1.328640	1.750714
H	1.788211	-2.071198	1.675120
H	4.687535	-2.131868	-1.436914
H	5.067824	0.110473	-1.791899
H	-3.635735	0.221676	2.091078
H	-2.911435	1.816996	1.877830
H	-1.898340	0.454065	2.380363
H	-4.694511	0.763298	-0.885148
H	-3.514725	0.889481	-2.201216
H	-3.681400	2.204659	-1.027342
H	-1.141233	-3.213439	0.469378
H	-1.543333	-1.993090	1.685738
H	-0.263356	-1.674134	0.506350
H	-1.730586	-2.894958	-1.942158
H	-2.513876	-1.415894	-2.511457
H	-0.829115	-1.369486	-1.966245
H	-4.397305	-1.816775	-0.800798
H	-4.011127	-2.026342	0.914734
H	-3.570751	-3.278930	-0.250906
C	2.876560	-2.123982	-0.191894
H	1.976047	-2.231922	-0.808769
H	3.201786	-3.140140	0.041210
C	3.380909	1.039723	-0.742142
H	3.333384	1.771448	-1.555837
H	3.899942	1.539140	0.086477

Conf. 6c

C	1.147075	1.080444	-0.707685
C	2.492654	1.658212	-0.245431
N	2.870091	0.478393	0.293775
C	1.638374	-0.240514	-0.063719
O	3.047758	2.739402	-0.312846
C	-0.124359	1.682280	-0.134023
C	-0.408044	3.069569	-0.685779
H	1.066951	-0.526388	0.824552
C	4.096198	0.012408	0.897884
C	3.839838	-2.430138	0.205518
C	2.604963	-2.550904	-0.283331
O	-1.182042	0.795386	-0.462007
Si	-2.328754	0.208841	0.614361

C	-3.362613	-0.996281	-0.414205
C	-1.481697	-0.666021	2.039877
C	-3.344455	1.634988	1.278546
C	-2.443167	-2.047308	-1.057278
C	-4.113906	-0.237905	-1.520011
C	-4.380646	-1.700878	0.498198
H	1.074318	1.023433	-1.797780
H	-0.011526	1.749285	0.958722
H	-1.324192	3.470820	-0.246572
H	0.418381	3.747297	-0.457521
H	-0.532856	3.023170	-1.771175
H	4.794538	0.851873	0.890631
H	3.907625	-0.263067	1.941056
H	4.285299	-3.294356	0.690902
H	2.096905	-3.505066	-0.174505
H	-2.221649	-0.979806	2.782682
H	-0.770160	-0.006720	2.546577
H	-0.942115	-1.556397	1.704822
H	-4.130559	1.277669	1.950814
H	-3.816567	2.198316	0.468966
H	-2.711646	2.324617	1.846411
H	-3.037463	-2.758386	-1.644993
H	-1.891643	-2.622229	-0.306173
H	-1.714910	-1.582573	-1.727954
H	-4.696790	-0.940597	-2.128940
H	-4.812411	0.496086	-1.105995
H	-3.424993	0.289164	-2.186780
H	-5.055833	-0.988705	0.984105
H	-3.889056	-2.287835	1.280544
H	-4.998827	-2.389745	-0.090902
C	4.686625	-1.180046	0.136819
H	5.677409	-1.386685	0.546664
H	4.829955	-0.881264	-0.908765
C	1.846559	-1.441707	-0.971907
H	0.867305	-1.796866	-1.301461
H	2.390303	-1.113063	-1.867415

Conf. 6d

C	1.051353	1.275220	-0.585474
C	2.252977	1.757398	0.242413
N	2.652351	0.491135	0.491550
C	1.567973	-0.149499	-0.265550
O	2.708786	2.836197	0.573099
C	-0.335718	1.643334	-0.085534
C	-0.618855	3.125495	-0.281373
H	0.900991	-0.717174	0.389501
C	3.811552	-0.071499	1.144124
C	3.900706	-2.213586	-0.239230

C	2.775462	-2.241794	-0.954272
O	-1.262701	0.850183	-0.811379
Si	-2.735915	0.241178	-0.276873
C	-2.474320	-1.414341	0.621478
C	-3.561599	1.470968	0.871416
C	-3.734691	-0.001799	-1.835751
C	-1.721161	-1.199416	1.944590
C	-1.677759	-2.378469	-0.271806
C	-3.844116	-2.043155	0.932450
H	1.147267	1.535542	-1.643662
H	-0.394489	1.405009	0.986486
H	-1.605301	3.384775	0.107122
H	0.130669	3.726214	0.239769
H	-0.586715	3.373149	-1.346044
H	4.418064	0.766417	1.494011
H	3.493044	-0.648357	2.018942
H	4.343739	-3.160432	0.057462
H	2.346916	-3.207184	-1.208630
H	-4.474985	1.042194	1.294649
H	-3.838582	2.381273	0.332439
H	-2.910912	1.753173	1.704393
H	-4.734873	-0.380483	-1.606169
H	-3.250934	-0.711738	-2.512373
H	-3.847075	0.948966	-2.364441
H	-1.575579	-2.159180	2.456250
H	-2.274409	-0.544127	2.624487
H	-0.730783	-0.760309	1.792479
H	-1.511867	-3.329167	0.250425
H	-2.210841	-2.602265	-1.201225
H	-0.699467	-1.971632	-0.540675
H	-4.413838	-2.251457	0.021610
H	-4.455303	-1.400331	1.574008
H	-3.707149	-2.995803	1.459448
C	4.624378	-0.959803	0.195274
H	5.554808	-1.230330	0.698660
H	4.898551	-0.362104	-0.682515
C	2.035455	-1.013410	-1.426185
H	1.164074	-1.297128	-2.021223
H	2.682838	-0.406858	-2.073002

Conf. 6e

C	1.114456	0.984396	-0.757434
C	2.231234	1.789637	-0.074489
N	2.794762	0.672171	0.432692
C	1.807640	-0.255844	-0.137632
O	2.535715	2.965322	0.006423
C	-0.321660	1.261950	-0.341357
C	-0.776192	2.639847	-0.794568

H	1.214487	-0.738134	0.644075
C	4.010658	0.424540	1.171381
C	4.393929	-1.940859	0.295367
C	3.286650	-2.275724	-0.368155
O	-1.138343	0.262026	-0.931276
Si	-2.166959	-0.827265	-0.171845
C	-3.713533	0.056953	0.491991
C	-2.610830	-2.043139	-1.517056
C	-1.273725	-1.693890	1.230655
C	-4.374091	0.875039	-0.628794
C	-3.347630	0.990111	1.658168
C	-4.716534	-0.994934	0.998150
H	1.195305	1.017005	-1.847960
H	-0.383184	1.203211	0.755310
H	-1.800302	2.834126	-0.470277
H	-0.124548	3.411640	-0.378222
H	-0.741124	2.703754	-1.885606
H	4.498467	1.390849	1.315310
H	3.762046	0.019567	2.158209
H	4.958299	-2.731957	0.781726
H	2.995500	-3.322126	-0.392775
H	-3.246560	-2.842762	-1.125883
H	-1.705342	-2.501731	-1.924638
H	-3.144689	-1.556194	-2.337657
H	-1.967389	-2.340513	1.776883
H	-0.845209	-0.989732	1.949749
H	-0.467277	-2.324035	0.844500
H	-5.265556	1.388188	-0.246426
H	-4.692977	0.238475	-1.460290
H	-3.698627	1.635297	-1.030832
H	-4.249830	1.482004	2.043287
H	-2.891740	0.442830	2.489181
H	-2.653404	1.779557	1.355155
H	-4.300835	-1.602863	1.808065
H	-5.037901	-1.670369	0.199214
H	-5.613271	-0.498114	1.389349
C	4.941980	-0.538457	0.426529
H	5.895339	-0.566857	0.958177
H	5.143242	-0.119945	-0.567126
C	2.389085	-1.294623	-1.083380
H	1.562999	-1.818623	-1.570083
H	2.950561	-0.774836	-1.870877

Conf. 6f

C	1.168463	1.167386	-0.770535
C	2.540430	1.695952	-0.324286
N	2.930284	0.471989	0.088127
C	1.681504	-0.207412	-0.272628

O	3.104971	2.774792	-0.322968
C	-0.069233	1.715384	-0.081189
C	-0.375895	3.145658	-0.492624
H	1.145428	-0.565068	0.611174
C	4.173447	-0.080471	0.580311
C	3.500938	-2.547401	0.248022
C	2.647556	-2.495373	-0.777393
O	-1.146505	0.860275	-0.429501
Si	-2.229103	0.172947	0.654041
C	-3.330902	-0.927609	-0.420323
C	-1.303885	-0.834631	1.936654
C	-3.192651	1.529398	1.513369
C	-2.460308	-1.929109	-1.196687
C	-4.124226	-0.067828	-1.417176
C	-4.313134	-1.697672	0.478267
H	1.043243	1.208232	-1.856492
H	0.101677	1.685634	1.005780
H	-1.265846	3.506094	0.028246
H	0.464271	3.801014	-0.249568
H	-0.558212	3.195033	-1.569701
H	4.895442	-0.163565	-0.239819
H	4.589203	0.601580	1.326434
H	3.958605	-3.514394	0.442666
H	2.504503	-3.415121	-1.340062
H	-2.002540	-1.223349	2.684056
H	-0.565511	-0.224987	2.466789
H	-0.784081	-1.685545	1.487310
H	-3.934497	1.109402	2.199511
H	-3.714075	2.165751	0.793109
H	-2.520937	2.163673	2.100767
H	-3.093017	-2.576431	-1.817071
H	-1.886147	-2.576610	-0.526050
H	-1.754533	-1.416865	-1.856785
H	-4.748607	-0.708304	-2.052993
H	-4.788819	0.636158	-0.906482
H	-3.461138	0.506512	-2.070829
H	-4.955920	-1.023090	1.053435
H	-3.792443	-2.351989	1.184684
H	-4.966739	-2.329877	-0.135606
C	3.932971	-1.453463	1.202819
H	3.205834	-1.357051	2.017846
H	4.867982	-1.764148	1.674088
C	1.841797	-1.325858	-1.280824
H	0.844171	-1.677757	-1.564791
H	2.299190	-0.932351	-2.198014

7

C	-2.632855	0.314363	-0.267916

C	-2.655202	-1.184827	0.061345
C	-1.287271	-0.917413	0.712582
N	-1.477667	0.491424	0.442302
O	-0.212433	-1.448470	-0.036034
C	0.922546	-0.673344	-0.035949
C	0.895051	0.710496	0.164299
C	-0.402605	1.452747	0.401142
C	2.124520	-1.332112	-0.285138
C	3.310078	-0.616472	-0.330903
C	3.303572	0.760771	-0.118467
C	2.102367	1.407722	0.128374
O	-3.317812	1.083052	-0.897971
H	-3.448540	-1.495948	0.739162
H	-2.609778	-1.835213	-0.811094
H	-1.152307	-1.162184	1.768304
H	-0.599077	2.156436	-0.411774
H	-0.352615	2.023563	1.333021
H	2.107002	-2.404781	-0.438760
H	4.242145	-1.134790	-0.524431
H	4.228959	1.323662	-0.145686
H	2.088638	2.481037	0.290971

Conf. 8a

C	0.179158	1.810886	0.724654
C	0.283426	0.588380	1.651438
N	1.068679	-0.076976	0.745609
C	1.132135	1.018300	-0.196746
C	2.007747	-1.162812	0.793283
C	3.289186	-0.682987	0.145937
C	3.433069	0.625874	-0.328172
O	2.433162	1.561099	-0.267692
C	4.378308	-1.550102	0.053450
C	5.585821	-1.133988	-0.483242
C	5.717534	0.177533	-0.937399
C	4.647445	1.053154	-0.862864
O	-0.142724	0.275000	2.734623
H	0.776428	0.767547	-1.198003
O	1.557992	-2.309370	0.105236
C	-1.211016	2.118472	0.185468
C	-1.188299	3.238992	-0.843690
H	0.657506	2.695431	1.148705
O	-1.715090	0.930473	-0.390075
Si	-3.228639	0.234727	-0.172803
C	-2.943503	-1.608264	-0.501329
C	-3.802938	0.545927	1.581406
C	-4.435120	0.976331	-1.395942
C	-2.134307	-2.213454	0.656615
C	-2.158645	-1.787101	-1.810739

C	-4.294089	-2.333767	-0.612322
H	2.181359	-1.399556	1.848368
H	4.264368	-2.565478	0.416889
H	6.420100	-1.822206	-0.544928
H	6.657293	0.517663	-1.357098
H	4.727986	2.073328	-1.218900
H	0.854901	-2.718470	0.617496
H	-1.829653	2.424285	1.039360
H	-2.198771	3.428701	-1.211641
H	-0.800860	4.160872	-0.402782
H	-0.557905	2.965054	-1.694052
H	-4.679282	-0.069651	1.807258
H	-4.086998	1.591415	1.732826
H	-3.016610	0.296226	2.299609
H	-4.504865	2.059404	-1.255947
H	-4.124037	0.786669	-2.427294
H	-5.436738	0.557033	-1.260170
H	-1.902559	-3.265756	0.447427
H	-2.684896	-2.176982	1.601452
H	-1.191015	-1.678629	0.799083
H	-2.002228	-2.854504	-2.012400
H	-2.693958	-1.365142	-2.667820
H	-1.177806	-1.306960	-1.755070
H	-4.132594	-3.408632	-0.762069
H	-4.898531	-2.216765	0.293634
H	-4.882622	-1.968119	-1.459537

Conf. 8b

C	0.189347	1.329370	1.119475
C	0.325204	-0.128835	1.585265
N	1.165018	-0.422677	0.542504
C	1.226585	0.935192	0.045090
C	2.156628	-1.428896	0.282848
C	3.443537	-0.719031	-0.079781
C	3.547567	0.676380	-0.059717
O	2.501671	1.501919	0.261025
C	4.575760	-1.468428	-0.400913
C	5.786275	-0.857045	-0.684307
C	5.877041	0.533591	-0.645251
C	4.764098	1.297720	-0.336937
O	-0.101212	-0.799581	2.491567
H	0.941094	1.044336	-1.002331
O	1.798806	-2.287084	-0.777750
C	-1.180261	1.774343	0.622736
C	-1.143032	3.212502	0.123280
H	0.597352	2.030822	1.849096
O	-1.566774	0.895829	-0.416502
Si	-3.109018	0.351740	-0.810501

C	-3.488280	-1.223643	0.177092
C	-4.366537	1.688997	-0.434696
C	-3.027351	-0.006040	-2.640915
C	-3.569098	-0.900025	1.677848
C	-2.378269	-2.260663	-0.059419
C	-4.832925	-1.810811	-0.283856
H	2.287791	-2.004039	1.205714
H	4.493196	-2.549541	-0.420824
H	6.654381	-1.456574	-0.930150
H	6.818570	1.024986	-0.862139
H	4.812724	2.379821	-0.310730
H	1.098273	-2.870843	-0.474544
H	-1.876945	1.702921	1.468596
H	-2.129963	3.515576	-0.230525
H	-0.840742	3.890463	0.925336
H	-0.434553	3.309284	-0.704055
H	-5.381880	1.310289	-0.585464
H	-4.229724	2.548426	-1.097055
H	-4.294252	2.040858	0.598603
H	-2.786505	0.905393	-3.195330
H	-2.260437	-0.752804	-2.864507
H	-3.985390	-0.383117	-3.010536
H	-3.805703	-1.809281	2.245532
H	-4.351665	-0.165595	1.894234
H	-2.619703	-0.517222	2.062780
H	-2.575068	-3.165388	0.529750
H	-2.319112	-2.558792	-1.111109
H	-1.399576	-1.875301	0.238663
H	-5.056704	-2.723243	0.283218
H	-5.661950	-1.114413	-0.121555
H	-4.820039	-2.079376	-1.344948

Conf. 8c

C	0.170405	1.397691	-0.948316
C	1.333186	2.341759	-0.590864
N	1.926887	1.373015	0.179756
C	0.965170	0.343063	-0.155298
C	3.263742	1.118069	0.643473
C	3.629429	-0.292094	0.230902
C	2.773913	-1.080240	-0.546543
O	1.540934	-0.653751	-0.968583
C	4.871921	-0.813621	0.592452
C	5.266568	-2.078091	0.186361
C	4.408178	-2.844411	-0.600608
C	3.166552	-2.350465	-0.964065
O	1.650437	3.476229	-0.843627
H	0.480015	-0.120167	0.706654
O	3.387803	1.227145	2.043282

C	-1.211657	1.753878	-0.418865
C	-1.809630	2.940603	-1.156824
H	0.130499	1.163674	-2.014411
O	-2.022496	0.605134	-0.577034
Si	-3.019298	-0.030905	0.620736
C	-3.802006	-1.556765	-0.175382
C	-1.986718	-0.486881	2.117174
C	-4.292043	1.251447	1.111547
C	-2.700667	-2.509568	-0.667964
C	-4.680073	-1.138805	-1.365646
C	-4.671469	-2.281041	0.866533
H	3.923226	1.839555	0.149443
H	5.532598	-0.204951	1.199758
H	6.236054	-2.464620	0.476548
H	4.705463	-3.834314	-0.927200
H	2.481331	-2.934456	-1.566887
H	3.364516	2.158200	2.279537
H	-1.116201	2.008750	0.647395
H	-2.793867	3.178930	-0.748506
H	-1.167183	3.819121	-1.057171
H	-1.919546	2.704843	-2.218662
H	-2.633977	-0.816100	2.935830
H	-1.412279	0.370480	2.481794
H	-1.287351	-1.298065	1.896116
H	-3.806540	2.132106	1.544054
H	-4.881644	1.578525	0.250766
H	-4.978391	0.850633	1.863792
H	-3.151345	-3.397358	-1.129282
H	-2.063069	-2.854050	0.152454
H	-2.060559	-2.030873	-1.414363
H	-5.122315	-2.026064	-1.836090
H	-5.502758	-0.487327	-1.054508
H	-4.100129	-0.610851	-2.128306
H	-5.140306	-3.163884	0.414500
H	-4.081373	-2.624435	1.722240
H	-5.475145	-1.640977	1.245187

Conf. 8d

C	0.178132	1.155212	-1.010004
C	1.139256	2.243182	-0.493747
N	1.833120	1.323979	0.252277
C	1.078097	0.183353	-0.223128
C	3.158529	1.250491	0.804802
C	3.775906	-0.051076	0.338609
C	3.116654	-0.904381	-0.552940
O	1.863790	-0.646917	-1.047445
C	5.058069	-0.396193	0.767140
C	5.680146	-1.549620	0.317527

C	5.015096	-2.381199	-0.582205
C	3.738578	-2.062861	-1.014378
O	1.279069	3.430797	-0.641177
H	0.619028	-0.411979	0.568170
O	3.166461	1.278041	2.213976
C	-1.285817	1.251338	-0.598808
C	-1.962886	2.432365	-1.275959
H	0.264476	1.004442	-2.088443
O	-1.913291	0.039752	-0.973559
Si	-2.759662	-1.034975	0.007862
C	-4.438081	-0.303130	0.516808
C	-2.979029	-2.546400	-1.063806
C	-1.746217	-1.437052	1.533387
C	-5.204635	0.175113	-0.726426
C	-4.247057	0.874771	1.486672
C	-5.265448	-1.392923	1.222023
H	3.724569	2.100057	0.408014
H	5.567225	0.262406	1.461979
H	6.676716	-1.799315	0.661045
H	5.491372	-3.285252	-0.943824
H	3.201410	-2.701011	-1.705861
H	2.975243	2.174534	2.502318
H	-1.332423	1.384339	0.491843
H	-3.003215	2.512291	-0.956588
H	-1.450102	3.364201	-1.026058
H	-1.943004	2.301220	-2.361068
H	-3.465088	-3.351936	-0.505829
H	-2.006466	-2.911799	-1.405242
H	-3.589454	-2.327519	-1.944121
H	-0.864587	-2.028557	1.270150
H	-1.414163	-0.539286	2.063143
H	-2.341485	-2.028887	2.235389
H	-6.174717	0.595444	-0.432614
H	-5.400198	-0.647074	-1.422167
H	-4.657466	0.949324	-1.271265
H	-5.223022	1.277987	1.784900
H	-3.725074	0.569393	2.398860
H	-3.682411	1.696605	1.036357
H	-6.235101	-0.983189	1.531375
H	-5.464043	-2.244441	0.563925
H	-4.768574	-1.769534	2.121833

Conf. 8e

C	0.035102	1.543326	-0.804840
C	1.063653	2.436793	-0.085149
N	1.691483	1.340120	0.450351
C	0.863143	0.374189	-0.239357
C	3.004090	1.063796	0.967998

C	3.532806	-0.155322	0.242013
C	2.821342	-0.764209	-0.797547
O	1.592477	-0.325451	-1.220443
C	4.785258	-0.667500	0.581806
C	5.328335	-1.747233	-0.095500
C	4.612255	-2.333096	-1.138231
C	3.363448	-1.846800	-1.487154
O	1.281931	3.617481	0.013124
H	0.360831	-0.336950	0.419335
O	3.001796	0.800750	2.352729
C	-1.420169	1.655431	-0.373044
C	-2.019712	2.980032	-0.822068
H	0.119819	1.608228	-1.891999
O	-2.104608	0.560161	-0.952411
Si	-3.417802	-0.282973	-0.317930
C	-2.803192	-1.646189	0.855513
C	-4.552743	0.896815	0.593187
C	-4.261816	-1.020223	-1.810368
C	-2.132381	-1.033935	2.096226
C	-1.802409	-2.554723	0.123270
C	-4.000494	-2.496746	1.315686
H	3.631011	1.935073	0.750554
H	5.334596	-0.198902	1.390759
H	6.303121	-2.129220	0.182672
H	5.026525	-3.176986	-1.677818
H	2.787105	-2.294304	-2.288084
H	2.866653	1.629329	2.820150
H	-1.465907	1.595370	0.723726
H	-3.054600	3.063613	-0.486694
H	-1.451709	3.816542	-0.407454
H	-1.998740	3.048451	-1.912988
H	-5.365196	0.345605	1.076031
H	-5.001702	1.615308	-0.098266
H	-4.024319	1.456763	1.370368
H	-4.594032	-0.228145	-2.487282
H	-3.586745	-1.679162	-2.363262
H	-5.139383	-1.603280	-1.516411
H	-1.787871	-1.828670	2.769642
H	-2.823455	-0.400215	2.660654
H	-1.258971	-0.426270	1.842078
H	-1.416456	-3.321222	0.806855
H	-2.271215	-3.071837	-0.719754
H	-0.947469	-1.996050	-0.267963
H	-3.657080	-3.291301	1.989957
H	-4.740425	-1.902687	1.861347
H	-4.507420	-2.978264	0.473752

Conf. 9a

C	1.238239	-2.054303	0.513057
C	0.921670	-1.056755	1.640226
N	0.036395	-0.378709	0.840234
C	0.157160	-1.285768	-0.278905
C	-1.082157	0.491047	1.068907
C	-2.268394	-0.092495	0.330618
C	-2.181157	-1.298835	-0.371909
O	-1.030814	-2.028510	-0.471975
C	-3.493438	0.570281	0.386783
C	-4.605692	0.030542	-0.231659
C	-4.526308	-1.178685	-0.915498
C	-3.311603	-1.836692	-0.984536
O	1.271239	-0.885518	2.779977
H	0.452543	-0.808728	-1.214897
Br	-6.269779	0.950448	-0.140684
O	-0.868199	1.802959	0.602305
C	2.656149	-2.018235	-0.040418
C	2.818097	-2.931600	-1.246728
O	2.941343	-0.680960	-0.398187
Si	4.321000	0.187750	0.009806
C	3.977991	1.916181	-0.676517
C	5.814134	-0.617155	-0.783888
C	4.527304	0.203784	1.870301
C	3.843485	1.852317	-2.206340
C	2.667924	2.454676	-0.078132
C	5.136675	2.856974	-0.307684
H	0.923548	-3.069939	0.758958
H	-1.275135	0.497731	2.147007
H	-3.559909	1.508717	0.923629
H	-5.399640	-1.601801	-1.395437
H	-3.220322	-2.775081	-1.517645
H	-0.219095	2.226826	1.170598
H	3.327644	-2.357623	0.760257
H	3.839114	-2.865998	-1.628429
H	2.612957	-3.970814	-0.977785
H	2.132627	-2.634123	-2.045008
H	5.961194	-1.625876	-0.384676
H	6.724229	-0.045342	-0.578315
H	5.696194	-0.695970	-1.868007
H	3.665986	0.662177	2.362378
H	4.637736	-0.811099	2.264083
H	5.425069	0.764184	2.149093
H	4.768839	1.511229	-2.681350
H	3.615992	2.848215	-2.607152
H	3.038386	1.176910	-2.510404
H	2.724905	2.540718	1.012186
H	2.455621	3.454653	-0.477300
H	1.822036	1.806157	-0.323248

H	5.251887	2.958895	0.776098
H	4.951539	3.858732	-0.715036
H	6.090549	2.508576	-0.717621

Conf. 9b

C	-1.318065	1.657660	-0.897217
C	-0.357406	2.806443	-0.541207
N	0.460005	1.950444	0.157780
C	-0.290171	0.759889	-0.184869
C	1.843920	1.960356	0.546717
C	2.464657	0.671064	0.049281
C	1.745895	-0.245659	-0.724245
O	0.434263	-0.070688	-1.065803
C	3.804466	0.410341	0.332984
C	4.410088	-0.730878	-0.159286
C	3.701413	-1.635095	-0.944365
C	2.368817	-1.388516	-1.220985
O	-0.289753	3.990150	-0.751337
H	-0.631664	0.175580	0.672458
Br	6.240640	-1.068782	0.239075
O	2.025439	2.038081	1.940610
C	-2.718525	1.691095	-0.302314
C	-3.585275	2.754367	-0.957448
O	-3.264639	0.401473	-0.493653
Si	-4.140343	-0.481424	0.638629
C	-4.333880	-2.188060	-0.156123
C	-5.791264	0.350564	0.937617
C	-3.179231	-0.571848	2.245411
C	-5.035160	-2.053033	-1.517175
C	-2.949579	-2.825147	-0.359219
C	-5.177835	-3.086917	0.763092
H	-1.357115	1.462426	-1.971148
H	2.314686	2.817889	0.054341
H	4.361161	1.114897	0.938622
H	4.179109	-2.526339	-1.330983
H	1.790086	-2.079970	-1.820956
H	1.830677	2.935003	2.225295
H	-2.630891	1.911725	0.771623
H	-4.576821	2.765942	-0.500493
H	-3.134452	3.742782	-0.838054
H	-3.694766	2.544981	-2.024809
H	-5.649741	1.336780	1.390787
H	-6.410611	-0.241319	1.618344
H	-6.341589	0.482602	0.001913
H	-2.209880	-1.059891	2.111746
H	-3.007015	0.422865	2.667075
H	-3.743629	-1.146033	2.986846
H	-6.032099	-1.611560	-1.418057

H	-5.156330	-3.041337	-1.978425
H	-4.456212	-1.430222	-2.204854
H	-2.432599	-2.983024	0.592562
H	-3.053455	-3.804299	-0.843487
H	-2.309891	-2.203797	-0.992923
H	-4.709748	-3.222761	1.743548
H	-5.292809	-4.080695	0.312759
H	-6.181677	-2.679944	0.920967

Conf. 9c

C	-1.200493	1.403823	-0.966438
C	-0.362458	2.562017	-0.393009
N	0.478808	1.682144	0.244175
C	-0.146604	0.496868	-0.303450
C	1.830549	1.741613	0.729921
C	2.587667	0.586555	0.107688
C	1.996185	-0.268335	-0.827516
O	0.696183	-0.149460	-1.232910
C	3.926392	0.393834	0.445647
C	4.654937	-0.619099	-0.149667
C	4.072848	-1.460238	-1.092963
C	2.742250	-1.281447	-1.425809
O	-0.381434	3.764939	-0.440261
H	-0.483881	-0.221920	0.445078
Br	6.482043	-0.864783	0.324553
O	1.918877	1.632741	2.130558
C	-2.643339	1.271740	-0.494937
C	-3.504073	2.385678	-1.069584
O	-3.114356	0.008706	-0.923061
Si	-3.763601	-1.229776	0.015946
C	-5.524633	-0.786586	0.575878
C	-2.671653	-1.536317	1.509803
C	-3.766886	-2.709141	-1.121295
C	-5.491940	0.341485	1.620082
C	-6.364622	-0.343842	-0.632608
C	-6.177037	-2.029151	1.207693
H	-1.146402	1.353802	-2.056317
H	2.255919	2.693427	0.394546
H	4.384258	1.050494	1.175209
H	4.646137	-2.252001	-1.558184
H	2.260899	-1.928491	-2.148758
H	1.625205	2.460271	2.520865
H	-2.658567	1.334579	0.602777
H	-4.525705	2.313281	-0.693186
H	-3.100137	3.362809	-0.794125
H	-3.530187	2.310700	-2.159871
H	-1.727977	-2.004247	1.215090
H	-3.171649	-2.216609	2.205974

H	-2.445196	-0.617301	2.058384
H	-4.453938	-2.570398	-1.960520
H	-2.764690	-2.877678	-1.525163
H	-4.069679	-3.611199	-0.581731
H	-4.930866	0.050975	2.513761
H	-6.512496	0.589717	1.937679
H	-5.043524	1.258549	1.226109
H	-6.438337	-1.134383	-1.386175
H	-7.385084	-0.097471	-0.313237
H	-5.943953	0.540612	-1.118718
H	-6.259610	-2.852748	0.491734
H	-7.191290	-1.786580	1.548694
H	-5.618696	-2.391096	2.077028

Conf. 9d

C	-1.409962	1.723714	-0.780798
C	-0.549215	2.766213	-0.040471
N	0.286169	1.784981	0.435887
C	-0.362238	0.711043	-0.283989
C	1.638393	1.739485	0.920866
C	2.365274	0.667504	0.134552
C	1.757973	-0.011489	-0.926602
O	0.463650	0.206417	-1.310336
C	3.693817	0.382900	0.446168
C	4.398959	-0.544963	-0.297723
C	3.802814	-1.206334	-1.367050
C	2.480965	-0.938161	-1.674335
O	-0.552287	3.961014	0.107009
H	-0.702432	-0.112519	0.346176
Br	6.212650	-0.918758	0.142580
O	1.723113	1.428128	2.291209
C	-2.845223	1.538135	-0.307961
C	-3.702207	2.737459	-0.685735
O	-3.326792	0.352017	-0.912046
Si	-4.433070	-0.745813	-0.273044
C	-3.527252	-2.012487	0.818483
C	-5.187834	-1.575865	-1.765020
C	-5.725919	0.167210	0.730371
C	-2.402060	-2.686085	0.016569
C	-2.937304	-1.331957	2.064672
C	-4.524182	-3.091726	1.277096
H	-1.372060	1.847988	-1.865286
H	2.090120	2.717871	0.726004
H	4.162347	0.900438	1.274260
H	4.359279	-1.928777	-1.950561
H	1.989381	-1.446782	-2.494535
H	1.467053	2.203222	2.798264
H	-2.840237	1.431922	0.786249

H	-4.721042	2.608294	-0.317054
H	-3.290301	3.652487	-0.253130
H	-3.734184	2.846968	-1.773004
H	-5.682236	-0.837355	-2.402175
H	-5.934754	-2.315613	-1.462738
H	-4.427774	-2.087402	-2.362044
H	-5.275053	0.800119	1.500471
H	-6.341372	0.801422	0.086317
H	-6.389508	-0.543048	1.232536
H	-2.796739	-3.246135	-0.836840
H	-1.854422	-3.393139	0.651998
H	-1.681508	-1.960605	-0.371417
H	-3.714364	-0.866863	2.679153
H	-2.419830	-2.070813	2.689139
H	-2.207595	-0.557391	1.811788
H	-5.340342	-2.670212	1.872398
H	-4.011104	-3.832029	1.903523
H	-4.964543	-3.627219	0.430379

Conf. iii-a

C	2.38719500	-0.85831400	0.87760100
C	1.87612100	-0.60661700	-0.55394300
N	1.81684800	0.82135500	-0.27679400
O	0.62117700	-1.23688300	-0.84686300
C	-0.51739400	-0.63804600	-0.39471300
C	-0.61094700	0.75525600	-0.34843700
C	0.62749400	1.51547100	-0.74637800
C	-1.59557700	-1.43891500	-0.03797200
C	-2.78252100	-0.84124100	0.36661900
C	-2.88756200	0.54418800	0.43371100
C	-1.79756200	1.33355400	0.08312800
H	3.45695500	-1.05933500	0.93005200
H	1.83500100	-1.62630300	1.41769000
H	2.51343000	-0.89316800	-1.39224900
H	0.61926000	2.53127500	-0.34336800
H	0.69324900	1.59866800	-1.83711600
H	-1.49104700	-2.51613500	-0.09023300
H	-3.62743400	-1.46341200	0.63976300
H	-3.81136300	1.00651200	0.76112000
H	-1.86929400	2.41590700	0.13731000
C	1.99498400	0.60391700	1.17403800
H	2.76152500	1.24469400	1.61465900
H	1.06698400	0.69136200	1.75291600

Conf. iii-b

C	2.95996900	-0.85053400	-0.03841900
C	1.58313600	-0.59863100	-0.65468800
N	1.71922400	0.80987000	-0.35495900

O	0.58546700	-1.26393800	0.13102000
C	-0.63059800	-0.64663800	0.07589000
C	-0.71889800	0.74261200	-0.01868000
C	0.53702700	1.58144200	-0.00501000
C	-1.77583000	-1.43487700	0.13816000
C	-3.02466800	-0.83404500	0.10204900
C	-3.13300700	0.54921300	-0.01619900
C	-1.98332900	1.32331500	-0.08155600
H	3.75557800	-0.89776200	-0.78072400
H	3.00700800	-1.70596800	0.63247300
H	1.38924300	-0.83644500	-1.70553400
H	0.67548000	1.99153200	1.00405400
H	0.41619800	2.43671600	-0.67939800
H	-1.66420300	-2.51002000	0.21385500
H	-3.91690100	-1.44777700	0.15335300
H	-4.10825600	1.01963600	-0.05972900
H	-2.06183400	2.40283900	-0.17265700
C	2.80053300	0.54528200	0.60626100
H	3.64640100	1.23316500	0.53562100
H	2.45697500	0.49366900	1.64839600
